# The use of minerals, plants and burnt materials in ancient medicine: Approaches to working recipes in John the Physician's *Therapeutics* from late 13th century Cyprus

**DOI:** 10.1016/j.pgeola.2026.101172

**Published:** 2026-03

**Authors:** Andrew C. Scott, Rebecca Lazarou, Robert Allkin, Mark Nesbitt, Andreas Lardos, Efraim Lev, Sharon Gibbons, Nathali V. Grassineau, James Brakeley, Barbara Zipser

**Affiliations:** a Department of Earth Science, Royal Holloway University of London, Egham, TW20 0EX, United Kingdom; b Science Directorate, Royal Botanic Gardens, Kew, Richmond, TW9 3AE, United Kingdom; c Biodiversity Informatics and Spatial Analysis, Royal Botanic Gardens, Kew, Richmond, TW9 3AB, United Kingdom; d Phytopharmacy and Natural Product Chemistry, ZHAW Zurich University of Applied Sciences, Life Sciences and Facility Management, Gruental, Wädenswil, CH-8820, Switzerland; e Department of Israel Studies, The Interdisciplinary Center for the Broader Application of Genizah Research, University of Haifa, 3498838, Israel; f Department of History, Royal Holloway University of London, Egham, TW20 0EX, United Kingdom

**Keywords:** History of medicine, Pharmaceutical ingredients, Burnt substances, Medical treatments

## Abstract

We have undertaken an innovative multidisciplinary approach towards the identification of pharmaceutical ingredients used in Byzantine Greece, with a particular focus on popular medicine in late 13th century Cyprus. Our case study is based on one source, John the Physician's *Therapeutics* (JC), along with a comparative study of other scholarly and non-scholarly texts. Our main goal was to develop a new, documented and transferable methodology to address a key, unresolved challenge when working with such texts, namely our ability to identify with confidence the individual ingredients, primarily plants, minerals and burnt materials, cited. This is an essential step in analysis of ancient pharmacy. Practical research focused on the understudied burnt substances and minerals that were added to medication. Ingredients identified have been mapped onto their current pharmaceutical uses thus exploring potential interest to pharmacological research.

The main approaches in relation to minerals and burnt substances include a comparison between JC and the ancient Greek handbook, *De Materia Medica*, written by Pedanius Dioscorides to see if there are likely candidates for materials; a consideration of the potential minerals available from Cyprus; the significance of mineral elements in modern medicine and a reconstruction of some of the recipes suggested by JC.

In this paper we describe a series of experiments reconstructing the use of burnt material in the recipes and consider their potential pharmaceutical use and potential efficacy. We conclude that at least some of the recipes had some potential practical medicinal value.

## 1 General introduction

Understanding ancient medical texts and pharmaceutical recipes is fraught with difficulty (Lev, 2010). Many have assumed that the published identifications of the ingredients are correct but the translation of any ancient text with regard to plants, minerals and burnt substances raises many issues, some of which have never been adequately assessed. The ancient Greek handbook *De Materia Medica* (Osbaldeston and Wood, 2000; Beck, 2005), written by Pedanius Dioscorides in the first century CE, has been highly influential on subsequent medical texts in Europe and Asia to the current day (Andrade, 2015; Griebler, 2019). To apply new methodologies to the identification of its ingredients we have chosen as a case study John the Physician's 13th century CE Byzantine Greek text *Therapeutics* (Zipser, 2009; Zipser et al., 2023a), which itself is based on the *De Materia Medica* in terms of the ingredients used. Our main goal was to develop a new, documented and transferable methodology to address a key, unresolved challenge when working with such texts, namely our ability to identify with confidence the individual ingredients, primarily plants, minerals and burnt materials, cited. This is an essential step in analysis of pharmaceutical practices. In the first part of our study we focused on botanical components (Zipser et al., 2023a; Lardos et al., 2024). We developed an iterative experimental approach, with six stages and including comparative analyses based on statistical evaluation of botanical descriptions and information about medicinal uses drawn from both historical and modern sources, enabling detection of the most likely candidate species.

Mineral and burnt substance identification represents a different order of problem. Whereas the plants of most regions have been described and published in regional floras, most minerals are not so easily identified on sight. In some cases colour and form are reliable, as in the case of native elements such as gold, silver, copper and sulphur. More often, secure identification of minerals needs knowledge of chemical composition and crystal structure, not known to anyone in the past, nor to non-specialists today (Zipser et al., 2023b). Our paper falls into two main parts. In 4, 5, 6, 7 we survey the minerals present in Cyprus, the presumed origin of JC (see Section 2), and thus most likely to have been used, examine the significance of elements in medicine, and finally synthesise this evidence with that of philology to propose identification of minerals and related substances in our study texts. In Section 8 we describe a series of experiments carried out to investigate why the medicinal recipes sometimes recommend burning minerals and plant and animal materials. However, we begin by describing our study texts and explaining how we extract mineral names and recipes from them.

## 2 The source texts

Our core primary sources are very different in style, intellectual approach and structure. Our first, *Περὶ ὕλης ἰατρικῆς (peri hulēs iatrikēs)*, commonly known under its Latin title *De Materia Medica* (DMM), was written by Dioscorides in the second half of the first century CE (Wellmann, 1907; Osbaldeston and Wood, 2000; Beck, 2005). Dioscorides was born and raised in Anazarbus, which is in modern terms in southern Turkey, near the border with Syria. In antiquity, this area was known as Cilicia. We do not have much reliable data on Dioscorides' life or career. DMM is a large and comprehensive reference work on the pharmaceuticals that were deemed relevant in the Graeco-Roman world of this time. It is divided into five books, with each book being subdivided into encyclopaedia style entries devoted to a given ingredient. Most entries concern plants, but some others are devoted to animals, animal products or minerals. The entries do not have a uniform style, apart from the fact that they all start with a headword for the ingredient. Some entries contain a description of the product, for instance what a certain plant species looked like, whilst others only mention what a given ingredient is used for. There is a large amount of granular data hidden in these entries, that allows us to reconstruct certain aspects of ancient medicine. DMM became a seminal text. Later medical or pharmaceutical writers largely follow its nomenclature, at least in Europe.

On the other side of the spectrum, we have a highly unusual text dating to the late 13th century CE, which we call JC, short for John the Physician commentary version. It is a vernacular translation of an earlier medical text to which was added a commentary (Zipser, 2009). It is also one of the earliest longer witnesses of a dialect that would some centuries later be standardised as modern Greek. We do not have any data on the author of this text, other than what we can extract from the text itself. He was evidently a physician who due to his rather humble circumstances also doubled as a pharmacist. He could read ancient Greek, which was the language used in writing at his time, but he chose to write in dialect. It appears that he was trying to reach audiences who could read, but who were not educated enough to read learned texts. The difference between his dialect and the dead language used in writing was quite significant. For several reasons, we conclude that JC was written in Cyprus (Zipser, 2009).

JC has been chosen for our analysis as it reflects an actual medical practice for patients with limited means. A substantial part of the materia medica mentioned in this text would have been locally available. The text was also reduced to the bare minimum, forming a stark contrast to learned manuals of ancient and medieval times that aimed to compile as much as possible. Often they tend to dwell on complex and expensive preparations, but it may be doubted whether these had been tried and tested. On the other hand, the preparations in JC are tried and tested, and they have, beneath the rough dialectal appearance, a clearly defined pharmaceutical system. In brief, JC is a text that provides guidelines for someone who does not have many resources, but who can gather and manufacture simple pharmaceuticals locally.

Whilst there is nothing to suggest that JC had a copy of DMM to his disposal, it is well-demonstrated that the DMM was the standard reference source for materia medica in Byzantine times (Riddle, 1984, Riddle, 1985), but this could manifest directly or indirectly. In essence, DMM created a set terminology for medicinal herbs, which was then applied in oral and written traditions throughout the Greek world, and sometimes also beyond. Given that many of the terms for ingredients used in JC are also used in DMM, we are confident that JC must also have come into existence in a context where the DMM terminology was used. We therefore took as our evidence the terms used, and the limited further information in JC, and the much fuller information given for the same ingredients in DMM.

## 3 Textual analysis

We assigned all materia medica mentioned in our JC text a unique identifier. Plants were listed as JCP_ followed by a running number, and minerals as JCM_ followed by a running number. Details of this process can be found in Zipser et al. (2023a: 4–5).

Given that Greek words inflect, and that our primary source is written in a dialect that does not have a standardised spelling, we then set up a hierarchy in which all inflected forms and spelling variants of a given word were standardised under a single term, known as a lemma by philologists. Each lemma was assigned the tag JCLP_ or JCLM_ followed by a running number. To borrow a comparison from contemporary English, the words 'apple' and 'apples' would have the same JCLP_ tag. The lemmas are given for minerals in Table 1; for plants see Zipser et al. (2023a; Lardos et al., 2024).

**Table 1:** Application of lemma tags (JCCLM_) for the characterisation of materials in the JC text. The translation refers to the term used by Beck (2005); succeeding columns list the one or more unique identifiers (JCM_) applied at each mention of a material.

Lemma tag	Translation	CF	IT	TM	Greek	IT	TM	Greek	IT	TM	Greek	IT	TM	Greek
JCLM_001	Hematite (*haimatitēs*)	**2**	JCM_1408	1	αἱματίθην									
JCLM_027	Iron (*sidēron*)	**4**	JCM_2569	1	σίδηρον	JCM_2568	1	σιδέρου	JCM_0916	3	σιδήρου			
JCLM_028	Fe_2_O_3_ in modern Greek (*skouria*)	**3**	JCM_2598	1	σκουρέαν	JCM_2599	1	σκουρίαν						
JCLM_026	A stone that strikes fire (*pyritēs*)	**3**	JCM_2485	1	χαλκόν									
JCLM_020	Magnet (*magnētis*)	**3**	JCM_2162	1	μαγνήτιν									
JCLM_039	Literally copper/bronze stone	**1**	JCM_2840	1	χαλικολίθαρον									
JCLM_041	Copper or bronze (*khalkos*)	**3**	JCM_2876	1	χαλκόν									
JCLM_014	Blue vitriol (*kalkanthos, khalkanthos*)	**2**	JCM_1938	1	καλακάνθην									
JCLM_006	Yellow orpiment (*arsenikin*)	**2**	JCM_0436	7	ἀρσενίκιν									
JCLM_029	Antimony (*stibē*)	**2**	JCM_2636	1	στίβην	JCM_2637	1	στιβῆς						
JCLM_043	Lead white (*psimmithion*)	**3**	JCM_0268	13	ψιμμίθιον	JCM_0473	7	ψιμμίθι						
JCLM_018	Litharge (*litharguron*)	**3**	JCM_0125	27	λιθάργυρον									
JCLM_024	Lead (*molubdin*)	**4**	JCM_2230	1	μολύβδιν	JCM_2231	1	μολύβδινον	JCM_2232	1	μολύβιν			
JCLM_031	Either “bright red mineral colourant from red lead” or alternatively a misspelled word that means “silk”. We think it is the former (*surikon*)	**1**	JCM_1323	2	συρικόν									
JCLM_030	Astringent substance containing either alum or ferrous sulphate (*stupteria*)	**2**	JCM_2652	1	στυπτερίαν	JCM_2653	1	στυπτηρίαν						
JCLM_035	Mercury (*hugrarguron*)	**4**	JCM_2765	1	ὑγράργυρον									
JCLM_037	Mercury (*hudrarguros*)	**4**	JCM_0470	7	ὑδράργυρον									
JCLM_041	Gold	**4**	JCM_2876	1	χρυσάφιν									
JCLM_012	Sulphur (*theion*)	**4**	JCM_1873	1	θεῖον									
JCLM_005	Without fire (*apuron*, used to describe a type of sulphur)	**4**	JCM_1524	1	ἄπυρον									
JCLM_033	Probably sulphur (*teaphin*)	**3**	JCM_0188	18	τεάφην	JCM_0385	9	τεάφιν	JCΜ_2702	1	τεάφης			
JCLM_007	Lime (*asbestin*)	**2**	JCM_0437	7	ἀσβέστιν	JCM_1546	1	ἀσβέστην						
JCLM_021	Marble (*marmaron*)	**3**	JCM_2180	1	μάρμαρον									
JCLM_015	Coal	**4**	JCM_0448	1	καρβούνια	JCM_1959	1	καρβουνίων						
JCLM_009	Lump of earth (*bolos*)	**4**	JCM_0439	7	βῶλον									
JCLM_019	Stone (*lithos*)	**4**	JCM_2131	1	λίθος	JCM_2130	1	λίθον	JCM_1184	2	λιθάριν	JCM_2129	1	λιθάρια
JCLM_025	Of rock	**4**	JCM_1261	2	πετραίους									
JCLM_034	Glass	**4**	JCM_2764	1	ὑάλιν	JCM_0943	3	ὑέλινον						
JCLM_002	Salt	**4**	JCM_0116	29	ἅλας	JCM_0433	7	ἅλατος						
JCLM_004	Salt water	**4**	JCM_0969	2	ἅλμην									
JCLM_023	Sodium carbonate (*nitron*)	**3**	JCM_0416	8	νίτρον									
JCLM_022	Water	**4**	JCM_0018	215	νερόν	JCM_0197	17	νεροῦ	JCM_1218	2	νερά			
JCLM_036	Water	**4**	JCM_0539	6	ὕδωρ	JCM_0230	15	ὕδατος	JCM_2768	1	ὕδατι			
JCLM_038	Hail stone	**4**	JCΜ_2838	1	χαλάζιν									
JCLM_003	A type of water of volcanic origin	**2**	JCM_1428	1	ἄλβουλα									
JCLM_008	Rain	**3**	JCM_0772	3	βροχῆς									
JCLM_010	Either an unusual word for dew, or alternatively rosewater	**1**	JCM_0785	3	δροσᾶτον									
JCLM_011	Sea water	**4**	JCM_1867	1	θαλασσίαν									
JCLM_016	Pumice stone (*kissērin*)	**3**	JCM_1142	2	κισσήριν									
JCLM_032	Type of medication with a certified seal on it, commonly clay	**4**	JCM_1324	2	σφραγίδα									
JCLM_017	Lemnian earth	**4**	JCM_2126	1	σφραγίδα									

We have then considered if any of the minerals may have been mentioned or used in Dioscorides (Table 2). It is clear, therefore that even a simple linking of words in the John the Physician text with those in Dioscorides is very difficult. The questions then arise — which of these minerals or other substances occur in both texts, and how accurate is not just the translation of the words but also the identification of the material?

**Table 2:** Minerals cited in Dioscorides (data from Beck, 2005). Columns show the reference to the relevant part of the text, the Greek term used by Dioscorides, the Greek term written in Roman script, and Beck's translation.

Dioscorides Ref	Greek	Greek (Roman alphabet)	Translators identification
V, 74	καδμεία	*kadmeia*	Calamine
V, 75	πομφόλυξ	*pompholux*	Pompholyx
V, 76	κεκαυμένος χαλκός	*kekaumenos khalkos*	Burned copper
V, 77	χαλκοῦ ἄνθος	*khalkou anthos*	Flower of copper
V, 78	λεπίς	*lepis*	Flake [of ore]
V, 79	ἰός ξυστός	*ios xustos*	Verdigris
V, 80	ἰός σιδήρου	*ios siderou*	Iron rust
V, 81	πεπλυμένος μόλυβδος	*peplumenos molubdos*	Washed lead
V, 82	σκορία μολύβδου	*skoria molubdou*	Lead dross
V, 83	μολυβδοειδὴς λίθος	*ho molubdoeides lithos*	Leadstone
V, 84	στίβι	*stibi*	Antimony
V, 85	μολύβδαινα	*molubdaina*	Galena
V,86	ἡ τοῦ ἀργύρου σκορία	*he argurou skoria*	Silver dross
V, 87	λιθάργυρος	*litharguros*	Litharge
V, 88	ψιμύθιον	*psimmithion*	White lead
V, 89	χρυσοκόλλα	*khrusokolla*	Chrysocolla
V,90	᾽Αρμένιον	*Armenion*	Azurite
V, 91	κύανος	*kuanos*	Lapis lazuli
V, 92	Ἰνδικόν	*Indikon*	Indigo
V,93	ὦχρα	*ōkhra*	Yellow ocher
V, 94	κιννάβαρι	*kinnabari*	Cinnabar
V, 95	ὑδράργυρος	*hudrarguros*	Mercury
V, 96	μίλτος Σινωπική	*miltos sinopike*	Sinopic red earth
V, 97	Λημνία γῆ	*Lēmnia gē*	Lemnian earth
V,98	χαλκανθές	*khalkanthes*	Copper sulphate solution
V, 99	χαλκῖτις	*khalkitis*	Rock alum
V, 100	μίσυ	*misu*	Misy
V, 101	μελαντηρία	*melantēria*	Shoemaker's black
V, 102	σῶρι	*sori*	Melanterite
V, 103	διφρυγές	*diphruges*	Pyrite from copper mines
V, 104	ἀρσενικόν	*arsenikon*	Yellow orpiment
V, 105	σανδαράκη	*sandarakē*	Sulphide of arsenic
V, 106	στυπτηρία	*stuptēria*	Alum
V, 107	θεῖον	*theion*	Sulphur
V, 108	κίσηρις	*kisēris*	Pumice stone
V, 109	ἅλς	*hals*	Salt
V, 110	ἁλός ἄχνη	*halos akhnē*	Salt froth
V, 111	ἅλμη	*halmē*	Brine
V, 112	ἄνθος ἁλός	*anthos halos*	Salt efflorescence
V, 113	νίτρον	*nitron*	Soda
V, 114	τρύξ	*trux*	Wine lees
V, 115	ἄσβεστος	*asbestos*	Unslaked lime
V, 116	γύψος	*gupsos*	Gypsum
V, 117	τέφρα κληματίνη	*tephra klematinē*	Ash of vine twigs
V, 118	ἀλκυόνειον	*alkuoneion*	Alcyonion
V, 119	ἀδάρκης	*adarkēs*	Adarces
V, 120	σπόγγοι	*spongoi*	Sponges
V, 121	τὸ κουράλιον	*to kouralion*	Coral
V, 122	ἀντιπαθές	*antipathes*	Antipathes
V, 123	λίθος Φρύγιος	*lithos Phrugios*	Phrygian stone
V, 124	Ἄσσιος λίθος	*Assios lithos*	Assian stone
V, 125	πυρίτης λίθος	*purites lithos*	Copper pyrites,
V, 126	αἱματίτης λίθος	*haimatithēs lithos*	Hematite
V, 127	σχιστὸς λίθος	*skhistos lithos*	Talc
V, 128	γάγατος	*gagatos*	Lignite
V, 129	Θρακίας	*Thrakias*	Thracian stone
V, 130	μαγνίτης λίθος	*magnitēs lithos*	Magnet
V, 131	’Αραβικός λίθος	*Arabikos lithos*	Arabian stone
V, 132	γαλακτίτης	*galakitēs*	Milkstone
V, 133	μελιτίτης	*melititēs*	Honey stone
V, 134	λίθος μόροχθος	*lithos morokhthos*	Pipe clay
V, 135	λίθος ἀλαβαστρίτης	*lithos alabastritēs*	Alabaster
V, 136	θυίτης	*thuitēs*	Thyites
V, 137	’Ιουδαϊκός λίθος	*Ioudaikos lithos*	Judaic stone
V, 138	λίθος ἀμίαντος	*amiantos*	Asbestos
V, 139	λίθος σάπφειρος	*lithos sapheiros*	Lapis lazuli
V, 140	λίθος Μεμφίτης	*lithos Memphitēs*	Memphitic stone
V, 141	λίθος σεληνίτης	*lithos selenitēs*	Selenite
V, 142	λίθος ἴασπις	*lithos iaspis*	Jasper
V, 143	λίθος ὀφίτης	*lithos ophitēs*	Serpentine
V, 144	λίθοι οἱ ἐν τοῖς σπόγγοις	*lithoi hoi en tois spongois*	The stones in the sponges
V, 145	λιθοκόλλα	*lithokolla*	Stone glue
V, 146	λίθος ὀστρακίτης	*lithos ostrakitēs*	Clay stone
V, 147	σμύρις	*smuris*	Emery
V, 148	ἄμμος	*ammos*	Sand
V, 149	ἀκόνη Ναξία	*akonē Naxia*	Powder from Naxian whetstone
V, 150	λίθος γεώδης	*lithos geōdes*	Earth-like stone
V, 151	γῆ	*gē*	Earth
V, 152	Ἐρετριάδος γῆ	*eretriados gē*	Eretrian earth
V, 153	Σαμία γῆ	*Samia gē*	Samian earth
V, 154	ἐν τῇ Σαμίᾳ γῇ λίθος	*en tē samia gē lithos*	Stone found in Samian earth
V, 155	Χία γῆ	*Khia gē*	Chian earth
V, 156	Κιμωλία γῆ	*Kimolia gē*	Cimolian earth
V, 157	πνιγίτις γῆ	*pnigitis gē*	Pnigitis earth
V, 158	ὄστρακα	*ostraka*	Potsherds
V, 159	Μήλια γῆ	*Mēlia gē*	Melian earth
V, 160	ἀμπελῖτις γῆ	*ampelitis gē*	Ampelitis earth
V, 161	ἀσβόλη	*asbole*	Soot
V, 162	μέλαν	*melan*	Black ink
Ι, 72	πίσσα	*pitta*	Pitch
Ι, 73	ἄσφαλτος	*asphaltos*	Asphalt

## 4 Methodology

We begin (Section 5) by considering the geology of Cyprus, to assess which minerals may be locally available to JC. Indeed, Cyprus is frequently stated in Dioscorides as the place of origin of minerals and this can lead to the realisation that either of the authors have mis-translated or mis-interpreted a mineral and/or that there may be several different minerals that have been lumped together. However, often it is necessary to also consider the components of minerals – their elements – both in terms of anions and cations. This is because it is the cations (Al, Ar, Ca, Co, Cr, Cu, Fe, Na, Pb, Sb, Zn) that are the key to the importance of a mineral to bodily functions yet it is the anions (CO_3_, O, S, SO_4_, silicates *etc.*) that allow the bioavailability of the mineral cations. We therefore constructed tables of such data (Table 3, Table 4) to help with a consideration of potential minerals to be considered with respect to their occurrence in Cyprus. The method of naming minerals has had a very long and complex history, so much so that a more rigorous international approach was instigated in 1950 with formal international approval of names (Zipser et al., 2023b).

**Table 3:** Cations in minerals. The name of each element is followed by examples of minerals containing that element, general comments, and notes on occurrence in Cyprus.

Cations	Mineral examples	Comments	Occurrence in Cyprus
Aluminium (Al)	*Gibbsite* Al(OH)_3_, *corundum* Al_2_O_3_	Often found as hydroxides and also oxides.	There are no major minerals of aluminium occurring in Cyprus.
Antimony (Sb)	*Stibnite* Sb_2_S_3_ (Fig. 2q)	The main mineral Stibnite, a lead-grey mineral that has metallic lustre but becomes dull upon weathering, is a sulphide and is associated with the main sulphide deposits dominated by iron pyrite.	Not reported in Cyprus.
Arsenic (As)	*Arsenopyrite* FeAsS_2_, *realgar* As_4_S_4_, *orpiment* As_2_S_3_ (Fig. 2u)	This mineral is usually a minor part of complex sulphide ore deposits.	Arsenic minerals are a rare association with the main sulphide ore bodies in Cyprus.
Barium (Ba)	*Baryte* BaSO_4_ (Fig. 2k)	The main mineral baryte is a massive white dense crystalline mineral.	It does not appear to be a common mineral in Cyprus.
Cadmium (Ca)	Greekockite CdS	Cadmium is always a minor element and is usually found linked to a range of iron and copper sulphides.	No cadmium minerals have been reported from Cyprus, however many cadmium bearing minerals are found in Greece.
Calcium (Ca)	*Calcite* CaCO_3_ (Fig. 2l), *aragonite* CaCO_3_	Abundant associated with all rock types.	Widely distributed.
Cobalt (Co)	*Cobaltite* CoAsS	Cobalt is always a minor element and is usually found linked to a range of iron and copper sulphides.	Cobalt minerals have not been reported a separate mineral species but may occur in massive sulphide deposits.
Copper (Cu)	*Bornite* Cu_5_FeS_4_, *chalcocite* Cu_2_S, *chalcopyrite* CuFeS_2_, *covellite* CuS, *cuprite* Cu_2_O (Fig. 2s), *idaite* Cu_3_FeS_4_	A number of quite different minerals contain both iron and copper as sulphides. In addition, other copper minerals exist in the oxidation zones of sulphide ores or sometimes as a result of ore extraction processes.	Cyprus is famous for its copper minerals. However, the most important copper ores are a mixture of iron and copper sulphides (Fig. 2)
Chromium (Cr)	*Chromite* (Mg, Fe) Cr_2_O_4_ (Fig. 2o)	The mineral, which is black or brown in colour occurs in patches but are particularly rich in chromium.	Chromium minerals occur in Cyprus associated with the rocks hartzburgite and dunite.
Iron (Fe)	*Goethite* (brown ochre) FeO (OH), *hematite* Fe_2_O_3_ (Fig. 2r), *iron pyrite* FeS_2_ (Fig. 2a), *limonite* FeO (OH), *marcasite* FeS_2_, *magnetite* Fe_3_O_4_, *ochre* FeO (OH)·*n*H_2_O, *pyrrhotite* Fe_(1__−__x)_S, *siderite* FeCO_3_, iron oxides — *umber* iron and manganese oxide Fe_2_O_3_ + MnO_2_ + nH_2_O + Si + AlO_3_ (Fig. 2f)	Many of the iron minerals may be collected for iron alone. However there are a significant number with other elements attached. In particular the occurrence of sulphur compounds, manganese and aluminium. We should also note the copper and iron sulphides often co-occur.	These are the most common minerals in Cyprus. Most of the famous copper deposits are hosted by iron minerals, especially sulphides.
Lead (Pb)	*Galena* PbS (Fig. 2m)	Important part of many sulphide ore deposits.	Lead minerals are not particularly common in Cyprus but are occasionally associated with other sulphide deposits.
Lithium (Li)	*Spodumene* LiAlSi₂O₆ and *lepidolite* K(Li, Al, Rb)_2_(Al, Si)_4O_10__(F, OH)_2_	Lepidolite is a member of the mica group associated with granites.	Widely found as accessory minerals.
Magnesium (Mg)	*Magnesite* MgCO_3_	It is often found as an alteration product of ultramafic rocks including serpentinite.	Note recorded separately in Cyprus but likely to occur as a subsidiary mineral.
Manganese (Mn)	*Pyrolucite* MnO_2_	Such minerals occur associated with hydrothermal veins. They include a number of oxides and dioxides but are difficult to recognise.	This has not been reported in Cyprus.
Molybdenum (Mo)	*Molybdenite* MoS_2_ (Fig. 2p)	Found in high temperature hydrothermal veins.	Molybdenum minerals are not particularly common in Cyprus but are occasionally associated with other sulphide deposits.
Nickel (Ni)	*Milerite* NiS	Nickel occurs mainly associated with other metallic sulphides but although there are a large range of nickel minerals these are relatively uncommon as separate entities.	Nickel may occur as subsidiary minerals associated with other ore sulphides.
Potassium (K)	*Silvite* KCl	Mainly associated with salts rather than as a standalone mineral	See sodium occurrence.
Sodium (Na)	*Halite* NaCl	Occurs as salt and associated with seawater	Widespread occurrence.
Strontium (Sr)	*Celestine* SrCO_3_, *strontianite* SrCO_3_	Widely associated with a minor element with a range of minerals.	Occurs mainly in sedimentary rocks such as bedded salt deposits, also in bedded limestone and dolomite in cavities
Sulphur (S)	Elemental *sulphur* and in sulphides	Widely found, mainly as sulphide ore minerals.	Widespread occurrence.
Titanium (Ti)	*Ilmenite* FeTiO_3_	Widely associate with a range of igneous deposits, especially basic and ultrabasic forms and also as ‘black sand’.	Titanium minerals are not particularly common in Cyprus but are occasionally associated with the oxidisation zones of sulphide deposits.
Zinc (Zn)	*Sphalerite* ZnS (Fig. 2r)	Often associated with iron and lead sulphide deposits	Zinc minerals are not particularly common in Cyprus but are occasionally associated with other sulphide deposits.

**Table 4:** The anions in minerals. The name of each element is followed by examples of minerals containing that element, general comments, and notes on occurrence in Cyprus.

Anions	Mineral examples	Comments	Occurrence in Cyprus
Carbonates (CO_3_)	*Aragonite*, *calcite*, *siderite*, *azurite* (very rare in Cyprus), *malachite* (may not occur in Cyprus).	These minerals may be easily ground to fine powders and hence may be more able to release their metallic cations by simple mixing with some acids but also internally after suspension in a liquid.	Carbonate minerals are very common in Cyprus, especially calcites and aragonites. Although siderite is common copper carbonates appear to be relatively rare.
Oxides (O)	*Chromite*, *cuprite*, *goethite* (brown ochre), *hematite*, *ilmenite*, *limonite*, *ochre*, *magnetite*, *umber* (Fig. 2f).	The weathering of the metalliferous sulphide deposits and also their interaction with hot fluids give rise to a large number of oxide minerals.	A large number of oxide minerals such as large deposits of umber as well as goethite, limonite and ochre.
Sulphides (S)	*Arsenopyrite*, *bornite*, *chalcocite*, *chalcopyrite*, *covellite*, *cuprite*, *galena*, *idaite*, *marcasite*, *mackinawite* (Fe,Ni)_9_S_8_, *molybdenite*, *orpiment*, *pyrrhotite*, *realgar*, *sphalerite*, *stibnite*.	Such minerals are both naturally oxidised by weathering as well as reacting to acids such as wine vinegar (see Section 6).	Sulphides are probably the most common minerals in Cyprus. In addition they form the basis of the major industrial processes and products and are the most likely minerals to have been considered for any medicinal use.
Sulphates (SO_4_)	*Anhydrite* CaSO_4_, *baryte*, *epsomite* (MgSO_4_·7H_2_O), *gypsum* CaSO_4_·2H_2_O	Rarer minerals include: Magnesium sulphates (epsomite, hexahydrite, pentahydrite and starkeyite); magnesium–aluminium sulphates (pickeringite); copper sulphates (chalcanthite, langite, antlerite); iron sulphates (melanterite, copiapite, halotrichite, siderotil, fibroferrite, jarosite, bilinite); calcium sulphates (gypsum); sodium sulphate (thenardite); sodium–magnesium sulphates (bloedite, conyaite, loeweite); sodium–calcium sulphates (glauberite, wattevilleite) and aluminium sulphates (alunogen).	Sulphates often occur as weathering products of metal orebodies, especially where weathering reactions create acidic water. Many of these minerals are brightly coloured and may have a form that is reminiscent of other less reactive minerals such as some silicate and some carbonate minerals. These minerals may be reactive even in water and certainly in acidic liquids such as wine vinegar (see Section 6)
Salts (Cl)	*Halite* (salt) NaCl	The variety of chemical compositions of such salty waters makes identification of which are intended for use in recipes to be difficult.	Salts are readily abundant in Cyprus both as solid salts from geological deposits through to those found in evaporating lakes.
Silicates (Si)	Serpentine and asbestos variety (*Chrysotile* Mg_6_Si_4_O_10_ (OH))	The most problematic minerals to identify in ancient texts are silicate minerals. *Celadonite* (Fig. 2l): mica group mineral, a phyllosilicate of potassium, iron in both oxidation states, aluminium and hydroxide with formula K(Mg,Fe^2+^)(Fe^3+^,Al)[Si_4_O_10_](OH)	Ultrabasic rocks are common in Cyprus with a wide variety of silicate minerals.
Clays	(Hydrous sheet silicates): *Montmorillonite*, *smectite*, *chlorite*, *kaolinite*, *illite*, *glauconite*.	These minerals may be easily ground to fine powders and hence may be more able to release their metallic cations by simple mixing with some acids but also internally after suspension in a liquid.	A range of clays are available throughout Cyprus including those containing important types such as montmorillonite
Others: Boron (B), chlorine (Cl), iodine (I), molybdenum (Mo) (as molybdenate), phosphorus (P), selenium (Se), tellurium (Te)	Many of these occur only as trace elements in a range of minerals (*e.g.,* boron, iodine, phosphorus, selenium and tellurium). Chlorine occurs mainly as soluble chlorides.	Salts occur widely and are chlorides. Phosphorus may be found extensively in guano.	Salts are widely found and guano may be readily available. Many of these elements are associated with a range of other minerals.

An important clue to the identification of a mineral or indeed element may come from the medical condition for which it is used. To aid such an approach, in Section 6 we considered the potential impacts of each of the elements, both in terms of a cause of a condition and for a treatment of a condition. It was hoped that this may provide further clues to the use of a particular substance.

In Section 7 we integrate the results of these studies, assessing the plausibility of identifications and commenting on medical use. Section 8 describes our experiments with the properties of those ingredients for which JC recommends use in burnt form, to consider if they may have had any effect in terms of successful or at least rational treatment. We also took an experimental approach to see how easily some elements might be made bioavailable using a range of liquids commonly used in the recipes (water, wine, wine vinegar *etc.*). The experimental procedures are detailed in that section.

## 5 Minerals and their geological and geographical occurrence in Cyprus

### 5.1 Introduction

**Fig. 1:**
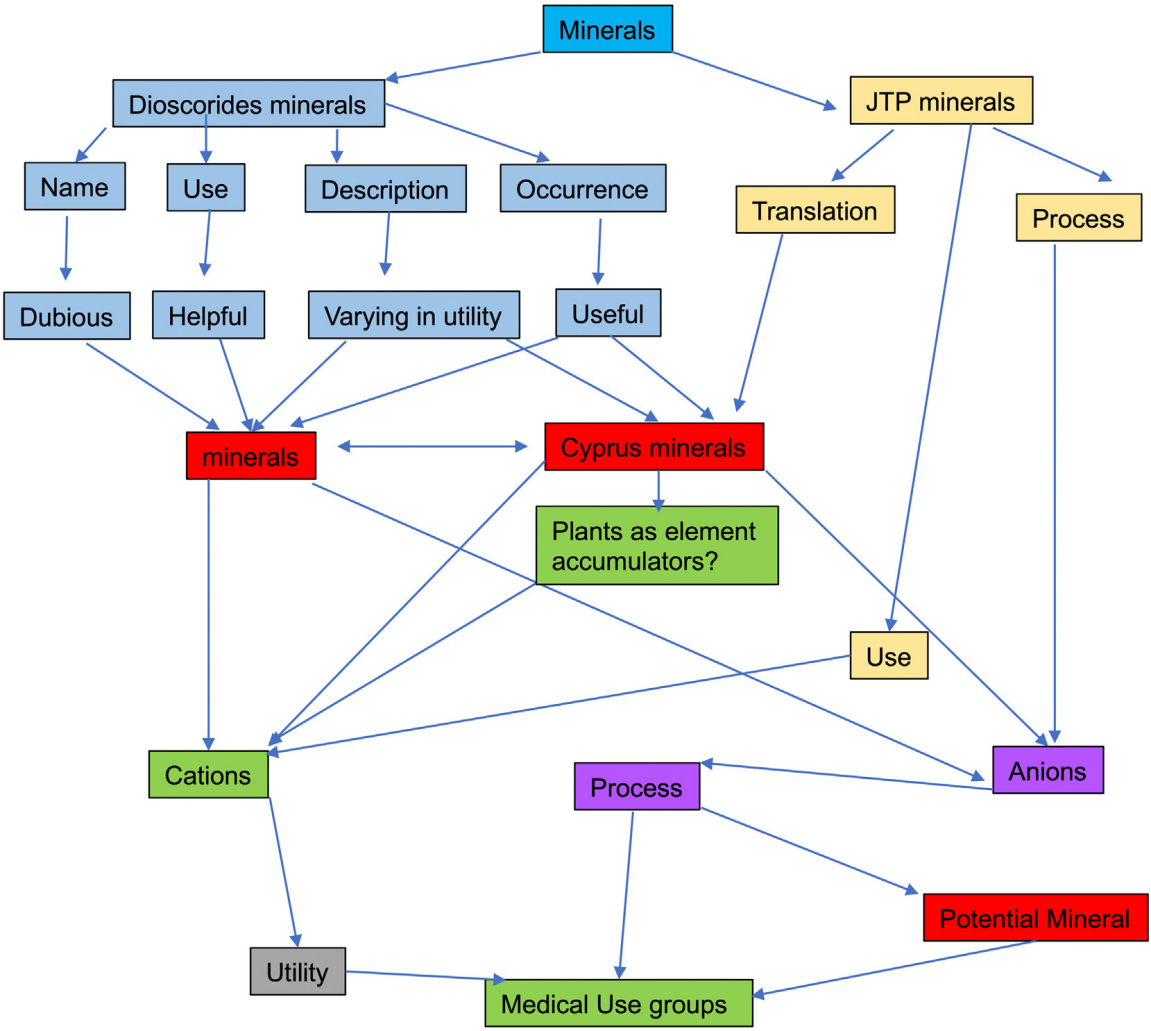
Conceptualising the identification of minerals in ancient texts.

**Fig. 2:**
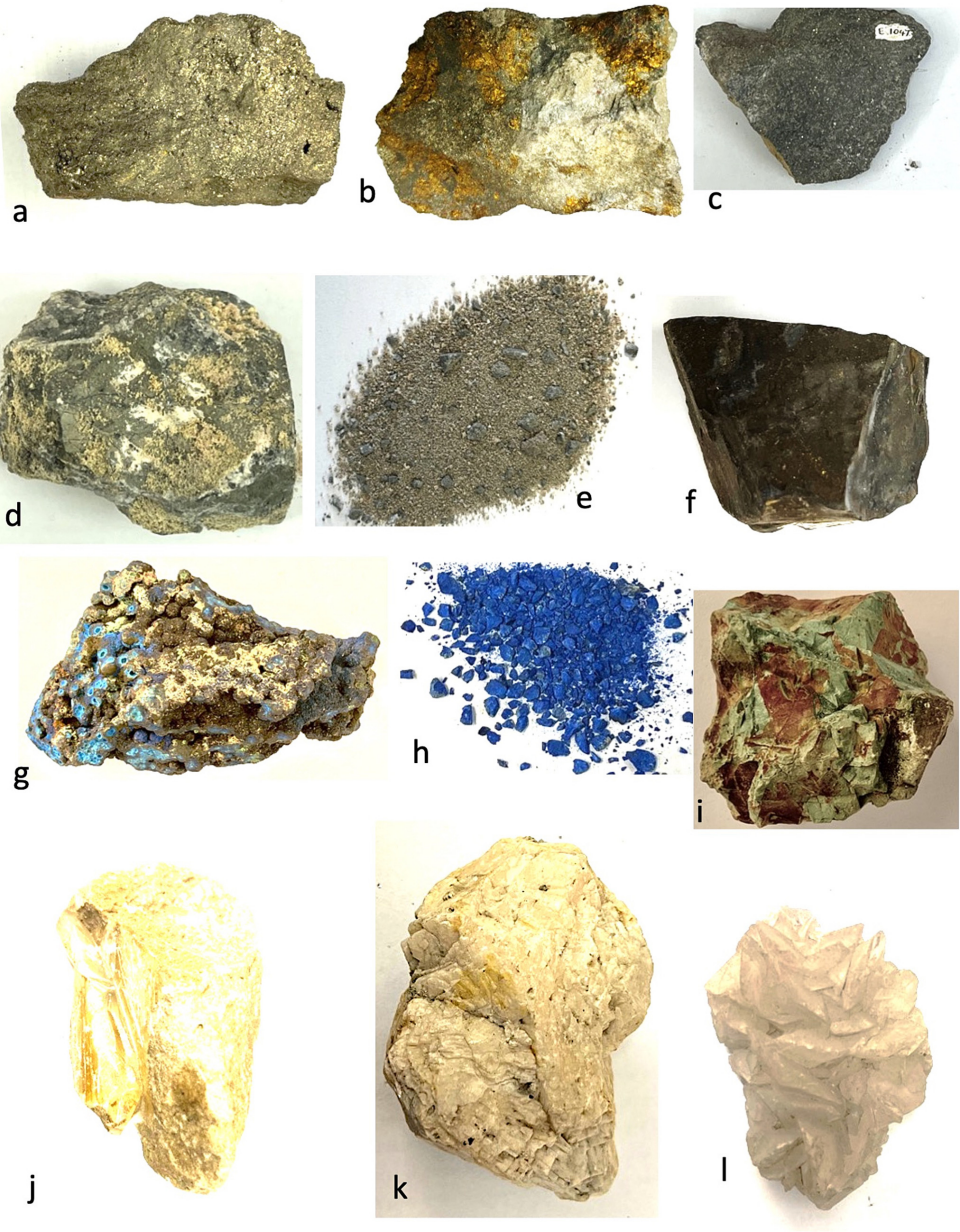

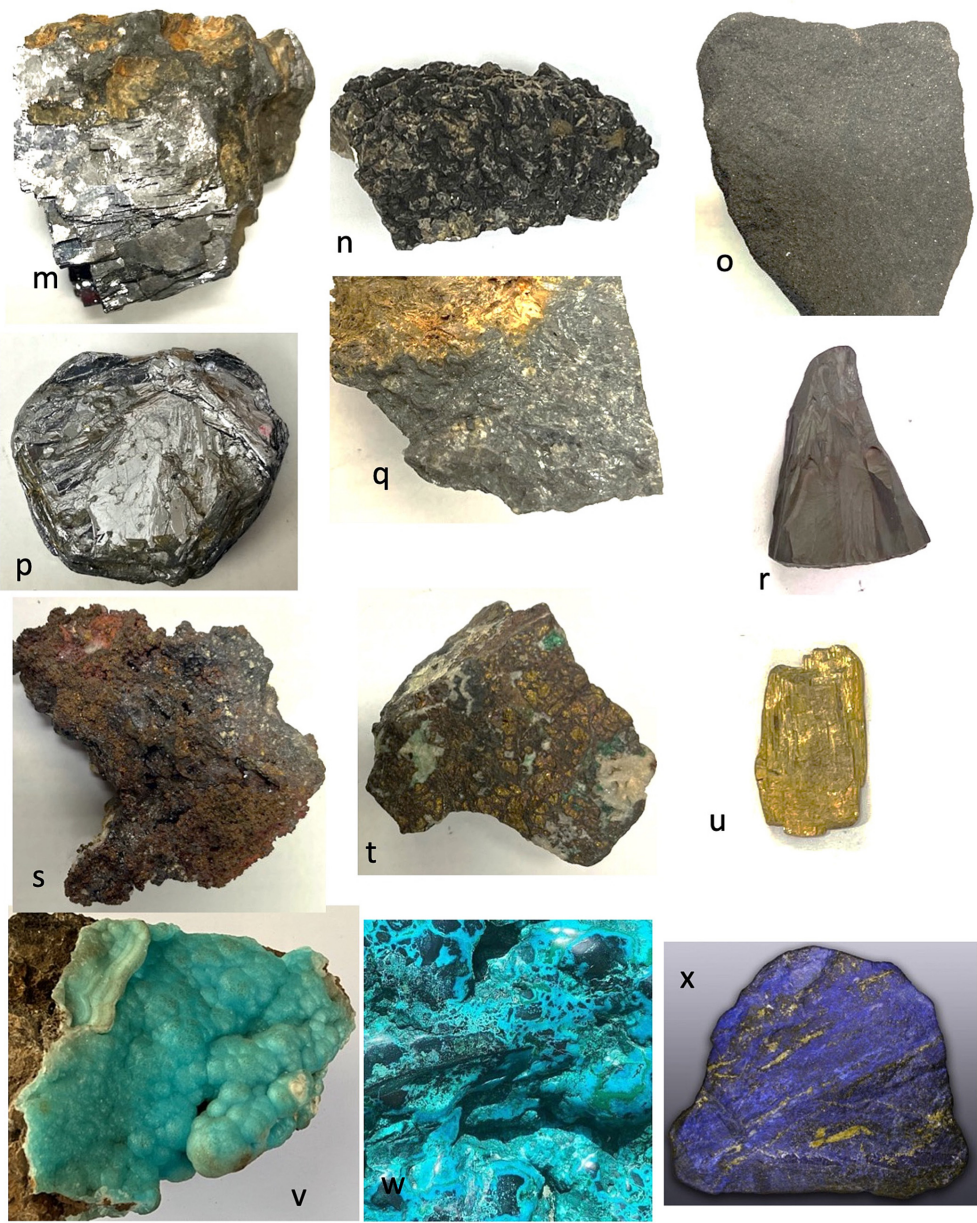
Cyprus and other comparative minerals. a. Cyprus sock sulphide containing iron pyrite and chalcopyrite, Cyprus. b. Chalcopyrite from within stock pyrite ore. Cyprus. c. Sulphide ore, Cyprus. d. Cyprus sulphide ore undergoing decay. e. Decay products from e. f. Umber from Cyprus. g. Azurite and malachite. h. Crushed azurite. i. Celadonite in pillow lavas, Klirou Bridge, Cyprus. j. Gypsum. k. Baryte. l. Calcite. m. Galena. n. Sphalerite. o. Chromite. p. Molybdenite. q. Stibnite. r. Hematite. s. Cuprite. t. Copper pyrite and malachite. u. Orpiment. v. Calamine (hemimorphite). Zinc silicate Cumberland, England. w. Chrysocolla, locality unknown. x. Lapis lazuli, Afghanistan.

Unlike plants, where a detailed description and/or illustration may provide an important clue to identification (Lardos et al., 2024), the colour and gross form of a mineral may not provide sufficient detail to identify mineral species (Fig. 1). We have previously examined three of the minerals and rocks commonly identified in Dioscorides (Zipser et al., 2023b), chrysocolla (Dioscorides V, 89 χρυσοκόλλα, kh*rusokolla*) (Fig. 2w), lapis lazuli (Dioscorides V, 91 κυανός, k*uanos*) (Fig. 2x) and calamine (Dioscorides: V, 74 καδμεία, k*admeia*) (Fig. 2v) (Table 2) (Zipser et al., 2023b). The most important clue for the misidentification of minerals came from the use of the mineral name chrysocolla. The name was derived from the Greek kh*rusos* meaning gold and k*olka* meaning glue. However, in Dioscorides (Beck, 2005) it is stated that the third best material comes from Cyprus. However, the mineral today termed chrysocolla (Fig. 2w) is unknown in Cyprus and it is clear that the simple use of the Greek name in the ancient text does not indicate an identification of a mineral given the same name by modern science. Identification of a mineral from ancient texts needs, therefore, more information such as occurrence and use.

Both the modern geology of Cyprus and its ancient exploitation by humans are well-documented (Cullis, 1924; Greensmith, 1994; Edwards et al., 2010). We should consider both minerals that are naturally found (Constantinou and Govett, 1973) and those that have formed as a biproduct of human activity such as smelting (Brempt and Kassianidou, 2016; Antivachis et al., 2017).

We know that in Cyprus the main iron and copper ores are the Volcanogenic Massive Sulphide (VMS) deposits (Fig. 2a, c) that have been worked for over 5000 years (Davies, 1930; Knapp et al., 2001). We can learn much also from an examination of ancient slags to understand the nature and uses of these deposits (Brempt and Kassianidou, 2016). The main ore is iron sulphide (iron pyrite, Fig. 2a) with copper sulphide being a minor element (Fig. 2b) (Constantinou and Govett, 1973). Whereas Cyprus is justifiably famous for its copper there is often a misunderstanding among non-geologists of how this occurs in Cyprus, and indeed the wide range of copper minerals that exist (see Greensmith, 1994; Edwards et al., 2010). These may be considered either as primary or secondary ores (sulphide ores or oxidation products such as carbonates, sulphates or oxides) or else as different chemical associations (*e.g.,* copper sulphides, copper carbonates, copper silicates) (Fig. 2). Each group may react in different ways to, for example, acids such as vinegar so that the choice of solvents is critical.

### 5.2 Geology and mineral resources of Cyprus

The Troodos massif (Cleintuar et al., 1977) is a key element in the geology of Cyprus. It is the type Ophiolite complex: that is, a piece of ocean floor with associated mineralisation (Edwards et al., 2010). The mineralisation is often hosted in a series of basic and ultrabasic rocks, some of which have undergone significant alteration such as serpentinisation. This in turn may lead to the formation of minerals such as asbestos found in serpentinised hartzburgite (Edwards et al., 2010). The major minerals are ore minerals of iron, copper, chromium, cobalt, lead and zinc, most often occurring as sulphides (Eliopoulos et al., 2020) (Fig. 2). In addition, contacts between the ore bodies and serpentine rocks yield sulphides with high concentrations of zinc, selenium, molybdenum, mercury and antimony. The minerals include not only pyrite and chalcopyrite (Fig. 2b) but also bornite–covellite, siegentite, sphalerite, selenide-clausthatite, telluride-melonite and tennantile–tetrahedrite. In addition, the sulphides pyrrhotite, chalcopyrite, bornite and sphalerite (Fig. 2n) associated with magnetite have high Cu/Cu + Ni and Pt/Pt + Pd and low Ni/Co ratios (Eliopoulos et al., 2020). Mineralogical processes are dynamic and over time some mineral phases may be altered by a range of processes, leading to an even greater variety of minerals with a wide range of chemical compositions that include not only major elements but also minor and trace elements.

The complex nature of the mineralisation also means that alteration of minerals by hydrothermal fluids is commonplace (Edwards et al., 2010). Many of the main ore minerals occur within VMS deposits. They are hosted in pre-mineralisation volcanic rocks. They occur as massive hydrothermal feeder zones, known as stockworks with the main massive sulphides occurring at the top of the units. These are then often overlain by post-mineralisation volcanic rocks. At the junction of the VMS deposits and the overlying volcanic rocks there is an oxidation zone that produces oxides such as umber (Fig. 2f) and ochre (Edwards et al., 2010).

Within some of these umber oxidation layers there are deposits of gold (Edwards et al., 2010). Greensmith (1994) also records silver associated with gold. Within the lower pillow lava layers veins of gypsum may be found (Antivachis, 2015). A number of different minerals are found in the unmineralised lavas, hydrothermally altered lavas and as sulphides in the stockwork veins. These include magnetite, leucoxene, montmorillonite, illite and kaolinite, chlorite, quartz, pyrite (Fig. 2a, c), chalcopyrite (Fig. 2a) and sphalerite (Fig. 2m) inclusions in pyrite as well as interstitial chalcopyrite and important clay minerals (Constantinou and Govett, 1973).

Many minerals may be found within these complex rock systems but simple identification of many may be difficult. The most important minerals are massive sulphides dominated by iron pyrite, but which may contain some copper (Constantinou and Govett, 1973) (Fig. 2). The pyrite may be very variable and, in some places, may be coarsely crystalline, yellow and good crystal shapes but more often is finely crystalline with colloform textures. Although minor parts both sphalerite (zinc sulphide) and chalcopyrite (iron copper sulphide) also occur (Fig. 2).

The oxidation of some of these minerals gave rise to umber (iron and manganese oxide, Fig. 2f) which may also be altered to form a silicate mineral known as jasper. Within some of the lavas there are patches of the white zeolite analcite as well as the glassy zeolite heulandite (Greensmith, 1994).

The naming of ore rocks and their component minerals is complex. For example, in some deposits the rocks are dominated by hartzburgite, chromitite and dunite with pyroxenite veins. The dominant mineral within chromitite is chromite (Fig. 2o) that is an iron chromium oxide. Dunite on the other hand may comprise 90% of the mineral olivine (a distinctive green crystal) that is a magnesium silicate mineral. In contrast harzburgite comprises the minerals olivine and low-calcium pyroxene known as enstatite.

The key minerals, both primary and secondary associated commonly with the ore bodies include pyrite, marcasite, and chalcopyrite (Antivachis, 2015) with bornite, sphalerite, galena (Fig. 2m) and baryte (Fig. 2k) being accessories with quartz in the main gangue. Goethite, hematite (Fig. 2f), chalcocite, covellite and Fe-, Cu-, Pb-, Al- and Ca-sulphates were formed in the supergene environments (Antivachis, 2015). Many of the ores have been found to contain significant quantities of the elements Ag, As, Au, Cd, Co, Fe, Ni, Pb, S, Se, Sb and Zn.

It has also been shown (Antivachis et al., 2017) that the underlying geology may lead to distinctive assemblages of minerals. For example, in the Lower Pillow Lavas secondary minerals such as celadonite, calcite and analcime may occur. In areas near the base of the main ore deposits that have been brecciated and exposed to hydrothermal waters many different clay minerals occur. The minerals include chlorite, clay minerals (smectites, kaolinite–smectites, mixed smectites, chorit–smectites), albite, quartz and some sulphides. In the main ores themselves the sulphides may be massive, and their iron and copper contents vary but the minerals are predominantly iron pyrite, marcasite and chalcopyrite. Bornite, sphalerite, galena and baryte are accessories. Other minerals were formed in the supergene environment such as goethite, hematite, chalcocite, covellite, as well as iron, copper, lead, aluminium and calcium sulphates. Veins of gypsum may be found in some of the host rocks (Antivachis et al., 2017).

In summary, therefore, associated minerals with the main ore bodies include pyrite as well as marcasite, chalcopyrite, more rarely sphalerite, pyrrhotite, covellite, chalcocite, bornite, diagenite, idolite, mackinawite as well as quartz and red jasper (Constantinou and Govett, 1973).

There is a significant thickness of Cretaceous and later sedimentary rocks that include chalks, limestones (calcium carbonate), marls (calcareous clays) and gypsum (calcium sulphate) (Fig. 2j) (Edwards et al., 2010). In addition, volcanic clays such as bentonites occur. The distinctive nature of some clay types may be a significant clue to their use, but identification of which clays or earth is described is difficult.

Salt layers and lagoons are represented by significant fossil evaporite deposits (Greensmith, 1994) as well as salt lakes that occur near the coast (Edwards et al., 2010). In Cyprus, these include the Akrotiri salt lake and the Larnaca salt lakes that contain a wide variety of salts (Greensmith, 1994).

### 5.3 Mineral extraction in Cyprus

A feature of the minerals of Cyprus is that there are a wide range of primary minerals, such as sulphide ores of iron and copper but also a range of secondary minerals such as many carbonate, sulphate or oxides resulting either from natural weathering or other geological processes or from the industrial development of the minerals, including from processes for copper smelting. Copper smelting has been reported in Cyprus from the late Bronze age (1600–1200 BCE) (Knapp et al., 2001). Most of the material obtained from excavations is a mixture of copper and iron sulphides that appear to have undergone a two-stage refinement. Copper slags have been recovered from some excavations (Brempt and Kassianidou, 2016) and it is possible that some of these rocks could have been used in John's recipes. The mineral phases found in excavations tend to be mainly iron pyrites together with chalcopyrite and chalcocite with the more yellowish material being iron-rich and the darker yellow phases being copper-rich (Fig. 2) (Brempt and Kassianidou, 2016). Some of the slags are also sulphur-rich.

Working of the copper ores and indeed exposure of a range of sulphide minerals may allow oxidation and the formation of acid waters that then also interact to form new minerals (Antivachis et al., 2017). Pyrite and chalcopyrite are so abundant that many places may yield these secondary minerals. The secondary minerals from the main ore zones are mainly iron, copper and magnesium sulphates. In contrast those secondary minerals deposited on the margins of water bodies are dominated by magnesium, calcium, sodium, and aluminium-bearing sulphate minerals (Antivachis et al., 2017). There are, however, also secondary minerals containing arsenic, copper, lead and zinc in some areas.

These secondary sulphates include magnesium sulphates (epsomite, hexahydrite, pentahydrite and starkeyite); magnesium–aluminium sulphates (pickeringite); copper sulphates (chalcanthite, langite, antlerite); iron sulphates (melanterite, copiapite, halotrichite, siderotil, fibroferrite, jarosite, bilinite); calcium sulphates (gypsum); sodium sulphate (thenardite); sodium–magnesium sulphates (bloedite, conyaite, loeweite); sodium–calcium sulphates (glauberite, watevillite) and aluminium sulphates (alunogen) (Antivachis et al., 2017).

Historical records show that copper and copper compounds (see Lev, 2010) had been used medicinally at least as early as 400 BCE (Mason, 1979). Many copper compounds were used to treat a variety of diseases during the nineteenth century, and the presence of copper in plants and animals was recognised more than 150 years ago (Ernst, 2006). For quite some time it has been widely accepted that copper is an essential trace element required for survival by all organisms, from bacterial cells to humans (Linder and Hazegh-Azam, 1996).

### 5.4 Minerals and plants

There may be a significant linkage between the geology of an area and the plants that are selected for a range of recipes. Even if the same plant is identified from several different regions, it may vary in effectiveness for a selected treatment or recipe because of the kind of soil in which the plant is growing and this may be linked to the underlying geology. It has been appreciated for some time that there are links between the biochemistry of plants, their tolerance and uptake of a range of elements and toxicity (Farago, 1994). It was discovered in the 1960s and 1970s that some plants preferentially took up certain elements such as cobalt and copper when growing in soils above ore bodies. This led to the idea of using plant chemistry as a mineral exploration tool (Cole, 1980). It has been demonstrated that a number of both metal and trace elements are essential to plant growth: These are Fe, Cu, Mn, Zn, Co, Mo, Ni, V, Na and Rb. In addition, it has been suggested that some elements that exist in low contents in plants may, at higher concentrations, become toxic to humans that consume them (*e.g.,* As, Ag, Cd and Pb) (Mehra and Fargo, 1994).

It is quite possible that the processes used in some recipes release these different elements and make them bioavailable. This may be through a range of processes including burning. What is of particular interest with regard to the plants living in a range of soils in Cyprus is that they may provide an abundance of heavy metals such as copper, cobalt, nickel and zinc in the weathered soils both of ore bodies and mining slag deposits, and that some plants may be able to hyperaccumulate these heavy metals (Brooks, 1994). Whilst we have some knowledge of which plants may act as hyperaccumulators we lack detailed studies relating to Cyprus despite the long history of copper mining on the island. It is clear that the hyperaccumulation of copper and cobalt goes together. Certain conditions considered in JC's text may be a result of copper and cobalt toxicity (see Section 6.1.3).

## 6 The significance of elements in medicine

#### 6.1.1 Introduction

When assessing the importance of a mineral in a medical context, different aspects must be considered. First, it is important to realise that the use of a mineral in any medical treatment may be because of several factors (Skinner and Berger, 2003; Hasan et al., 2013), often starting with the influence of colour. Indeed, colour may originate from different causes, from changes in crystal structure, to the incorporation of minor or trace elements into the crystal structure. It is rarely an individual mineral that is significant, but rather the elements that make up the mineral. These may affect bioavailability for external or internal use (Finkelman et al., 2001; Selinus, 2005; Hasan et al., 2013).

We can categorise minerals in terms of either cations (Table 3) or anions (Table 4); this affects the degree to which a mineral may react with a range of solutions to make the elements bioavailable. Cations have a positive charge and are mainly metallic atoms whereas anions are non-metallic atoms and have a negative charge. For example, the mineral calcite is a calcium carbonate mineral with the main metallic species being calcium. Calcium is an important element for the working of the body (Selinus, 2005). This is also the case with copper (Linder and Hazegh-Azam, 1996). However, copper may be connected to a range of anions that may increase bioavailability to the body (Selinus, 2005; Ernst, 2006). We have copper oxide (cuprite), copper carbonate (azurite, malachite), and copper sulphide (*e.g.,* chalcocite), each of which may react differently in pharmacological recipes (Dowlet and Sorenson, 1985). Equally, although the colour blue is most often associated with copper minerals and derivatives (*e.g.,* copper sulphate or blue vitriol) there are many blue minerals lacking copper (Baur, 1974).

One key piece of evidence is the availability of a mineral for the medical practitioner. This may be complicated in the case of valuable commodities that are sought for other purposes (Davies, 1930).

The second issue is whether the mineral can be transformed in a particular recipe. In this regard we need to consider the reactivity of the anions. For example, silicate minerals are difficult to dissolve whereas carbonate, sulphate and sulphide minerals may react with an acid, such as the acetic acid in vinegar (see Section 7).

Minerals may be found either as primary or secondary ores (sulphide ores or oxidation products such as carbonates or oxides). They may also represent different chemical associations (*e.g.,* copper sulphides, copper carbonates, copper silicates) (Davies, 1930; Antivachis, 2015; Antivachis et al., 2017). Each group may react in different ways to a range of liquids. We know that in Cyprus the main iron and copper ores are the massive sulphide deposits (Eliopoulos et al., 2020). We can learn much from an examination of ancient slags to understand the nature and uses of these deposits (Brempt and Kassianidou, 2016). The main ore is iron sulphide (iron pyrite) with copper sulphide being a minor element (Davies, 1930; Antivachis, 2015; Antivachis et al., 2017). Bornite also occurs (Eliopoulos et al., 2020) but is much rarer.

Another issue relates to minor or trace elements that are present within a mineral when it may be that the rarer component is the one of interest. For example, we now know that selenium is an important metallic element essential for some bodily functions (Selinus, 2005). The element rarely forms a mineral on its own as native selenium. The element is found in several mineral species, including over 100 copper minerals.

This then leads us to another possible approach, that is, a consideration of the use of a mineral in an ancient recipe book for an identified condition and then the opposite — the use of an element for the treatment or identification of a condition in modern medicine. There has been rapid development of our understanding of the use of minerals in modern medical practice. A useful example is that of Borkow and Gabbay (2009), which shows that copper has potent biocidal (including anti-fungal) properties.

#### 6.1.2 Ancient use

There has been a significant increase in interest in ancient medicines in relation to modern cancer treatments. Many of the historic approaches to cancer treatments have been summarised by Hajdu (2016) and these will be compared to treatments suggested in our texts and their current and potential use in modern medicines.

#### 6.1.3 Modern use

One aspect of ancient copper mining in Cyprus deposits (Brempt and Kassianidou, 2016) is the concentration of toxic heavy metals in soils, and in wild and cultivated plant species. Many instances have serious health implications (Christou et al., 2017). Soil heaps derived from ancient copper workings have very high concentrations of sulphur, zinc, copper and lead. Manganese concentrations are also raised. In addition, cadmium appears to be in higher concentration in figs, peanuts and lemons as well as in grains and barley straw. Eating such materials may lead to illness. Studies have also shown that heavy metal concentrations are high in native plant species such as *Inula viscosa* and *Allium ampeloprasum* (Christou et al., 2017). Iron species have a range of both positive and negative effects on human health and this often depends on the structure of the iron compound concerned.

Waters draining from old mine areas may contain a range of dissolved minerals and salts (Antivachis et al., 2017). In addition to primary dissolved iron and copper minerals, there are many secondary minerals such as magnesium-, calcium-, sodium- and aluminium-sulphate minerals and highly soluble iron sulphate salts.

### 6.2 Elements

Much discussion has taken place on the essentiality of elements, particularly trace elements, in bodily functions. The current definition (Mertz, 1998) states: *An element is considered essential to an organism when reduction of its exposure below a certain limit results consistently in a reduction in a physiologically important function, or when the element is an integral part of an organic structure performing a vital function in the organism*. For most elements, ranges of safe and adequate intakes have been defined (Lindh, 2005a). Eleven elements are consistently abundant in biological systems: hydrogen, oxygen, carbon, nitrogen, sodium, potassium, calcium, magnesium, phosphorus, sulphur, and chlorine whereas other elements may be regarded as trace elements but their role is more difficult to assess. These include lithium, vanadium, chromium, manganese, iron, cobalt, nickel, copper, zinc, tungsten, molybdenum silicon, selenium, fluorine, iodine, arsenic, bromine, and tin (Lindh, 2005b). The paramount function is to be necessary for the structure and function of significant biomolecules, mainly enzymes. The utilisation of an element in biology is intimately dependent on its uptake into the living organism (Lindh, 2005a).

Trace elements, that might be ingested as part of a diet may also have a significant effect upon bodily functions (Lindh, 2005a, Lindh, 2005b). For example, to function properly, organisms must actively pump sodium and chloride out of cells and actively take in potassium. Circuits maintained by K^+^, Na^+^, and Cl^−^ generally control the following properties in all cells of all organisms: osmotic pressure; membrane potentials; condensation of polyelectrolytes, and the required ionic strength for activity (Lindh, 2005b).

There are also close connections between magnesium and phosphate, which are involved in hormonal regulation. Magnesium is important in plants through its occurrence in chlorophyll, where it links with phosphorus through the molecule adenosine triphosphate (ATP) that provides energy for living cells. Further details of the function of the elements can be found in Selinus (2005).

Foods contain essential nutrients because of the capacity of plants, and in some cases food animals, to synthesise and/or store them. The human body, therefore, consists of substantial amounts of “mineral elements” obtained mostly from such foods (Combs, 2005). Nutritionally important mineral elements include some (*e.g.*, manganese [Mn]) that occur predominately in silicates, some (*e.g.*, zinc [Zn], selenium [Se]) that occur in silicates and sulphides, some (*e.g.*, copper [Cu], molybdenum [Mo]) that occur in sulphides or as native elements with iron (Fe), and some (*e.g.*, iron) that occur in silicates and sulphides. The most abundant of these is iron (Combs, 2005). Many of these elements are important for optimal health (Table 2 in Combs, 2005).

To better capture both ancient (Lev, 2010; Limpitlaw, 2010) and modern medicinal usages of elements we can use the structure of medicinal use groups proposed by Staub et al. (2015) that categorises treatments for a range of internal and external practices (Table 5). These codes for these categories are presented below, for each element. This data can then be linked to known medical information concerning the importance of elements and hence be useful in helping to identify mineral species used in ancient pharmacological texts.

**Table 5:** Medicinal use groups (MUG).

	Medicinal use group JC (deduced from chapters of JB)	Details (examples from JC)	ICPC (International Classification of Primary Care), symptoms	TDGW medicinal subcategories (Cook)
AN	Andrology	Male genital organ	P) Male genital system	Genitourinary medicinal disorders (2); unspecified medicinal disorders (1)
BS	Blood, spleen	Spleen, ethno-organ “spleen”	B) Blood forming organs, blood, lymphatics, spleen	Blood system disorders
CV	Cardiovascular disorders	Dropsy/oedema, internal treatment of (internal) haemorrhoids	F) Circulatory	Circulatory system disorders
DE	Dermatology	Skin disorders, injuries, wounds, bleeding wounds, animal bites, hair, topical manifestations of symptoms linked with a venereal disease, topical treatment of (external) haemorrhoids	K) Skin	Skin/subcutaneous cellular tissue disorders; Injuries
FV	Fever	Unspecific fever, intermittent fevers	A) General and unspecific (3)	Infections/infestations (2)
GI	Gastrointestinal tract	Oesophagus, stomach, intestine	C) Digestive	Digestive system disorders (1)
GY	Gynaecology	Incl. female genital organ	O) Female genital system and breast	Unspecified medicinal disorders (1)
ID	Infectious diseases	Consumptive disease (phthisis)	A) General and unspecific (2)	Infections/infestations (1)
LG	Liver and gall bladder	Liver, gall bladder	C) Digestive	Digestive system disorders (2)
MA	Maternity	Pregnancy, birth, lactation	N) Pregnancy, childbirth, family planning	Pregnancy/birth/puerperium disorders
MC	Mental conditions	Melancholia	I) Psychological	Mental disorders
MS	Musculoskeletal	Gout, rheumatism, sciatica, spasm, fractures, strains, paralysis	G) Musculoskeletal	Muscular-skeletal system disorders
MN	Metabolic, nutritional disorders	Anorexia	L) Endocrine, metabolic, nutritional	Endocrine system disorders, metabolic system disorders, nutritional disorders
NC	Neurological conditions	Nervous system, headache, migraine, analgesic	H) Neurological	Nervous system disorders
OP	Ophthalmology	Eyes	D) Eye	Sensory system disorders (1)
OC	Oral cavity	Teeth, gingiva, aphthae	C) Digestive	Infections/Infestations (2)
OT	Otology	Ears	E) Ear	Sensory system disorders (2)
RE	Respiratory tract	Lung, bronchia; cough	J) Respiratory	Respiratory system disorders (1)
RL	Rhino-laryngology	Head, nose, throat incl. tonsillitis	J) Respiratory	Respiratory system disorders (2)
UR	Urology	Kidney, bladder, urethra	M) Urology	Genitourinary medicinal disorders (1)
XY	Residual category	Unclear symptoms or disease or such that does not fit in one of the specific use groups	A) General and unspecific (1)	Unspecified medicinal disorders (1); ill-defined symptoms; immune system disorders; poisonings
–	–	–	Q) Social problems	–

TDGW = Traditional Databases Working Group. In 1995 TDGW had established various data collection standards including also a system to categorise medicinal uses (published by Cook, 1995, in Economic Botany). The TDGW categorisation system was designed for use in field studies where information on traditional uses was collected through interviews. We found that this categorisation system is less useful for our case (historical texts and intercultural (*i.e.,* ancient-modern) comparisons). Therefore we mainly relied on the categorisation system proposed by Staub et al. (2015), with some adaptations.

JB = John's Book (learned version of John's text).

JC = John's Commentary (vernacular version of John's text, with extended recipes and a more practical focus).

Use groups are largely based on Staub et al. (2015) model 6.II.

#### 6.2.1 Aluminium (Al)

Aluminium is highly biologically active (Exley, 2016) and is non-essential and toxic (Exley, 2013). Some forms of dementia are brought on by exposure to aluminium, as are some cancers, especially breast cancer (Exley, 2016). It may occur in drinking water (Solfrizzi et al., 2006). Aluminium is not used in many medical treatments. It has been used topically in the treatment of hyperhidrosis (excessive sweating) (Sanajou et al., 2021). MUG (medicinal use groups see Table 5): DE and OT.

#### 6.2.2 Antimony (Sb)

Antimony is a silvery-white metal that is found in the Earth's crust. In the environment, the antimony metal combines with other substances to form antimony compounds that can be found in ores (Gad, 2014). Antimony can have beneficial effects. It has been used as a medicine to treat people infected with certain types of parasites. Some side-effects have been reported, including heart problems, nausea and vomiting, and muscle and joint pain (Sundar and Chakravarty, 2010). One method involved drinking wine that had been left standing overnight in a cup made of antimony (McCallum, 1999). This resulted in the antimony reacting with tartaric acid in the wine to form antimony tartrate, a compound that induces vomiting. Stibnite was the main ingredient in “kohl” used by ancient Egyptian women in a type of mascara (McCallum, 1999).

Antimony can be antiparasitic, particularly against protozoal infection leishmaniasis. Uptake of antimony through the skin does not make a significant contribution to systemic exposure. Exposure to lungs through inhalation is damaging, leading to pneumonitis. It can be genotoxic (Boreiko and Rossman, 2020). Urinary antimony was found to be linked to sleep disorders (Scinicariello et al., 2017), schistosomiasis and leishmaniasis. Side-effects include cardiotoxicity, pancreatitis, respiratory irritation, abdominal pain, diarrhoea, vomiting and ulcers; an increased incidence of spontaneous abortions and disturbances in menstruation was reported in women working at an antimony metallurgical plant compared to a control group (Sundar and Chakravarty, 2010). At termination, one highest dose male had a cirrhotic liver, and three highest dose males exhibited gross haematuria (Poon et al., 1998). MUG: RE, GI, MA, LG, UR, and XY.

#### 6.2.3 Arsenic (As)

Used as a beauty product for women, for hair and to make lips and cheeks redder. Withdrawal symptoms: The symptoms of withdrawal include anxiety, indigestion, loss of appetite, vomiting, constipation and spasmodic pain. Arsenic has been associated with a variety of complications in body organ systems: integumentary, nervous, respiratory, cardiovascular, haematopoietic, immune, endocrine, hepatic, renal, reproductive system and development (Kosnett, 2013). Dermal system (skin lesions), cardiovascular (blackfoot disease), renal (proximal tube degeneration, papillary and cortical necrosis), nervous system (peripheral neuropathy, encephalopathy), hepatic system (hepatomegaly, cirrhosis, altered haem metabolism), endocrine system (diabetes), and haemotological system (bone marrow depression) (Mohammed et al., 2015). MUG: MN, RE, RE, DE, XY, and MA.

#### 6.2.4 Barium (Ba)

The ingestion of absorbable barium salts, *e.g.*, carbonate or chloride, produces a combination of ectopic ventricular contractions, ventricular tachycardia, skeletal muscle paralysis, salivation, diarrhoea, hypertension and finally, respiratory paralysis and ventricular fibrillation. Infusion of barium chloride causes hypokalaemia and cardiac toxicity (Roza and Berman, 1971). Hearing loss (Ohgami et al., 2015). MUG: GI, CV, MS, RE, XY, and OT.

#### 6.2.5 Boron (B)

Boron is an essential component of several minerals such as borax, kernite, ulexite and colemonite, and also is a trace element in minerals and also occurs in trace amounts in seawater. Boron is necessary for the growth and development of vascular plants (Shaaban, 2010), marine algae and algal flagellates, diatoms, and cyanobacteria (Dinca and Scorei, 2013). Boron plays an important role in osteogenesis, and its deficiency has been shown to adversely impact bone development and regeneration (Khaliq et al., 2018). Inorganic boron compounds (boric acid and borates) may exert toxic effects on reproduction (Bolt et al., 2020). Boron appears to have links with a range of health issues. Boron deficiency may also be linked with low magnesium (Shaaban, 2010) and boron supplements may be useful for those with hyperthyroidism and on brain function (Shaaban, 2010). Boron has also been linked to wound-healing (Pizzorno, 2015). MUG: MS, UR, MA, MN, and DE.

#### 6.2.6 Cadmium (Cd)

Cadmium has no known beneficial function in the human body. Cadmium is a cumulative toxin. The surplus effects can lead to skeletal damage (osteoporosis) (Jarup et al., 1998). Tubular damage, increased occurrence of kidney stones, a possible increased prevalence of osteoporosis, renal morbidity and mortality, effects on female reproductive system, necrosis of the testicles, and carcinogenic (Rzigalinski and Strobl, 2009). MUG: UR, MS, MA, GY, and AN.

#### 6.2.7 Calcium (Ca)

Calcium has an important role in the body being involved in the formation of bone and teeth, muscle contraction, normal functioning of many enzymes, blood clotting, and normal heart rhythm (Brini et al., 2013). Calcium supplementation has been found to be beneficial for bone health in children, young adults, and menopausal women. Deficiency effects: calcium once was thought to play a role in the formation of kidney stones, and patients with stones often received a low-calcium diet. More recent studies now suggest that a low-calcium diet may increase the risk of developing kidney stones. Surplus effects: Calcium supplements are generally well tolerated; however, some patients complain of gastrointestinal symptoms, including constipation, gas, flatulence, and bloating (Straub, 2007; Lewis III, 2022). MUG: MS, UR, and GI.

#### 6.2.8 Carbon (C), coal and charcoal

There are two main forms of carbon that may be important in modern and ancient medicine (Jones, 2011): coal and coal derivatives including coal tar, and charcoal.

a. Coal tar: Coal tar is a distillate of coal that only became widely available in the eighteenth century. It may not have been known in the ancient world. However some natural tars occur commonly, particularly in the Middle East. The composition of coal tar will be influenced both by the starting material and the process used in its production (Roberts, 2014). Today, coal tar is often used externally to be used on the skin and scalp to ease itching, irritation and dry parches on the skin. It also has some antibacterial properties (Paghdal and Schwartz, 2009; Ghafoor et al., 2022). Coal tar is also mixed with a range of other materials in preparations including zinc oxide and sulphur. It is known to have side-effects especially for pregnant women and may be classified as a carcinogen (Roberts, 2014). MUG: DE.
b. Charcoal: Charcoal is primarily used in the form of activated charcoal (Ajala et al., 2022). Activation is often achieved by a number of processes that may involve heating the charcoal twice at two different temperatures (*e.g.,* 300 °C, 600 °C and in some cases 900 °C; see Mallick et al., 2019 for range of alternative treatments) and adding a liquid (such as zinc chloride) that evaporates during pyrolysis (heating without oxygen) (Cherik and Louhab, 2017), or potassium hydroxide for example (Yeung et al., 2014). These treatments create minute holes in the charcoal to increase the surface area of the material and hence the adsorbent capacity of the material, used both in medicine and pollution control (Ajala et al., 2022). The ability of activated charcoal to adsorb and potentially absorb toxic metals such as copper and lead may be significant (Yeung et al., 2014). Charcoal has both external and internal uses. Charred material can be activated from a range of plant waste products including straw, wood sawdust (Castro et al., 2019), coconut husks, coffee residues (Yeung et al., 2014) and date and olive stones (Cherik and Louhab, 2017).
  Externally activated charcoal has often been used in dental medicine (Brooks et al., 2017a, Brooks et al., 2017b) as well as in the internal gastrointestinal conditions where it acts as an absorbent (Bond, 2002). Charcoal has been widely used in dental hygiene treatments from at least Roman times (Newsom, 2004) and is still used today (Brooks et al., 2017a, Brooks et al., 2017b). MUG: GI and OC.

#### 6.2.9 Chlorine (Cl)

Whilst chlorine is important to the ionic balance of cells (Selinus, 2005), helping to maintain osmotic potential and the acid/base balance of cytoplasm it is also a component of stomach acid. However chlorine may not have been widely used in Byzantine medicine, just as it is not used as a gas treatment today (Achanta and Jordt, 2021), because of its high toxicity. It is possible that the gas may be released through some recipes that include, for example salt (sodium chloride) (Fukayama et al., 1986). MUG: None.

#### 6.2.10 Chromium (Cr)

Chromium is considered an essential trace element but this has been disputed (Vincent, 2017). It occurs in many foods (Anderson and Cefalu, 2010). It has been found to help in the regulation of insulin (and blood sugar control for type 2 diabetics), but this has been disputed (Landman et al., 2014). Many other potential uses have not been scientifically proven (Vincent, 2017; Vincent and Brown, 2019). Chromium deficiency has not been reported. MUG: XY.

#### 6.2.11 Cobalt (Co)

Cobalt is an essential trace element for the human body and can occur in organic and inorganic forms (Taylor and Marks, 1978). The organic form is a necessary component of vitamin B_12_ and plays a very important role in forming amino acids and some proteins in nerve cells, and in creating neurotransmitters that are indispensable for correct functioning of an organism (Czarnek et al., 2015). It is a cytotoxic compound, and its administration under some circumstances can be harmful. Hyperaccumulation of copper and cobalt is also of considerable interest because of the significance of such accumulation for diverse fields such as phytochemistry, plant physiology and mineral exploration. Concentrations of cobalt in plants tend to reflect the composition of the soil since cobalt is not usually considered to be essential for plant nutrition (Brooks et al., 1980). The use of cobalt in the treatment of anaemia may produce a beneficial response (Taylor and Marks, 1978). It helps avoid fatality by pernicious anaemia and is involved in metabolism of iron. Surplus effects: Cobalt chloride is a recognised cytotoxic compound, and its administration can be harmful; under some circumstances a beneficial response is produced. For more than 40 years it has been known that cobalt produces polycythaemia in many animals and humans (Taylor and Marks, 1978). Cobalt has repeatedly been shown to induce hyperlipaemia. Raw cobalt chloride causes gastrointestinal symptoms, *e.g.,* nausea, vomiting and anorexia, but with present regimes of low doses given in enteric-coated capsules these effects are relatively uncommon. Peripheral neuropathy, tinnitus and auditory nerve damage develop on rare occasions (Duckham and Lee, 1976). Cobalt also interferes with thyroid function; reduced thyroidal I'31 uptake, goitre and myxoedema have all been described (Kriss et al., 1955). Can also potentially cause tumours. It can inhibit enzymes (Taylor and Marks, 1978). MUG: XX, MN, YY, and OT.

#### 6.2.12 Copper (Cu)

Because copper is an essential element for plant nutrition (Vetchý et al., 2018), the copper content of most plants tends to be internally rather than externally controlled, so that concentrations in plants are relatively constant and independent of the nature of the substrate (Timperley et al., 1970). This constancy of copper concentrations in plants sometimes breaks down when the copper content of the soil is very high. Copper is important for the body to make red blood cells as well as for the functioning of many metabolic pathways (Barceloux, 1999; Scheiber et al., 2013). To prevent infection of fresh wounds the ancient Greeks sprinkled a dry powder composed of copper oxalate and copper sulphate on the wound. Another copper-containing antiseptic wound treatment was a boiled mixture of honey and red copper oxide (Dowlet and Sorenson, 1985). The Greeks had easy access to copper since this metal was found in large amounts on the island of Kypros (Cyprus) from which the Latin name for copper *cuprum* is derived (Davies, 1930; Antivachis, 2015; Brempt and Kassianidou, 2016). It was used for venereal inflammatory conditions as well as ulcers and sore throats. Black copper oxide was given with honey to remove intestinal worms. When diluted and injected as drops into the nostrils, it cleared the head, and when taken with honey or rosewater it purged the stomach. It was given for “eye roughness and eye pain and mistiness” and ulceration of the mouth (Dowlet and Sorenson, 1985). It stopped bleeding from nostrils and haemorrhoidal bleeding. Mixed with henbane it drew out splinters. Applied to the forehead with a swab it arrested watering of eyes; used in plasters it was efficacious for cleansing wounds and ulcers (Dowlet and Sorenson, 1985). A mere touch of a decoction of it removed swellings of the uvula, and it was laid with linseed oil on plasters used for relieving pain. It was blown *via* tubes into the ears to relieve ear problems. It was also employed in ancient India and Persia to treat lung diseases (Dowlet and Sorenson, 1985). It was used for parasites such as helminths, particularly nematodes. Paracelsus made and wore “constellation rings” made of iron, lead, tin, copper, gold and silver which according to beliefs of his time would bring the wearer under the protective influence of Mars, Saturn, Mercury, Venus, the Sun or the Moon respectively. Copper compounds were also used by the ancient, medieval and nineteenth century physicians for the treatment of conditions such as 'trembling limbs'. Copper has anticonvulsant effects (Dowlet and Sorenson, 1985).

Copper plays a role in the function of the nerve system in general and a deficiency can cause anaemia (Myint et al., 2018). MUG: MN and XY.

#### 6.2.13 Iodine (I)

The importance of iodine has long been recognised in medicine, although it was not until 1811 that the actual element was identified. It was used inadvertently through its occurrence in both seaweeds and sponges. Ancient and medieval Greek doctors used sponge and seaweed as cures for swollen neck. Italian physicians of the School of Salerno were the first to report the specific use of the sponge and dried seaweed to treat goitre in the 13th century: de Villanova cautioned that the effect of the sponge on goitre was limited; it could cure goitre of recent origin in young people but had only a modest effect on large, chronic goitres (Rohner et al., 2014). Iodine has also been used as a treatment for wounds because of both its strong antibacterial properties and help in stopping blood flow (Vallin, 1882; Selvaggi et al., 2003; Vermeulen et al., 2010).

Deficiency effects: Goitre (swelling of thyroid gland). All ages: Goitre increases susceptibility of the thyroid gland to nuclear radiation. Foetuses — abortion and stillbirth, congenital anomalies, and perinatal mortality; neonates — infant mortality and endemic cretinism. Children and adolescents — impaired mental function and delayed physical development. Adults — impaired mental function, reduced work productivity, toxic nodular goitre, increased occurrence of hypothyroidism in moderate-to-severe iodine deficiency, and increased occurrence of hypothyroidism in mild-to-moderate iodine deficiency. Surplus effects: an overdose of iodine can also lead to hyperthyroidism (overactive thyroid) (Riggs, 1955; Institute of Medicine (US) Panel on Micronutrients, 2001). MUG: DE, MA, MC, and XY.

#### 6.2.14 Iron, (Fe)

Iron is one of the most common elements on Earth and is often encountered in minerals and rocks as a sulphide or oxide ore. Iron is a major component of haemoglobin, the protein in red blood cells that carries oxygen from the lungs to all parts of the body.

Deficiency: Iron is the most abundant transition metal in the brain, where it is required to sustain the brain's high respiratory activity, for myelinogenesis, and the production of several neurotransmitters including dopamine and norepinephrine. Iron deficiency is well-established to impair brain development and cognition (Elstrott et al., 2020).

Excess: Major causes of systemic iron overload are hereditary haemochromatosis, iron loading anaemias and transfusional or other secondary forms of iron overload. Several other chronic liver diseases including alcoholic liver disease, non-alcoholic fatty liver disease, and viral hepatitis, are also associated with liver iron loading. Diabetes mellitus is a common complication of iron overload disorders such as heamochromatosis. Iron overload has been associated with many neurodegenerative disorders. Iron accumulation is also associated with more common neurodegenerative diseases including Parkinson's disease and Alzheimer's disease. Iron and iron-induced reactive oxygen species have been implicated in the pathogenesis of multiple models of acute kidney injury (Dev and Babitt, 2017). The physiological manifestations of iron deficiency have also been noted in immune function, cognitive performance and behaviour, thermoregulatory performance, energy metabolism, and exercise or work performance, many of these manifestations of iron deficiency are not mutually exclusive events and do not occur independently of each other (Beard et al., 2009).

Iron deficiency anaemia (Camaschella, 2015) can also occur when there are stomach ulcers or other sources of slow, chronic bleeding (colon cancer, uterine cancer, intestinal polyps, haemorrhoids, *etc.*). Iron deficiency during pregnancy is associated with a variety of adverse outcomes for both mother and infant, including increased risk of sepsis, maternal mortality, perinatal mortality, and low birth weight (Beard et al., 2009). During the 17th century, iron was used to treat chlorosis (green disease), a condition often resulting from iron deficiency (Abbaspour et al., 2014). MUG: RE, MN, XY, UR, MA, SU, GI, MC, BS, and NC.

#### 6.2.15 Lead (Pb)

Lead is the most important toxic heavy element in the environment. There is almost no function in the human body which is not affected by lead toxicity (Dietert and Piepenbrink, 2006; Y. Wang et al., 2020). Of all the organs, the nervous system is the mostly affected target in lead toxicity, both in children and adults (Dietert and Piepenbrink, 2006.) The impact in children is greater than in adults. Lead poisoning has also been found to be the cause of anaemia in a number of cases as lead inhibits porphobilinogen synthase and ferrochelatase, preventing both porphobilinogen formation and the incorporation of iron into protoporphyrin IX, which prevents haem synthesis (Cohen et al., 1981) or causing ineffective haem synthesis and subsequently microcytic anaemia. In adults, abdominal colic, involving paroxysms of pain, may appear at blood lead contents higher than 80 μg/dL (Kosnett, 2005). High blood lead concentrations which exceed 100 μg/dL cause very severe manifestations, like signs of encephalopathy (condition characterised by brain swelling) accompanied by increased pressure within the skull, delirium, coma, seizures, and headache (Henretig, 2006). Central nervous system and neuromuscular manifestations usually result from intense exposure, whilst gastrointestinal features usually result from exposure over longer periods (Brunton et al., 2007). Signs and symptoms of chronic exposure include loss of short-term memory or concentration, depression, nausea, abdominal pain, loss of coordination, and numbness and tingling in the extremities (Patrick, 2006a, Patrick, 2006b). Fatigue, problems with sleep, headaches, stupor, slurred speech, and anaemia are also found in chronic lead poisoning (Pearce, 2007). Children with chronic poisoning generally show aggressive behaviour and refuse to play (Wani et al., 2015). Prenatal exposure to lead has been correlated with antisocial behaviour and schizophrenia. Long-term lead exposure causing low and medium lead concentration in blood has been linked to depression as well as generalised anxiety disorder and other behavioural disorders. High blood concentrations correlate with psychotic symptoms like delusions and hallucinations but more rarely with psychotic syndromes. In a prospective study conducted in Cincinnati, prenatal and average childhood blood lead concentrations were reported to be associated with a greater risk of delinquent behaviour later in life (Vorvolakos et al., 2016). MUG: NC, MC, and UR.

#### 6.2.16 Lithium (Li)

Lithium occurs as a major element in some minerals, such as spodumene and lepidolite, but also as a trace element in other minerals. Its role in bodily functions has only been recognised recently and its exact mode of action is uncertain (Shorter, 2009). In the nineteenth century, lithium was used in people who had gout, epilepsy and cancer (Sneader, 2005). Lithium is used primarily for long-term (“prophylactic”) treatment of bipolar disorder with the aim to prevent further manic and depressive recurrences (Bauer et al., 2014). The first modern use of lithium was for treatment of mania. Lithium has also proven useful in major depression, particularly for augmentation of antidepressants; for aggressive behaviour, it has a specific anti-suicide effect. The emerging picture stresses effects in multiple nodes of regulatory networks, in which lithium may dampen excessive activity and thus contribute to stabilisation of neuronal activity, stress resilience, improved neuronal/synaptic plasticity and regulation of chronobiological processes (Alda, 2015). MUC: MN.

#### 6.2.17 Magnesium (Mg)

Magnesium sulphate (Epsom salts) has been used to treat ailments, such as constipation, insomnia, and fibromyalgia for hundreds of years (Ingraham, 2016). Its effects on these conditions are not well researched (de Baaij et al., 2015). The salt is an important constituent of evaporitic salt deposits. The main external use is as bath salts and to soothe sore feet, claimed to also soothe and hasten recovery from muscle pain, soreness, or injury (Fawcett et al., 1999; van der Plas et al., 2013). Potential health effects of magnesium sulphate are reflected in medical studies on the impact of magnesium on resistant depression (Eby III and Eby, 2010) and as an analgesic for migraine and chronic pain (de Baaij et al., 2015). Magnesium sulphate has been studied in the treatment of asthma, preeclampsia, and eclampsia (Euser and Cipolla, 2009). Internal uses include replacement therapy for magnesium deficiency and for the treatment of severe arrhythmias (Guerrera et al., 2009; Gröber et al., 2015; Fiorentini et al., 2021). It also may be used as a laxative (Izzo et al., 1996). Magnesium plays a number of critical roles in the body and is a co-factor in more than 300 metabolic reactions (Volpe, 2013). Low concentrations of magnesium have been associated with chronic diseases including migraine headaches, Alzheimer's disease, cerebrovascular accident (stroke), hypertension, cardiovascular disease, and type 2 diabetes mellitus (Volpe, 2013). Symptoms of magnesium deficiency include: loss of appetite, nausea or vomiting, fatigue or weakness. Symptoms of more advanced magnesium deficiency include: muscle cramps, numbness, tingling, seizures, personality changes, heart rhythm changes or spasms. Good food sources of magnesium include unrefined (whole) grains, spinach, nuts and legumes. It has been reported that magnesium may be an effective complementary treatment for migraine headaches (Guerrera et al., 2009). Ozawa et al. (2012) reported a significant inverse association between potassium, calcium, and magnesium intake and all-cause dementia and vascular dementia; the lower the intakes, the greater the rates of dementia. Dickinson et al. (2006) evaluated the effects of magnesium supplementation for the treatment of hypertension and concluded that the evidence was weak for a significant effect. In general, Kass et al. (2012). reported that higher doses of magnesium led to greater reductions in blood pressure. MUG: GI, MS, MC, and XY.

#### 6.2.18 Manganese (Mn)

Manganese is an essential dietary element in human health (Chen, 2018; Erikson and Aschner, 2019). It is an essential co-factor in carbohydrate, protein and lipid metabolism as well as a co-factor in blood clotting and is also important in osteogenesis (Selinus, 2005). It plays an essential role in bodily defence systems and the brain (Horning et al., 2015). Overexposure can lead to toxicity in the brain and neurological impairment (Erikson and Aschner, 2019). It is not only used to treat manganese deficiency, but also to treat osteoporosis (brittle bones) (Carver, 2019). MUG: NC and MS.

#### 6.2.19 Mercury (Hg)

Mercury is a toxic element (Genchi et al., 2017). It exists in nature primarily as elemental mercury or as a sulphide and is found in the Earth's crust at approximately 0.5 ppm. Atmospheric exposures occur from outgassing from rock or through volcanic activity. Human sources of atmospheric mercury include coal burning and mining (mercury and gold in particular). Atmospheric elemental mercury settles in water, where it is converted by microorganisms into organic (methyl or ethyl) mercury, which is then ingested by smaller creatures which are eventually consumed by larger fish. Fish at the top of the food chain (*e.g.*, tuna, swordfish, or shark) may concentrate a considerable amount of mercury in their tissues (Bernhoft, 2012) through bioaccumulation. Acute exposure to a large quantity of mercury vapour induces pneumonitis — inflammation of lung tissue. Symptoms of low-grade chronic exposure are more subtle and nonspecific: weakness, fatigue, anorexia, weight loss, and gastrointestinal distress (micromercurialism — chronic health effects from long-term exposure). At higher exposures, the mercurial fine tremor punctuated by coarse shaking occurs; erethism (excessive sensitivity), gingivitis, and excessive salivation have also been described, as has immune dysfunction (Kester et al., 2011). It is a risk factor in autism (Kern et al., 2016). The major route of human exposure to methylmercury (MeHg) (Newland et al., 2015) is through eating contaminated fish, seafood, and wildlife which have been exposed to mercury through ingestion of contaminated lower organisms (Rice et al., 2014). Mercury has profound cellular, cardiovascular, haematological, pulmonary, renal, immunological, neurological, endocrine, reproductive, and embryonic toxicological effects (Rice et al., 2014). MUG: MA, MN, UR, OC, and XY.

#### 6.2.20 Molybdenum (Mo)

Molybdenum has been identified as an essential trace element for nearly all plants and animals, occurring as a co-factor in three important enzymatic reactions that take place in virtually all forms of life (Novotny, 2011). Clinically, molybdenum deficiency is rare, but inborn errors of metabolism resulting in deficiencies of the molybdoenzymes have been described. Dietary intake of molybdenum is generally sufficient, with legumes such as lentils, beans, and peas being the richest source. Nuts, grains, cauliflower, and leafy vegetables are also good sources, whereas animal products and fruit are low in molybdenum. Molybdenum content of plant-based foods is dependent on the amount of molybdenum in the soil in which they are grown. Molybdenum supplementation may be of therapeutic benefit in patients with molybdoenzyme deficiency, sulphite sensitivity, Wilson's disease, and certain types of cancer, and in those receiving total parenteral nutrition (Basri, 2006). Toxicity is also associated with copper (Institute of Medicine (US) Panel on Micronutrients, 2001). MUG: XY.

#### 6.2.21 Nickel (Ni)

Although nickel is ubiquitous in the environment, its functional role as a trace element for animals, including human beings, has not been yet recognised (Genchi et al., 2020). Nickel contact can cause a variety of side-effects on human health, such as allergy, cardiovascular and kidney diseases, lung fibrosis, and lung and nasal cancer (Zdrojewicz et al., 2016). In excess it can lead to: contact dermatitis; headaches; gastrointestinal manifestations; respiratory manifestations; lung fibrosis; cardiovascular diseases; lung cancer; nasal cancer; and epigenetic effects (Institute of Medicine (US) Panel on Micronutrients, 2001). MUG: CV and RE.

#### 6.2.22 Phosphorus (P)

Phosphorus occurs in a range of phosphate-bearing minerals, usually as an accessory element including in the mineral apatite. Next to calcium, phosphorus is the most abundant mineral in the body. 85% of the body's phosphorus is in bones and teeth. Phosphorus helps filter out waste in the kidneys Berner (1997) and plays an essential role in how the body stores and uses energy. It also helps reduce muscle pain after a workout. Phosphorus is needed for the growth, maintenance, and repair of all tissues and cells, and for the production of the genetic building blocks, DNA and RNA. Phosphorus is also needed to help balance and use other vitamins and minerals, including vitamin D, iodine, magnesium, and zinc.

Most people get plenty of phosphorus in their diets. The mineral is found in milk, grains, and protein-rich foods. Some health conditions, such as diabetes, starvation, and alcoholism can cause decreased concentrations of phosphorus in the body. The same is true of conditions that make it hard for people to absorb nutrients, such as Crohn's disease and coeliac disease (Ritz et al., 2005). Some medications can cause loss in phosphorus, including some antacids and diuretics (water pills). Symptoms of phosphorus deficiency include loss of appetite, anxiety, bone pain, fragile bones, stiff joints, fatigue, irregular breathing, irritability, numbness, weakness, and weight change (Calvo and Uribarri, 2013). In children, decreased growth and poor bone and tooth development may occur. Having too much phosphorus in the body is more common than having too little and is generally caused by kidney disease or by consuming too much dietary phosphorus and not enough dietary calcium. Several studies suggest that higher intakes of phosphorus are associated with an increased risk of cardiovascular disease. As the amount of phosphorus eaten rises, so does the need for calcium. The delicate balance between calcium and phosphorus is necessary for proper bone density and prevention of osteoporosis. Phosphates (phosphorus) are used clinically to treat the following: Hypophosphatemia, deficiency of phosphorus in the body; hypercalcemia, high blood calcium contents; and calcium-based kidney stones (Yu et al., 2020). MUG: MS, OC, UR, CV, and MN.

#### 6.2.23 Potassium (K)

Total body potassium content and proper distribution of potassium across the cell membrane are of critical importance for normal cellular function (Weaver, 2013) but disorders of altered potassium homeostasis are common. It can cause kidney disease (Palmer and Clegg, 2016; Rodan, 2017). Grover and Garlid (2000) indicate a potential use of potassium in the treatment of cardiovascular disease (Houston and Harper, 2008; Roman et al., 2014; Stone et al., 2016). MUG: CV and UR.

#### 6.2.24 Selenium (Se)

Selenium is an essential trace element (Kiełczykowska et al., 2018; Rayman, 2020). Recent studies have indicated that selenium exerts a beneficial effect on coronary disease mortality, and that selenium plus garlic produce significant anticancer activity (Brown and Arthur, 2001; Avery and Hoffmann, 2018). Selenium is primarily taken up from the soil by plants as selenate (SeO_4_^2−^) or selenite (SeO_3_^2−^) (note — not the mineral selenite that is a variety of gypsum). The assimilation of selenate appears to follow the sulphate reduction pathway common to higher plants. Cereals and forage crops convert selenium into mainly selenomethionine and incorporate it into protein (Selinus, 2005). Selenium is of fundamental importance to human health. It is an essential component of several major metabolic pathways (Brown and Arthur, 2001). Whilst selenium is an important element in bodily function of the general population, the employment of Se supplements for prevention of hepatopathies has been questioned, because benefits of Se supplementation are still uncertain, and their indiscriminate use could generate an increased risk of Se toxicity (Navarro-Alarcon and Cabrera-Vique, 2008). Selenium deficiency plays a role in a number of diseases including cardiovascular conditions and cancer (Navarro-Alarcon and Cabrera-Vique, 2008; Nogueira and Rocha, 2011). There is also evidence that Se has a protective effect against some forms of cancer; that it may enhance male fertility; decrease cardiovascular disease mortality, and regulate the inflammatory mediators in asthma (Brown and Arthur, 2001). A deficiency can cause reproductive disorders in humans and animals (Mehdi et al., 2013). MUG: AN, GY, CV, and RE.

#### 6.2.25 Sodium (Na)

Sodium minerals are mainly in the form of salt (halite) (NaCl) that occur naturally and commonly in nature. It is used extensively as a preservative for food (Edwards and Marsh, 2000). Whilst sodium is an important element for bodily functions, excessive sodium intake increases blood pressure, and hypertension is a risk factor for cardiovascular disease (T. Wang et al., 2020; Mente et al., 2021). Dietary sodium intake, mainly from the excessive use of salt (sodium chloride) has received considerable attention as a potential risk factor of cardiovascular disease (Grillo et al., 2019). MUG: CV.

#### 6.2.26 Strontium (Sr)

In the body, strontium behaves very much like calcium. A large portion of the strontium will accumulate in bone (Nielsen, 2004). Strontium may be used as a supplement for the treatment of osteoporosis (Marie, 1996; Nielsen, 2004) and tooth sensitivity. However, too much strontium may result in kidney problems (Cohen-Solal, 2002). The presence of strontium in the soil *via* water, or taken up by plants and then through food and drink, will increase the prevalence of rickets. This is because it will prevent calcium absorption as well as preventing the production of the active form of vitamin D (Ozgür et al., 1996). MUG: MS, UR, and OC.

#### 6.2.27 Sulphur (S)

Sulphur is an important element in many minerals, especially sulphide ore minerals. Sulphur is the sixth most abundant macro-mineral in breast milk and the third most abundant mineral based on percentage of total body weight (Parcell, 2002). Researchers who have examined the role of sulphur in biological systems have controlled the amount of sulphur intake through regulation of protein intake. Sulphur compounds can be used therapeutically in the treatment of a number of conditions, such as depression, fibromyalgia, arthritis, interstitial cystitis, athletic injuries, congestive heart failure, diabetes, cancer, and AIDS. Mainly organically linked sulphur compounds (Alfieri et al., 2022). MUG: MC, MS, UR, and CV.

#### 6.2.28 Tellurium (Te)

Metalloid tellurium is characterised as a chemical element belonging to the chalcogen group, without known biological function (Doulgeridou et al., 2020). Recent evidence suggests that increasing environmental pollution with tellurium has a causal link to autoimmune, neurodegenerative and oncological disease (Vávrová et al., 2021). MUG: NC and XY.

#### 6.2.29 Titanium (Ti)

Titanium has a limited role in natural bodily functions. TiO_2_ nano-particles can induce pathological lesions of the liver, spleen, kidneys, and brain. Most of these effects may be due to the use of very high doses of TiO_2_ (Shi et al., 2013). Its recent exposure as a potential carcinogen to humans has also been documented (Racovita, 2022). In modern medicine titanium is a commonly used inert bio-implant material within the medical and dental fields (Kim et al., 2019). It appears not to have been used in ancient medicine. MUG: OR.

#### 6.2.30 Zinc (Zn)

Zinc is a common metallic ore, usually as sphalerite (zinc sulphide) and often co-occurs with lead (also as a sulphide, galena) (Edwards et al., 2010). It is a major player in the creation of DNA, growth of cells, building proteins, healing damaged tissue, and supporting a healthy immune system (Prasad, 2009). Zinc is found in cells throughout the body. It is needed for the body's defensive (immune) system to properly work. It plays a role in cell division, cell growth, wound healing, and the breakdown of carbohydrates (Kogan et al., 2017). A syndrome of anaemia, hypogonadism and dwarfism has been linked to zinc deficiency (Roohani et al., 2013). Compared to adults, infants, children, adolescents, pregnant and lactating women have increased requirements for zinc and thus, are at increased risk of zinc depletion (King and Cousins, 2006).

Due to the multitude of basic biochemical functions of zinc in the cells of the human body, there is a broad range of physiological signs of zinc deficiency. These signs vary depending on the severity of the condition. Organ systems known to be affected clinically by zinc deficiency states include the epidermal, gastrointestinal, central nervous, immune, skeletal, and reproductive systems (Hambidge and Walravens, 1982). Zinc is used for zinc deficiency, diarrhoea and Wilson's disease. Zinc is also used for acne, diabetes, anorexia, burns, and many other purposes. There is some scientific evidence to support its use for some of these conditions (Hambidge and Walravens, 1982). Zinc oxide topical cream is used to protect skin from being irritated and wet. Zinc compounds were probably used by early humans, in processed and unprocessed forms, as a paint or medicinal ointment, but their composition is uncertain (De Liedekerke, 2006). It is a proven astringent and antiseptic and has wound-healing effects (Reich et al., 2022). MUG: GI, NC, MS, GY, and XY.

## 7 Identification of minerals and other substances in ancient recipes

### 7.1 Introduction

We now comment on some of the other minerals and non-plant materials mentioned in Dioscorides that have implications for the identification of minerals in John the Physician's *Therapeutics*. In this paper we deal only with those materials in which we have arrived at an identification. Each account follows a standard format in which the JCLM, the lemma tag for minerals in JC (Table 1), (*e.g.,* JCLM_029) is followed by the identification in terms of modern mineralogy (*e.g.,* *antimony*), followed by the transliterated word from JC (*e.g.,* *stibi*). This is followed by the relevant information from Dioscorides including the name of the mineral that we suggest as an identification (*e.g.,* antimony), the Greek name (στίβι, *stibi*), and then the relevant quotation from DMM (Beck, 2005, Table 2). Comments follow this.

### 7.2 Identification of minerals

#### 7.2.1 Aluminium

*JCLM_030*: *στυπτερίαν astringent substance containing either alum or ferrous sulphate (stuptēria)*

Dioscorides V, 106 στυπτηρία (stuptēria), alum

“1. Nearly every kind of alum is found in the same mines in Egypt. But it also occurs in other places as in Melos, Macedonia, Lipari, Sardinia, Hierapolis of Phrygia, Libya, Armenia, and in many other places just like red earth” (Beck, 2005).

Alum is a hydrated sulphate of aluminium but can also include potassium sulphates, as well as sodium and ammonium-rich species, and may occur with evaporite minerals.

#### 7.2.2 Antimony

*JCLM_029*: *στίβην antimony (stibē)* (Fig. 2q)

Dioscorides V, 84 στίβι (*stibi*), antimony

“Antimony is excellent if it glitters and shines, if it is laminated when broken, containing neither soil nor dirt, and if it is easily broken. …It is burned placed on coals and blown upon until it catches fire: for if it should burn too much, it melts like lead. It is washed like calamine and burnt copper. But some people wash it the same way as lead dross” (Beck, 2005).

Stibnite is a rare sulphide but does occur associated with copper, zinc and lead sulphides in Cyprus.

#### 7.2.3 Arsenic

*JCLM_006*: *ἀρσενίκιν yellow orpiment (arsenikin)* (Fig. 2u)

Dioscorides V, 104 ἀρσενικόν (*arsenikon*), yellow orpiment

“Yellow orpiment is formed in the same mines as red sulphide of arsenic. That which is laminated, golden in color, with laminas that flake off and are as if lying on one another, and which is pure must be thought to be excellent. Such is the one formed in Mysia, at the Hellespont. There are two kinds of it. The one is as described above; the other is lumpy, bright red in colour, and it is brought from Pontus and Cappadocia” (Beck, 2005).

Arsenic minerals are not common in Cyprus but the description of the mineral in Dioscorides suggests that this is a correct identification. It is a mineral that is a vivid golden orange to brown with a good cleavage (Boegel, 1971). It traditionally has been used as a cosmetic.

#### 7.2.4 Asbestos (not in JC)

Dioscorides V, 138 λίθος ἀμίαντος (*lithos amiantos*), asbestos

“Asbestos is produced in Cyprus, it resembles cleft alum, and because it is fibrous, the people who work with it there make fabrics from it for shows” (Beck, 2005).

Asbestos does occur in Cyprus but it is relatively rare (Cullis, 1924).

#### 7.2.5 Clay, earth, rock and stone

*JCLM_009*: *βῶλον lump of earth (bolos)*

Not mentioned in Dioscorides

This may refer to a range of earth or clays, some of which were given specific names according to their provenances (MacGregor, 2013).

*JCLM_016*: *κισσήριν pumice stone (kissērin)*

Dioscorides V, 108 κίσηρις (*kisēris*), pumice stone

“1. One must prefer pumice stone that is very light and porous, cloven and free of stones. Additionally, it should be friable and white” (Beck, 2005).

Some pumice from geological formations in Cyprus may have been available but it will have been readily available from nearby volcanic islands in the Mediterranean and may have even floated across to Cyprus from there.

*JCLM_017*: *σφραγίδα Lemnian earth (sphragida)*

Dioscorides V 97 Λημνία γῆ (*Lēmnia gē*), Lemnian earth

“1. What they call Lemnian earth is brought up from a certain cavernous underground passage and goat, call it *sphragis*. 2. It is an uncommonly effective antidote for deadly poisons when drunk with wine, and, when taken ahead of time, it forces one to vomit the poisons. It is suitable both for the strokes of venomous animals and for their bites. It is mixed with antidotes. Some use it even in mystic rites” (Beck, 2005).

A range of earths have been used in pharmaceutical recipes since before the time of Dioscorides (MacGregor, 2013). Lemnian earth could also represent the weathering product of a range of rock types.

*JCLM_019*: *λίθος stone (lithos)*

Not mentioned in Dioscorides

This could represent a wide variety of rock types.

*JCLM_021*: *μάρμαρον marble (marmaron)*

Not mentioned in Dioscorides

This a metamorphosed limestone that is widely available in Cyprus (Cullis, 1924) but may also refer to any polished stone.

*JCLM_025*: *πετραίους of rock (petraios)*

Not mentioned in Dioscorides

There is no information about which kind of rock this represents.

*JCLM_032*: *σφραγίδα type of medication with a certified seal on it, commonly clay (sphragida)*

Not mentioned in Dioscorides

Clays have played an important role in ancient medicinal practice (Eadie, 2004; Gomes and Silva, 2007; Carrasco and Liñan, 2013; Photos-Jones et al., 2015; Gomes et al., 2021) and there is increasing interest in their contemporary use (Bryant et al., 2023) with some types even being attributed with antibacterial properties (Williams et al., 2021). They have often been termed *terra sigillata* (MacGregor, 2013) because they were certified with a seal. A range of seals have been discovered, with each seal denoting a particular product used for a particular condition. Although all medicinally used clays seem to be smectites, the diversity of them in use in antiquity makes it difficult to assess the kind of clay used if no further details are given.

#### 7.2.6 Coal

*JCLM_015*: *καρβούνια coal (karbounia)*

Not mentioned in Dioscorides

Coal is not mentioned in Dioscorides and as there is no coal in Cyprus, the term might rather refer to charcoal or glowing embers of a wood fire.

#### 7.2.7 Copper

*JCLM_014*: *καλακάνθην blue vitriol (kalkanthē)*

Dioscorides V, 98 χαλκανθές (*khalkanthes*), copper sulphate solution

“There is a single kind of copper sulphate solution: for it is a congealed liquid. …The best is blue and heavy, dense, clean and translucent; such is the *stalacton*, which some call *lonchoton*. Then comes the *pecton;* but the *ephthon* is popular for dying and for black hair dyes; experience has proven, however, that it is very weak for medical use” (Beck, 2005).

Blue vitriol (often referred to as Hungarian vitriol) is essentially a copper sulphate mineral that is produced artificially by treating copper with hot sulphuric acid. It is possible that it could have been produced in ancient copper mines.

*JCLM_026*: *πυρίτης a stone that strikes fire (puritēs)* (Fig. 2e)

Dioscorides V 125 πυρίτης λίθος (*puritēs lithos*), copper pyrites

“1. Copper pyrites is a type of stone from which copper is mined. You must choose that which is copper-coloured and which emits sparks readily. You must burn it this way: after coating it with honey place it on gently burning coals and fan it continuously until it becomes orange-tawny in colour”. (Beck, 2005).

*JCLM_039*: *χαλικολίθαρον (khalikolitharon) literally copper/bronze stone*
*JCLM_041*: *χαλκόν copper or bronze (khalkos)*

Dioscorides V, 76 κεκαυμένος χαλκός (*kekaumenos khalkos*), burnt copper

“Burnt copper is good when it is red and of the color of cinnabar when rubbed; but if it is black, it has been burned more than it should have been…The best is that burned in Memphis, then that burned in Cyprus” (Beck, 2005).

A range of techniques in producing copper and metals from ore provides a variety of possible minerals with a wide range of composition.

The following copper minerals are in DMM, but not in JC:

Dioscorides V, 77 χαλκοῦ ἄνθος (*khalkou anthos*), flower of copper
  Flower of copper, which some old writers have called nail- scrapings, is excellent …It is made this way: when the copper that was smelted in the ore furnaces flows down through the strainers of the conveying ducts into the receptacles, the attendants, after removing the dirt, pour on it very fresh water, their purpose being to cool it” (Beck, 2005). He goes on to say “Applied ground up with honey, it shrinks both uvulas and tonsils”, as well as considering other medical uses
Dioscorides V, 79 ἰός ξυστός (*ios xustos*), verdigris (copper acetate formed by the action of acetic acid, *e.g.,* vinegar)
  “You must prepare verdigris this way: after pouring very sharp vinegar into a cask or into another similar container, cover it with an inverted copper vessel; it is good if the cover were concave, but if not, it may be flat. It should also be clean and tight fitting. After ten days, removing the cover, scrape off the verdigris that coats it” (Beck, 2005). “And they say that verdigris is also formed in Cypriot mines, one type forming on the surface of some stones that contain copper and the other drips from a certain cave during the burning heat of the Dog Star” (Beck, 2005).
  The number of times copper is mentioned in Dioscorides' description of pharmaceuticals that are also mentioned in JC suggests that it must have played an as yet unidentified role in at least some of the JC recipes. The prescription of a copper pestle is interesting as it has been found that what is used to crush minerals in particular can affect their properties (Principe, 2016). The problem may be that verdigris may be a natural oxidation product of copper (*e.g.,* the common green patina seen on copper objects) or else copper acetate or even copper carbonate. The essential element here is copper.
Dioscorides V, 90 Ἀρμένιον (*Armenion*), azurite (Fig. 2g, h)
  Dioscorides writes “Azurite: you must choose it smooth, of a deep and very even blue color, without stones, easily broken, and as thick as chrysocolla. It accomplishes the same results as chrysocolla, falling short of it only in efficacy” (Beck, 2005).
  Note azurite (Fig. 2g), which is a copper carbonate mineral, may be confused with some lapis lazuli (see Section 3).
Dioscorides V, 103 διφρυγές (*diaphruges*), pyrite from copper mines
  Dioscorides writes “One must suppose that pyrite from copper mines is of three kinds: for one is a mineral, occurring, in fact, only in Cyprus” (Beck, 2005). He also notes: “After burning ochre, people sell it as pyrite from copper mines” (Beck, 2005).
  Iron and copper are likely to be a common element in recipes. Pyrite, both iron and copper, is found in vast quantities in Cyprus (Fig. 2; Edwards et al., 2010). Copper production is common in Cyprus and copper pyrites is one of the principal ores.

#### 7.2.8 Glass

*JCLM_034*: *ὑάλιν glass (hyalin)*

Not in Dioscorides

Formed from molten sand. Could refer to natural volcanic glass that is found in Cyprus.

#### 7.2.9 Gold

*JCLM_041* and *JCM_2876*: *χρυσάφιν gold (chrysaphin)*

Not in Dioscorides

This is rarely found in Cyprus but easy to distinguish from iron pyrite.

#### 7.2.10 Iron

*JCLM_027*: *σίδηρον iron (sidēron)*

This may refer to one of the many iron minerals that occurs commonly in Cyprus ranging from sulphides to oxides (Fig. 2).

*JCLM_028*: *σκουρέαν iron (III) oxide (Fe_2_O_3_) (skouria)*

Dioscorides V, 80 ἰός σιδήρου Iron rust (ios sidērou)

Dioscorides comments on several medicinal uses, for example: “1. Iron rust binds and stems leucorrhea, when applied, and it causes barrenness when drunk. Smeared on with vinegar, it effectively treats erysipelas and pustules, it is useful for whitlow, for membranes that grow over the inner corners of the eye, for rough eyelids, and for callous lumps” (Beck, 2005).

Essentially this represents some kind of iron oxide that widely occurs in Cyprus.

*JCLM_001*: *αἱματίθην hematite (haimatitēs)* (Fig. 2r)

Dioscorides V, 126 αἱματίτης λίθος (*haimatitēs lithos*)

In the text no visual description is given, and the location is given as occurring in Egypt. A brief description of the physical properties is given. “Hematite is of excellent quality when it breaks easily as if of its own accord and when it is hard, uniformly strong, and free of any dirt or veins” (Beck, 2005). It appears to have both external as well as internal uses. A description of the mineral being burned is given: “It is burned like Phrygian stone but without the wine. Let the standard for burning it be that it becomes moderately light and full of bubbles” (Beck, 2005). One feature of potential use is the description of colour: “It is found in Sinopic red earth, it is also made from magnet that was burned for a while, but natural hematite is mined in Egypt” (Beck, 2005).

It is true that many iron minerals may oxidise to a red colour by weathering but also soils may be red if they are subjected to fire. The identification of such materials has often caused difficulty to ancient writers (Scott and Freedberg, 2000; Scott, 2001).

The following iron minerals are in DMM, but not in JC:

V, 93 ὤχρα (*ōkhra*), yellow ochre
  Dioscorides writes “You must select yellow ochre that is very light, quince-yellow throughout, of a deep colour, free of stones, easily broken, and of Attic provenience. It, too, must be burned and washed like calamine” (Beck, 2005).
  This is an oxide of iron and likely to be common in Cyprus.
V, 96 μίλτος Σινωπική (*miltos Sinōpikē*), Sinopic red earth
  “Sinopic red earth is excellent if dense and heavy, liver-colored, free of stones, evenly coloured, and capable of much diffusion in solution. It is collected in Cappadocia, in certain caves, and after it is refined, it is brought to Sinope and sold, whence it also received its surname” (Beck, 2005).
  This is one of the many types of earth that are widely used (Gomes and Silva, 2007; Carrasco and Liñan, 2013). Many red earths occur in Cyprus.

#### 7.2.11 Lead

*JCLM_018*: *λιθάργυρον litharge* (*litharguron*)

Dioscorides V, 87 λιθάργυρος (*litharguros*), litharge

Litharge is considered to be lead oxide, and hence an oxidation product of the mineral galena. Dioscorides provides some description of the mineral and potential occurrence as well as indicating an external use. “Litharge: there is one type that is produced from the sand called *molybditis* when it is smelted until its complete calcination, another from silver, and another from lead. …It has properties that are astringent, emollient, that cool, that are capable of stopping the pores, of filling up hollows, of controlling fleshy excrescences, and of healing” (Beck, 2005).

There is, however, no certainty that this represents a single mineral, but it may well include the secondary mineral, lead oxide, known by that name.

*JCLM_024*: *μολύβδιν lead (molubdin)* (Fig. 2m)

Lead in its many forms has been used in a range of treatments but little data is provided as to the form of the mineral considered. It could represent lead produced by the smelting of galena that occurs in Cyprus.

*JCLM_043*: *ψιμμίθιον white lead (psymmithion)*

*Dioscorides* V, 88 ψιμύθιον (*psimuthion*), white lead

Dioscorides comments: “White lead is made this way: pouring into a wide-mouthed jar or into a pot-bellied clay jar very strong vinegar, and after lining the mouth of the jar with a mat of reeds, place on the jar's mouth a lead block and throw over it coverings to prevent the vinegar from evaporating. When the lead block sinks down crumbling, you must, on the one hand, strain the clean liquid that floats on top and, on the other hand, you must pour the viscous part into a container and dry it in the sun” (Beck, 2005).

As noted earlier some lead minerals occur in Cyprus and some may have been converted to elemental lead.

*JCLM_031*: συρικόν either “*bright red mineral colourant from red lead*” or alternatively a misspelled word that means “*silk*”. We think it is the former (*surikon*)

As previously indicated lead minerals and a range of red oxide minerals occur frequently in Cyprus.

Terms relating to lead that are in DMM and not in JC:

Dioscorides V, 81 πεπλυμένος μόλυβδος (*peplumenos molubdos*), washed lead
  Dioscorides provides several recipes to prepare washed lead thus indicating that it may be widely used but does not provide evidence of medicinal use.
  There are a number of lead minerals, each with a different preparation and potential use. Whilst lead seems to be a major priority for description in Dioscorides it appears much less frequently in JC.
Dioscorides V. 82 σκορία μολύβδου (*skoria molubdou*), lead dross
  ‘Lead dross that looks like white lead, that is thick and difficult to break, that contains nothing lead-like, but that is quince-yellow and glassy is excellent. It is good for the same things as burnt lead, but it is more astringent’ (Beck, 2005).
Dioscorides V, 83 ὁ μολυβδοειδὴς λίθος (*molubdoeidēs lithos*), leadstone
  Dioscorides writes “Leadstone has properties that are comparable to those of lead dross and it is similarly washed” (Beck, 2005).
Dioscorides V, 85 μολύβδαινα (*molubdaina*), galena (Fig. 2m)
  Dioscorides writes “1. Galena that looks like litharge, that is blonde, somewhat shining, orange-tawny when triturated, and liver-coloured when cooked in oil, is of excellent quality. But it is bad when it is grey or leaden. It is made from silver and from gold. There is also a kind that is mined, found around Sebastes and Corycos; of this one, too, the best is neither dross-like nor pebbly but blonde and shiny” (Beck, 2005).

#### 7.2.12 Lime

*JCLM_007*: *ἀσβέστιν lime (asbestin)*

Dioscorides V, 115 ἄσβεστος (*asbestos*), unslaked lime

Following a description of how the material is made he comments “All unslaked limes have in common properties that heat, bite, burn, and that further the formation of eschars. But when combined with certain other ingredients, such as suet or olive oil, they can further maturation, soften, disperse, and cicatrize. That which is fresh and not wet must be considered the most effective” (Beck, 2005).

Lime, *i.e.,* calcium carbonate, makes up not only the shells of molluscs (*e.g.,* mussels for example) but also limestones and marbles.

*JCLM_023*: *νίτρον sodium carbonate (nitron)*

Dioscorides V, 113 νίτρον (*nitron*), soda

“One must choose soda that is light and either rosy or white in color, having holes as if it were a spongy substance” (Beck, 2005).

We might consider here that it is lime (calcium carbonate) mixed with seawater containing sodium chlorides that is burned to form a sodium carbonate that is the essential ingredient of Plaster of Paris. The natural mineral natron is found in Egypt but not recorded from Cyprus.

#### 7.2.13 Magnetite

*JCLM_020*: *μαγνήτιν magnet (magnetis)*

Dioscorides V, 130 μαγνίτης λίθος (*magnitēs lithos*), magnet

Dioscorides comments “The best magnet attracts iron easily, is bluish in color, dense, and not too heavy. It has a property that drives out thick masses when given in the amount of one *triobolon* with hydromel. Some people burn it and sell it as hematite” (Beck, 2005).

This could refer to the mineral magnetite that does occur in Cyprus.

#### 7.2.14 Mercury

*JCLM_035*: *ὑγράργυρον mercury (hygrargyron)*
*JCLM_038*: *ὑδράργυρον mercury (hudrarguros)*

Dioscorides V 95 ὑδράργυρος (*hudrarguros*), mercury

“1. Mercury is made from what is called *minion*, which is wrongly called also cinnabar: for they place an iron spoon that has cinnabar on a clay vessel, they turn over it a cup luting it with clay, and burn it on coals. For the soot that settles on the cup becomes mercury when scraped off” (Beck, 2005). He comments on medicinal use: “Large quantities of milk drunk and vomited help, as does wine with wormwood *artemisia absinthium*, or seed of celery, or clary and either oregano or hyssop with wine” (Beck, 2005).

Cinnabar is the main sulphide ore of mercury.

Dioscorides V, 94 κιννάβαρι (*kinnabari*), cinnabar

Dioscorides writes: “Some are wrong in thinking that cinnabar is the same as that which is called *minion*: for *minion* is made in Spain from a certain stone that is mixed with sand containing silver ore. This stone is otherwise unknown, but in the furnace it changes into a very bright and flaming color. It does have a suffocating smell among minerals. At any rate, the natives there place around their faces bags to enable them to see but not to breathe the air” (Beck, 2005). He goes on to comment: “Cinnabar has the same properties as hematite, but it is a great deal more suitable for eye medications, for it is more astringent; wherefore it also staunches blood and it treats burns and pustules when compounded with cerate” (Beck, 2005).

Iron and mercury minerals may be confused when in their natural state. There is some difficulty in understanding what is meant here. Cinnabar is found in epithermal veins and associated with alkaline hot springs. There are also well-known deposits at Almaden in Spain. It also occurs as a different type known as *cinnabaris factitia* which is a smelt derivative.

#### 7.2.15 Pitch

*JCLM_036*: *ὑγροπίσση liquid pitch (hygropisse)*

Dioscorides I 72 πίσσα (*pissa*), pitch

Dioscorides comments on a range of pitches and considers a range of uses including: “It is capable of warming, softening, suppurating, dispersing growths and swellings of glands, and fleshing out sores. It is also profitably mixed with wound medications” (Beck, 2005).

It is clear in this case that what is described represents many different types of organic substance. A form of pitch may be produced during the charcoalification process.

#### 7.2.16 Salt

*e.g.,JCLM_002*: *ἅλας salt (halas)*

There are a range of salts possible here from simple sea salt to salts from evaporite deposits and their chemical composition may have bearing on their use or misuse.

Dioscorides V,109 ἅλς (*hals*), salt

“The most effective salt is that which is mined and off this kind, in general, that which is white, free of stones, translucent, dense, smooth in its formation, particularly the Ammonian in origin, that can be split, and that has straight cracks. But you must select sea salt that is dense, white, and smooth. Salt produced in Cyprus, especially in Salamis of Cyprus and in Megara is excellent” (Beck, 2005).

Note that in Cyprus there are many sources of salt, from ancient salt deposits and from modern salt lakes and salt pans.

#### 7.2.17 Serpentine

Not in JC

Dioscorides V, 143 λίθος ὀφίτης (*lithos ophitēs*), serpentine

“There is a kind that is sturdy and black, another ash-colored and spotted, and another that has white lines. All are useful when tied on people stung by vipers or on those who have headaches. The one that has the lines is reported to be particularly helpful for lethargic fever and headaches” (Beck, 2005).

Serpentine occurs in Cyprus (Cullis, 1924) so it would be expected to have been featured in the JC recipes.

#### 7.2.18 Silver

Not in JC

Dioscorides V, 86 ἡ τοῦ ἀργύρου σκορία (*hē tou argurou skoria*), silver dross

“The dross from silver is called *helcysma*. It has the same properties as galena, whereof it is mixed both with grey plasters and with plasters that cicatrize, since it is astringent and causes adherence” (Beck, 2005).

Silver is a minor element of galena (Fig. 2m) and produced when lead ores are smelted.

#### 7.2.19 Sulphur

*JCLM_012*: *θεῖον sulphur (theion)*

Dioscorides V, 107 θεῖον (*theion*), sulphur

“Sulphur that was not burned must be judged to be excellent when it is of a shiny color, transparent, and free of stones, but of the sulphur that has been burned, it is the pale-green and very fat that is excellent. Much sulphur is produced on Melos and on the Lipari islands” (Beck, 2005).

Of all minerals sulphur has the most distinctive smell and together with its colour makes identification more likely to be correct. Some sulphur may occur as native sulphur but other sulphur may have been obtained through the smelting of metal sulphides commonly undertaken in Cyprus.

*JCLM_005*: *ἄπυρον without fire (used to describe a type of sulphur)*
*JCLM_033*: *τεάφην probably sulphur (teaphin)*

Native sulphur is more likely to be the mineral than sulphur resulting from smelting processes.

#### 7.2.20 Water

JC mentions different kinds of waters which are not specified in DMM.

*JCLM_003*: *A type of water of volcanic origin (alboula)*

There are no active volcanoes in Cyprus so it is uncertain what is meant here. It could be water from hot springs that may contain chemical elements.

*JCLM_004*: *ἅλμην Salt water (halme)*

It is uncertain whether this means sea water, or water from the evaporitic lakes that occur in Cyprus (Edwards et al., 2010).

*JCLM_008*: *βροχῆς rain (brokhe)*

Rainwater differs from some fresh and salt water in chemistry and hence may be differentiated in recipes.

*JCLM_010*: *δροσᾶτον either an unusual word for dew, or rosewater (drosaton)*

Rosewater (a mixture of olive oil and rose petals) is a major component of some recipes.

*JCLM_011*: *θαλασσίαν sea water (thalassia)*

This would be readily available in Cyprus and contains natural salt (sodium chloride).

*JCLM_022*: *νερόν water (neron)*
*JCLM_038*: *water*

As distinct from rain or seawater, presumably fresh water.

*JCLM_039*: *χαλάζιν hailstone (khalazin)*

It is not clear why this might be mentioned except as a source of ice.

#### 7.2.21 Zinc

Not in JC

Dioscorides V, 75 πομφόλυξ (*pompholux*), *pompholyx*

In describing the material Dioscorides comments: “The white *pompholyx* is produced when, in processing and finishing the copper, metallurgists, wishing to improve it, sprinkle it generously with triturated calamine. For the smoke that rises from it, which is very white, forms bubbles” (Beck, 2005).

In addition, it is claimed by Dioscorides that “The Cyprian must be thought to be the best; when kneaded with vinegar, it emits a smell of copper and it is somewhat rust in colour; moreover, it has a muddy taste” (Beck, 2005). However a number of forms are described where burning is involved but Beck (2005), in the translator's notes, believes that the material described is zinc oxide. It is uncertain why this has been assumed. It would indicate that the furnace process involved sphalerite alone and that may be rather unusual. This is especially problematic as it also states that it is formed when working with copper. Most often, however, iron and copper sulphides co-occur but lead and zinc more often occur together.

It is clear that in at least this case the material is unlikely to be zinc oxide. Most of the metal smelting in ancient Cyprus concerns copper and iron sulphides with lead and zinc being much rarer (Davies, 1930; Knapp et al., 2001).

## 8 Experimental investigation of recipes

### 8.1 Introduction

In the recipes of John the Physician (JC) plants and minerals are processed in a variety of ways which may enhance the bioavailability of elements for external or internal use. There are two distinct ways in which material is processed: Using a liquid such as an acid to dissolve the material, or burning material and then using a liquid to dissolve it. The recipes tend to use vinegar (*e.g.,* recipes 12, 22) or wine (recipe 26), or in some cases warm water, as with clays or other minerals (39) even with ground up materials (62). What we do not see is extensive heating of minerals before their use.

How were the recipes prepared, to what use were the resulting materials put, and is there a detectable rationale behind the choice of materials and preparation methods? To address these issues, we undertook a series of experiments on materials the identification of which we were relatively certain. These were sourced from the authors' collections, or in the case of some of the plant material, from the living plant collection of Kew Gardens.

### 8.2 Experimental procedure

#### 8.2.1 Charring

The charring of samples used two methods:

1. Samples were broken into maximum dimensions of 2 × 5 cm or smaller and placed in steel tubes designed to prevent the ingress of oxygen whilst allowing volatile gases to escape and be vented. Charcoals were produced by heating for as long as 60 min. Only when the desired oven temperature was reached were the samples, within steel containers, placed in a thermostatically controlled oven (ambient to 600 °C). However, a data-linked thermocouple probe inserted into the samples indicated a lag time of approximately 15 min (the heat-soak time), and all charring times were recorded from this point on. In general times of 30 min and 60 min were used, at temperatures of 300 °C and 600 °C.
2. Samples were heated at 300 °C and 600 °C in the presence of air to allow the material to be ashed. Some specimens were subjected to higher temperatures, up to 1000 °C (as radiant heat) in a Carbolite CWF1100 furnace for 2 h with air included (Scott, 2020).

#### 8.2.2 Scanning electron microscopy

Scanning electron microscopy (SEM) is the most effective way to study charcoalified plant fossils (Scott and Collinson, 1978; Collinson, 1999; Figueiral, 1999; Scott, 2010). Techniques in SEM study have been widely published and will not be repeated here (Sander and Gee, 1990; Scott, 2010). The study of charcoalified plants and animals by SEM is important as three-dimensional anatomy may be well preserved. Specimens were mounted on aluminium stubs and gold coated. They were examined using a Hitachi S2400 low pressure Scanning Electron Microscope.

#### 8.2.3 Dissolution

To determine how, qualitatively and quantitatively, various metals and other elements from different minerals and burnt organic materials were able to dissolve, different media were used in controlled conditions. Materials of defined and precise weights (between 0.0025 g and 0.83 g for the 11 organic burnt samples, and around 1 g for most of the 17 minerals) have been mixed each time respectively into 25 mL of distilled water (DW), 20 mL of red wine vinegar (RWV) and 25 mL of red wine (RW). The 84 concoctions (three times 28) and three blanks (DW, RWV, RW) were shaken for 24 h on a shaking-table at 200 rpm. This recognised and controlled technique (ISO 11048:1995) accelerates the exchange between the material and the medium within a reasonable timescale. Vessels were closed during the process, although with sufficient headspace to allow some air-exchange. To replicate as best as possible the original conditions, the vessels used were glassware. The resulting solutions of the dissolved elements have been filtered to remove any particulates and acidified to stabilise the dissolved phase. The solutions have been analysed by ICP-AES (Inductively Coupled Plasma-Atomic Emission Spectrometry) (Thompson and Walsh, 1983). The elemental analysis comprises Al, As, B, Ba, Ca, Cd, Co, Cu, Cr, Fe, K, Li, Mn, Mg, Na, Ni, Pb, Sr, Ti, V, Y, and Zn. The calibration was determined with three certified multi-elemental stock-solution material references (CRMs) and four in-house RMs. Obtained data has been drift corrected, blank corrected from the instrumental background, and blank corrected from the respective mediums. The specific dilution factor has been applied to each solution to be able to calculate the elemental dissolution in mg per litre, to compare the performance for each material into the three different substances.

### 8.3 Experiments

#### 8.3.1 Animals

##### 8.3.1.1 Cuttlefish (Fig. 3)

JC 117.[98] “If there is something white on the eye. Burn the shell of cuttle fish (JCA_1305) and mix its ashes (JCX_0126) with good honey (JCA_0024) and put it into the eye. […]”

**Fig. 3:**
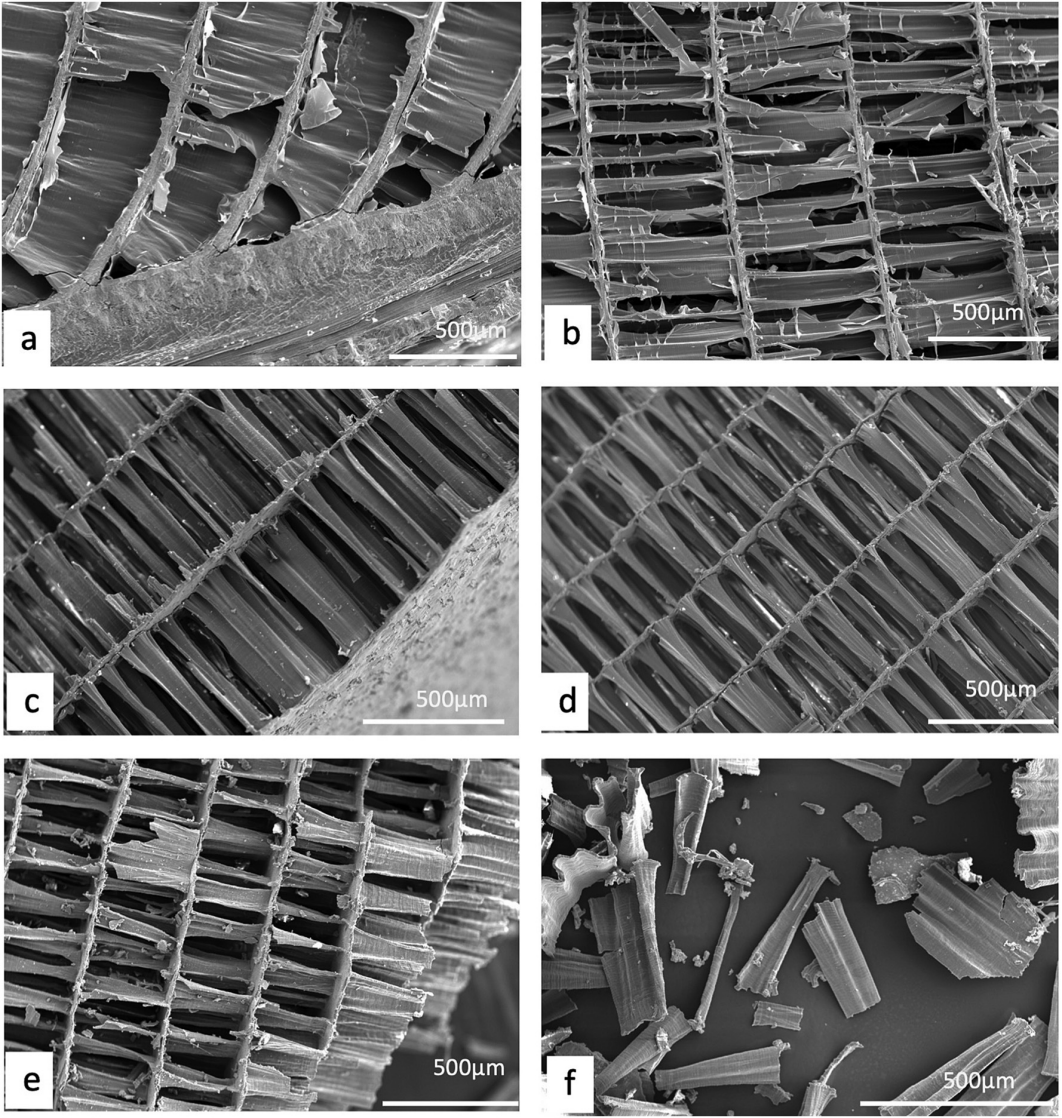
Scanning electron micrographs of cuttlefish bone. a. Uncharred cuttlefish bone. b. Uncharred cuttlefish bone. c. Cuttlefish bone charred at 300 °C for 1 h. d. Cuttlefish bone charred at 300 °C for 1 h. e. Cuttlefish bone charred at 600 °C for 1 h. f. Cuttlefish bone charred at 600 °C for 1 h — crushed showing isolated blades.

This recipe refers to the burning and use of cuttlefish bone. We, therefore, made a microscopic examination of cuttlefish bone both before and after charring.

A key question is why cuttlefish would be used and how charring affects the morphology and composition of the material. There are several important issues in relation to the charring of cuttlefish bone. The chemical composition of the ‘bone’ is calcium carbonate. If in the form of calcite, it would convert to calcium oxide when burnt. However, the cuttlefish mineral is aragonite that appears to alter less than calcite during the burning process. It is probable that the initial exposure to heat will sterilise the material that would be important if it is being put into the eye. However the structure of the ‘bone’ indicates there are numerous vertical plates with a distinct horizontal layering (Fig. 3a, b). With increasing temperature these bounding horizontal layers provide planes of weakness so that many individual shards are easily released when crushed (Fig. 3c–f). At higher temperatures these vertical plates appear to reveal a distinctive patterning that may be important in their use (Fig. 3f). At higher temperatures the individual ‘blades’ become detached and hence may be more easily suspended in honey. A next step would be to prepare the recipe and examine the behaviour of the material in the honey, in consultation with an ophthalmologist but this was not undertaken.

Simply putting such blades in the eye would likely to cause damage. However suspending the blades in honey would have two effects. Firstly only a few blades need to be suspended in the viscous liquid which would have the effect of being gentler on the eye. The blades then may be able to gently scrape away the surface spots on the eye. Secondly, it is now known that honey has antibacterial properties that may have aided healing (Root-Bernstein and Root-Bernstein, 1999; Ramsay et al., 2019).

##### 8.3.1.2 Mussel shells (Fig. 4)

JC 195. “Therapy for lichen and leprosy (sensu lato). […] 3. Burn the so-called mussel (JCA_2034) and mix the ashes (JCX_0126) with vinegar (JCLP_142) and apply. […] […]”

**Fig. 4:**
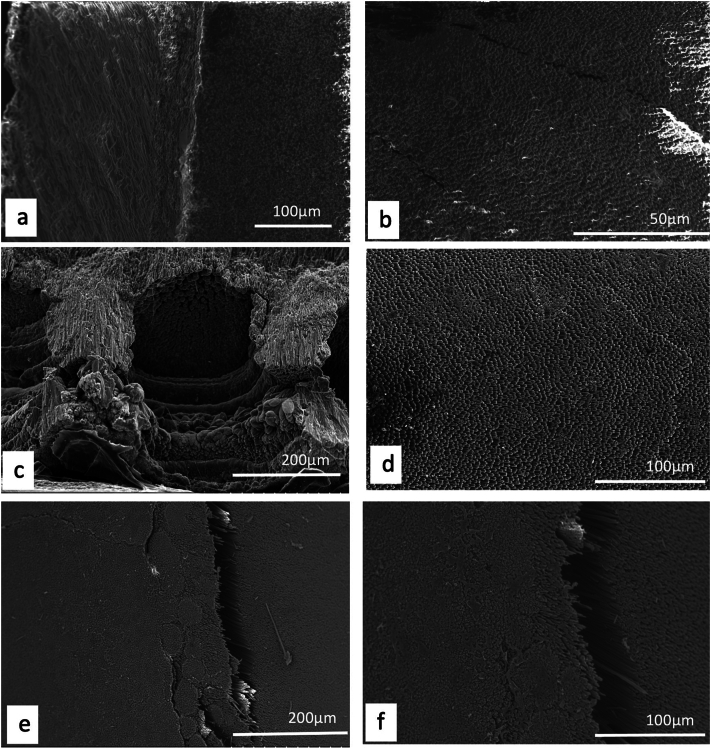
Mussel shells. Scanning electron micrographs. a. Uncharred showing fibrous shell structure. b. Uncharred. Detail of inner surface of shell. c. Charred at 300 °C for 60 min. Fibrous structure near hinge of shell. d. Charred at 300 °C for 60 min. Detail of fibrous rods in cross-section. e. Charred at 600 °C for 60 min. Showing fibrous structure still surviving. f. Charred at 600 °C for 60 min. Detail showing fibrous rods.

In this case also it is not clear why the shells of mussels have been chosen, apart from the fact that they might have been readily available. Mussel shells are composed of calcite (calcium carbonate). The distinctive shell structure was studied under SEM after burning. The nacre and main shell layer have quite distinct structures (Fig. 4a). The main shell layer is made up of distinctive crystals (Fig. 4a). The heat might convert part of the calcium carbonate mixture to calcium oxide, thus weakening the structure so that when mixed with vinegar the shell structure is broken down more easily. It is not easy to understand the reasons for this procedure. The carbonate rods (Fig. 4d) that make up the shell structure still remain intact even at 300 °C (Fig. 4c, d) and at 600 °C (Fig. 4e, f). It is possible that heating the shell makes it easier to dissolve in vinegar.

The main uses appear to be external but their relevance to leprosy is unclear. In our experiments we have shown that even with water calcium, magnesium and potassium are dissolved. Wine vinegar dissolved a wide number of elements including strontium and boron. These elements play a role both in bones and teeth so supplements may be of use for several conditions.

##### 8.3.1.3 Deer horn (Fig. 5)

JC 1.[1] “For acute headache. […] 2. Burn a bone of a deer (JCA_0366), soften it with rose (JCLP_181) olive oil (JCLP_053) and apply. Or once you have burnt the bone of a deer (JCA_0366), grind it and make it like flour. And then mix it with rose oil (JCLP_181) and apply to the forehead and the temples.”

**Fig. 5:**
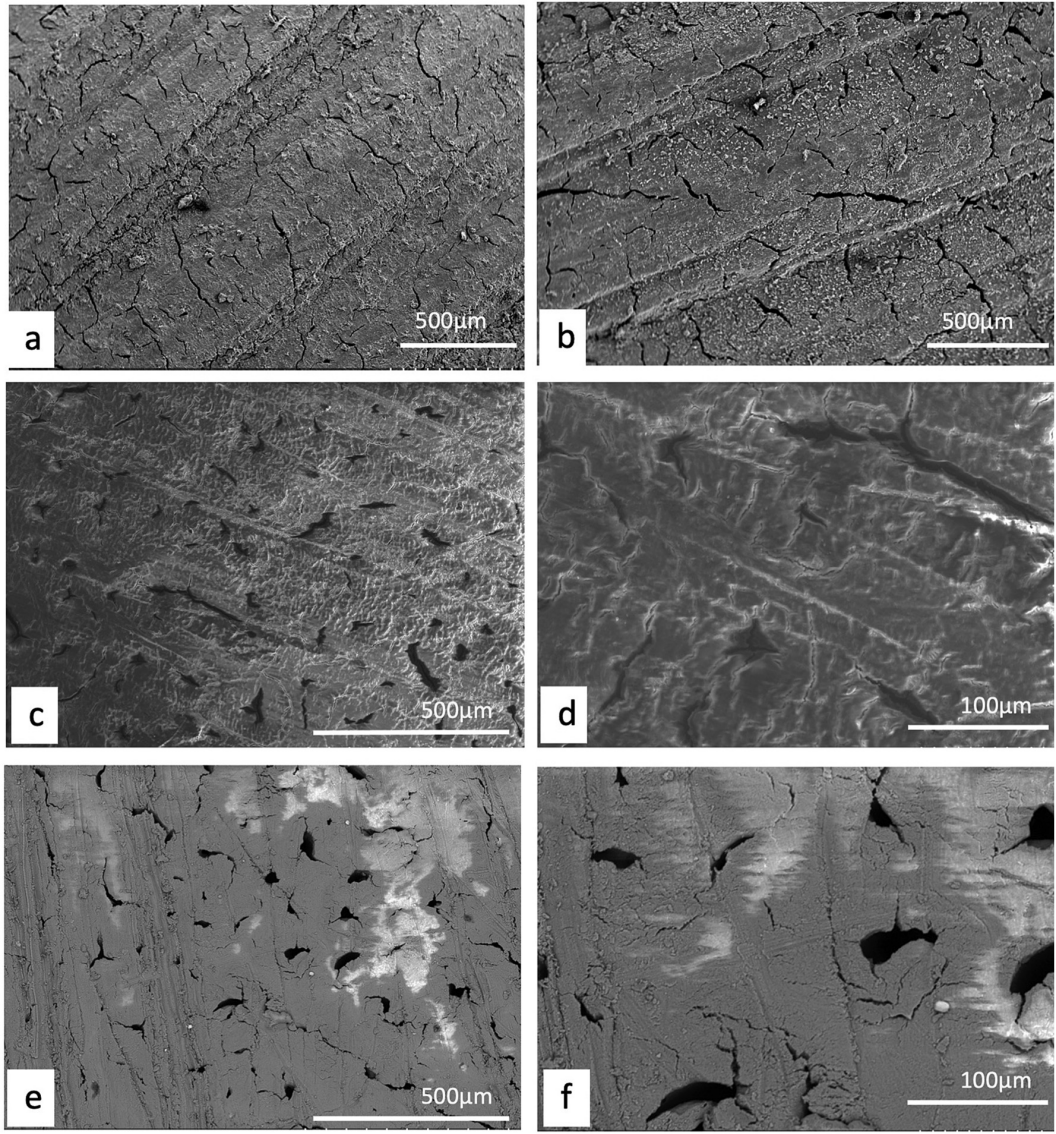
Deer horn. Scanning electron micrographs. a. Charred at 300 °C for 60 min. Cracks and saw marks visible. b. Charred at 600 °C for 60 min. Little change in structure from 300 °C. c. Charred at 600 °C for 60 min. Small holes visible but fundamental structure not changed. d. Charred at 600 °C for 60 min. Small holes visible but fundamental structure not changed. e. Charred at 900 °C for 60 min. Bone shaving. Small holes visible and material changed colour to white. f. Charred at 900 °C for 60 min. Detail of e showing small holes in bone.

JC 73.[63] “On *kelephiasis*. […] 3. Burn horn (JCA_0502) of a deer (JCA_0649) and make it fine and let him drink with wine (JCLP_144). […]”

JC 29.[26] “To make the teeth white. Burn horn (JCA_0502) of a deer (JCA_0366) until it gets white. Then grind it and make it like flour and apply to the teeth. […]”.

Dioscorides II, 59 ἐλάφου κέρας (*elaphou keras*), hart's horn

“An amount of two spoonsful of hart's horn burned and washed, is a suitable drink for people who spit blood, dysenteries, people who suffer in the bowels, for the jaundiced, for bladder pains with tragacanth, and for women who are having discharges with a liquid that is appropriate for their condition” (Beck, 2005).

There are several recipes that call for the use of deer horn. It is uncertain why it may have been used, but horns would have been easily obtained and may have been thought to have had some significance, even magical. When charred even at 600 °C for 60 min there appears to be little change in the horn structure (Fig. 5d). It was therefore deemed necessary to see how the horn reacted at higher temperatures with readily available oxygen. Charring deer horn proved more problematic. Pieces of horn were charred at 300 °C and 600 °C (Fig. 5a–d) without much apparent change. Slivers of horn were then taken and burned at 900 °C but much of the horn remained intact and showed only a few extra holes in the surface (Fig. 5e, f) and was much easier to grind as indicated by the recipe.

Solutions using wine and wine vinegar are rich in calcium, sodium, magnesium and potassium and their possible help in some conditions needs further investigation.

##### 8.3.1.4 Egg shells (Fig. 6)

JC 7.[6] “When blood is flowing from the nose. Burn the shell (JCX_1360) of an egg (JCA_0105) and grind it well and put it into a reed. And put one part of the reed into his nose and blow into the other part and it will get into the nose. […]”

**Fig. 6:**
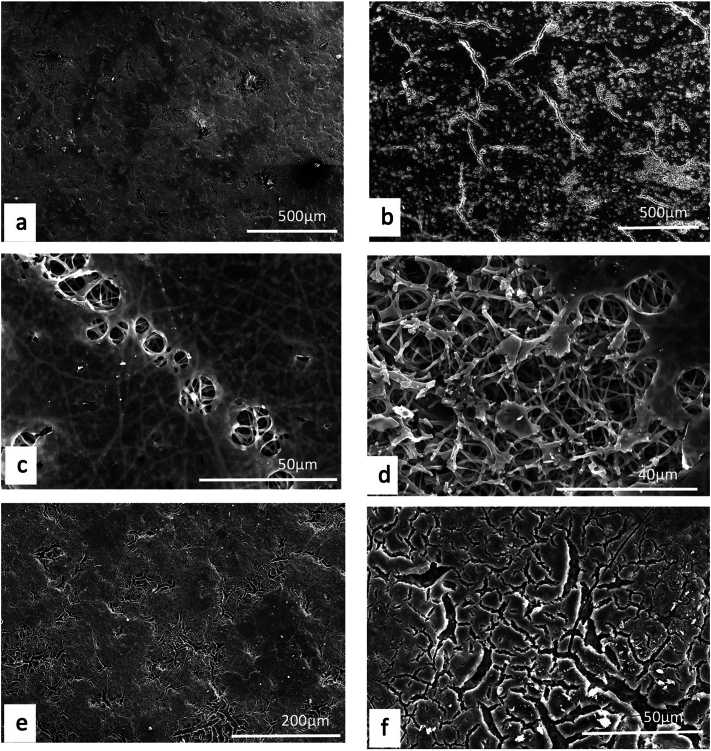
Egg shells. Scanning electron micrographs. a. Charred at 300 °C for 60 min. General external surface. b. Charred at 300 °C for 60 min. External surface showing wrinkled membrane. c. Charred at 300 °C for 60 min. Internal porous structure partially exposed. d. Charred at 300 °C for 60 min. Internal porous structure of shell. e. Charred at 600 °C for 60 min. Loss of shell structure. f. Charred at 600 °C for 60 min. Detail showing partial destruction of shell structure.

15.[14] “For clogged up ears. Put syrup into the shell (JCX_0167) of an egg (JCA_0105) and put the shell (JCX_0167) on the coals and let it become warm. Then put it into the ear. […]”.

It is not at all clear why eggshells should be burned and subsequently prepared, although calcium is important, for example, in blood clotting. The shells themselves comprise a hard outer layer made of calcium carbonate with an inner organic layer. One would presume that the burning of the shells would have had the effect of both combusting the organic layer and exposing the structure of the shell that would also be chemically altered.

Under SEM we see that the shell has a particular porous structure (Fig. 6) with many tiny pores. The eggshell remained intact at 300 °C (Fig. 6a–b) but some of the inner surface layer was stripped away (Fig. 6c–f). This revealed a highly porous structure which then could act as a ‘sponge’. At 600 °C (Fig. 6e, f) the shell structure began to show evidence of partial destruction and loss of internal porous structure.

It is possible that the fragments of shell may be used simply as a carrier of the liquid — either as an absorbent of liquid as in the use to stem blood flow or as a carrier of liquid, as would be the case with the use with syrup.

##### 8.3.1.5 Sponge (Fig. 7)

JC 28.[25] “For a lot of blood when it flows out of the nose. […] 2. Soak a new sponge (JCA_0200) in cedar oil (JCLP_083). And then burn the sponge (JCA_0923) well so that it roasts. Then make it like flour. Blow it through a reed (JCLP_075) into the nose. […]”

**Fig. 7:**
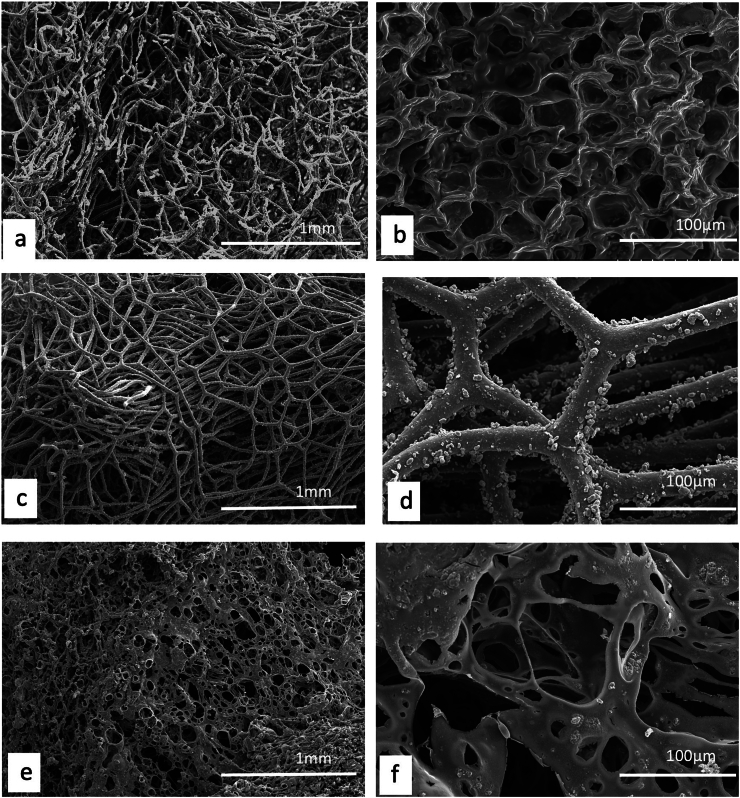
Sponges. Scanning electron micrographs a. Uncharred, showing stringy nature of the sponge. b. Uncharred, detail of area with vesicles. c. Charred at 300 °C for 60 min, String-like structure survives. d. Charred at 300 °C for 60 min, Detail of string-like structure with precipitates on surface of string-like structures. e. Charred at 600 °C for 60 min. Melting of structure providing a holey structure. f. Charred at 600 °C for 60 min. Detail of e showing melted sponge structure.

116.[97] “For if tears are running from the inner corner of the eye involuntarily. This disease is called by the doctors roias that is it is named after the flow. Treat these patients like this. That is take a new sponge (JCA_0200) and soak it in cedar oil (JCLP_083) and sprinkle the ashes (JCX_0126) of the sponge (JCA_0924) where the tears are flowing. 2. Again, take a new sponge (JCA_0200) and soak it in cedar oil (JCLP_083) and mix the ashes (JCX_0126) of the sponge (JCA_0924) once you have burnt it with the blood (JCX_0103) of a JCA_1717 and macerate it well and apply to the area where the tears are flowing. He shall not drink wine (JCLP_099) nor anything else that has a warm influence such as onions (JCLP_102) leek (JCLP_169) and the like, he shall not wash nor should the sun nor wind hit him.”

Dioscorides V, 120 σπόγγοι (*spongoi*), “Sponges have a wide variety of uses…. Sponges are burned like bastard sponges. Sponges that are empty and devoid of any fatty substance can treat wounds, repress swellings, glue fresh wounds with water or with water and vinegar, and with boiled honey glue old hollows” (Beck, 2005).

Many sponges are made of bio-silica whilst others are calcareous. It is uncertain what burning may do but perhaps this is to reduce the organic content and provide a fine ground glass. The sponge showed interesting changes during the charring process (Fig. 7). At 300 °C the complex porous nature of the sponge material is evident (Fig. 7a–d). At higher temperatures the sponge material begins to melt and gives rise to a brittle vacuous material (Fig. 7e, f).

All marine sponges contain iodine and it is this element that is likely to be why such materials are used (Sipkema et al., 2005) and have a beneficial effect. We have shown that the sponges, even when burned retain some of the porous properties but also are able to be dissolved and put a number of elements into solution, including iodine (52 mg L^−1^) in the 600 °C ash. Indeed with wine vinegar it is particularly effective, but our experiments show that iodine is especially taken up with the use of wine as a carrier liquid. We should not dismiss the concentration of strontium and boron in solution (Table 6) which may be of value in wound repair.

**Table 6:** Elemental analyses: Burnt substances.

Sample	Formula	Liquid	Element	Ca	Na	Mg	K	Fe	Al	Mn	B	Ba	Cd	Co	Cr	Cu	Li	Ni	Pb	Sr	Ti	V	Y	Zn	As
			Unit	mg l^−1^	mg l^−1^	mg l^−1^	mg l^−1^	mg l^−1^	mg l^−1^	mg l^−1^	mg l^−1^	mg l^−1^	mg l^−1^	mg l^−1^	mg l^−1^	mg l^−1^	mg l-1	mg l^−1^	mg l^−1^	mg l-1	mg l^−1^	mg l^−1^	mg l^−1^	mg l^−1^	mg l^−1^
Blank		**H2O**		0.0	0.0	0.0	0.0	0.00	0.00	0.00	0.000	0.000	0.000	0.000	0.000	0.000	0.00	0.000	0.000	0.000	0.000	0.000	0.000	0.000	0.000
Blank		**Wine**		46.8	0.0	74.8	902.4	4.0	0.0	0.9	4.7	0.1	0.0	0.0	0.0	0.3	0.0	0.0	0.2	0.3	0.0	0.1	0.0	0.8	0.0
Blank		**Wine vinegar**		108.7	28.3	51.4	533.1	1.5	0.4	0.9	3.1	0.1	0.0	0.0	0.0	0.6	0.0	0.0	0.2	1.0	0.0	0.0	0.00	0.8	0.00
**Gypsum**	CaSO_4_·2H_2_O	**H2O**		13,354.7	136.9	25.6	47.4	0.0	1.8	0.4	0.6	0.5	0.0	0.0	0.1	0.3	0.0	0.0	0.5	13.3	0.1	0.1	0.1	1.5	0.0
		**Wine**		7710.4	131.5	299.6	3711.6	86.3	0.0	23.8	17.8	1.5	0.3	0.0	0.0	3.7	0.0	0.4	4.7	7.4	0.0	1.5	0.0	8.8	7.6
		**Wine vinegar**		27,693.4	328.9	184.7	1917.7	18.1	7.6	7.9	23.5	1.9	0.0	0.0	0.2	2.3	0.0	0.3	1.6	24.7	0.0	0.2	0.0	2.5	0.0
**Hematite**	Fe_2_O_3_	**H2O**		19.6	18.8	0.5	9.4	17.3	0.0	1.0	0.4	0.4	0.0	0.0	0.0	0.6	0.0	0.0	1.1	0.3	0.0	0.1	0.0	0.1	0.3
		**Wine**		0.0	0.0	98.3	1760.8	138.6	0.0	19.9	9.2	4.2	0.0	0.0	0.1	19.4	0.0	1.3	5.5	0.3	0.0	1.6	0.0	7.2	0.0
		**Wine vinegar**		234.7	130.5	48.4	981.4	114.8	2.3	4.8	19.1	2.3	0.0	0.0	0.3	235.5	0.0	0.3	0.0	1.9	0.0	0.2	0.0	1.7	4.6
**Galena**	PbS	**H2O**		201.3	1.6	8.6	1.3	0.0	0.0	10.7	0.2	1.3	0.1	0.2	0.0	0.2	0.0	0.0	8.4	1.5	0.0	0.0	0.0	1.7	0.1
		**Wine**		722.2	3.9	128.6	1887.0	251.4	0.0	47.1	10.2	1.6	0.4	0.0	0.1	2.4	0.0	0.6	1162.0	3.9	0.0	1.3	0.0	11.0	0.0
		**Wine vinegar**		2258.8	104.7	291.8	1044.3	1271.6	7.1	129.4	21.3	1.5	0.5	0.2	0.1	4.5	0.0	0.4	2898.5	20.1	0.0	0.3	0.7	10.1	0.0
**Baryte**	BaSO_4_	**H2O**		113.6	32.7	0.0	1.3	0.0	0.0	1.0	0.2	9.2	0.0	0.0	0.0	0.3	0.0	0.0	0.8	10.6	0.0	0.1	0.0	0.1	0.0
		**Wine**		190.2	0.5	397.8	5421.6	91.3	0.0	25.2	25.4	108.9	0.3	0.0	0.0	5.4	0.0	0.4	27.3	2.4	0.0	2.7	0.0	8.9	0.0
		**Wine vinegar**		134.2	115.9	74.7	1191.9	9.0	0.7	0.0	21.1	12.2	0.0	0.0	0.1	2.0	0.0	0.1	20.5	0.0	0.0	0.1	0.0	0.9	0.2
**Calcite**	CaCO_3_	**H2O**		498.1	1.5	3.0	1.5	229.0	0.2	2.7	0.2	5.4	0.0	0.1	0.0	3.6	0.0	0.0	0.4	1.3	0.0	0.1	0.0	2.6	0.0
		**Wine**		7322.8	0.0	11.6	0.0	124.1	0.0	684.2	0.0	16.0	0.2	0.0	0.0	7.8	0.0	0.7	5.6	11.8	0.0	0.7	0.8	5.5	0.0
		**Wine vinegar**		244,017.2	0.0	1158.3	0.0	136.2	41.9	10,746.6	11.9	76.5	0.0	0.2	2.4	0.0	0.0	1.6	7.5	332.5	2.6	1.8	68.4	13.0	0.0
**Stock pyrite**	FeS_2_	**H2O**		301.1	31.8	60.9	27.8	1870.6	101.2	12.3	0.1	1.4	0.5	0.6	0.0	83.3	0.0	0.0	79.3	0.6	0.0	0.1	0.1	38.6	0.0
		**Wine**		82.1	0.0	300.5	3833.7	290.9	0.0	22.4	17.8	0.4	0.3	0.0	0.0	50.1	0.0	0.0	18.4	0.0	0.0	2.2	0.0	41.1	0.0
		**Wine vinegar**		325.3	178.3	182.9	1322.6	349.4	40.8	5.7	23.7	0.0	0.2	0.8	0.2	129.3	0.0	0.2	15.8	1.4	0.0	0.7	0.0	46.2	0.5
**Sphalerite**	ZnS	**H2O**		540.8	45.7	1.2	1.8	0.0	0.0	0.6	0.2	3.9	0.2	0.0	0.1	0.1	0.0	0.2	3.9	0.7	0.0	0.0	0.0	4.4	0.0
		**Wine**		1401.1	0.0	0.0	796.5	103.6	0.0	18.5	1.4	2.2	0.4	0.0	0.1	9.7	0.0	1.8	515.6	0.1	0.0	2.0	0.0	43.9	0.0
		**Wine vinegar**		3218.9	154.1	113.7	1463.5	70.5	4.6	2.7	24.2	1.6	1.3	0.2	0.3	326.6	0.0	1.2	1072.8	3.4	0.0	0.2	0.0	94.9	0.0
**Chromite**	(Mg, Fe) Cr_2_O_4_	**H2O**		78.4	12.7	20.2	6.6	9.5	3.4	0.4	0.5	0.4	0.0	0.0	2.4	1.3	0.0	0.0	0.9	0.2	0.1	0.1	0.0	0.2	0.0
		**Wine**		252.0	0.0	178.5	2918.9	273.6	0.0	23.1	10.9	1.6	0.1	0.3	2.1	26.1	0.0	0.2	3.5	0.3	0.0	1.8	0.0	9.3	0.0
		**Wine vinegar**		484.4	170.1	164.5	1536.0	357.4	19.4	60.0	24.5	1.5	0.1	0.3	5.7	110.6	0.0	2.5	1.7	3.2	0.0	0.3	0.0	4.2	0.0
**Oxidised pyrite**	FeS_2_/FeO (OH)	**H2O**		451.1	20.8	85.1	4.2	57,114.1	189.6	25.2	1.2	0.6	13.7	21.9	0.2	870.3	0.0	0.3	32.9	1.2	1.0	10.4	0.6	674.2	23.1
		**Wine**		532.2	24.8	909.9	12,485.6	76,110.0	145.4	48.9	48.5	2.3	16.4	22.1	0.0	922.0	0.0	0.0	17.0	2.7	0.0	7.2	0.0	679.9	19.9
		**Wine vinegar**		13.1	140.3	87.8	936.0	51,760.8	171.1	13.7	14.5	1.4	12.5	20.1	0.3	768.1	0.0	0.3	12.5	1.0	0.4	9.5	0.3	593.0	26.5
**Kalabras pyrite**	FeS_2_	**H2O**		56.8	5.7	12.6	49.1	793.3	85.7	5.0	0.4	1.2	0.1	0.4	0.0	55.6	0.0	0.1	58.6	0.3	0.0	0.1	0.0	3.2	0.0
		**Wine**		62.0	0.0	210.5	3904.8	275.6	10.6	23.1	13.8	0.5	0.3	0.0	0.0	37.7	0.0	0.1	46.7	0.2	0.0	1.6	0.0	9.9	0.0
		**Wine vinegar**		127.6	137.4	104.1	940.6	478.2	99.0	6.9	22.1	0.7	0.1	0.6	0.4	55.3	0.0	0.5	49.1	1.5	0.0	0.3	0.0	5.2	0.0
**Stibnite**	Sb_2_S_3_	**H2O**		902.8	5.9	2.9	6.4	16.1	0.4	1.3	0.3	0.9	0.0	0.0	0.0	0.2	0.0	0.0	0.1	1.1	0.0	0.1	0.0	0.6	0.0
		**Wine**		430.6	0.0	131.2	2720.9	271.7	0.0	21.0	9.3	1.9	0.2	0.0	0.2	1.4	0.0	1.5	29.6	0.9	0.0	1.8	0.0	8.1	0.0
		**Wine vinegar**		735.8	178.6	139.8	1362.5	345.6	14.0	1.1	26.3	3.1	0.1	0.2	0.9	0.7	0.0	0.4	22.1	4.4	0.0	0.3	0.0	4.9	0.1
**Molybdenite**	MoS_2_	**H2O**		54.0	35.1	0.9	20.0	4.3	0.1	2.5	0.2	1.6	0.0	0.0	0.0	0.6	0.0	0.0	0.1	0.2	0.0	0.1	0.1	1.0	0.0
		**Wine**		182.7	9.2	342.8	5941.0	109.9	0.0	23.3	20.7	2.1	0.3	0.0	0.0	6.5	0.0	0.6	9.0	1.0	0.0	1.8	0.0	10.2	1.1
		**Wine vinegar**		506.0	209.2	124.6	1145.6	204.6	4.8	5.5	23.4	1.6	0.1	6.0	0.5	5.4	0.0	0.8	13.3	2.9	0.0	0.1	0.0	4.0	5.3
**Chalcopyrite**	CuFeS_2_	**H2O**		140.1	14.6	33.6	1.9	7.8	0.4	8.9	0.2	0.3	0.3	1.2	0.0	134.4	0.0	0.0	0.5	0.3	0.0	0.0	0.0	87.0	0.0
		**Wine**		251.5	16.1	331.9	5064.9	238.4	0.0	27.4	19.4	1.8	0.0	0.0	0.2	231.1	0.0	1.1	9.6	0.9	0.0	1.5	0.0	61.4	3.2
		**Wine vinegar**		334.2	186.8	162.3	857.9	309.0	90.1	10.3	25.0	0.8	0.3	1.6	0.2	213.8	0.0	0.2	21.0	2.8	0.0	0.3	0.0	94.7	0.0
**Azurite**	CuOH (CO_3_)_2_	**H2O**		98.9	24.0	68.2	3.6	5.3	4.3	0.7	0.8	1.0	0.0	0.0	0.0	117.8	0.0	0.0	0.7	0.6	0.0	0.1	0.0	0.4	0.0
		**Wine**		277.1	0.0	281.8	1989.3	97.6	0.0	21.9	5.4	1.9	0.2	0.0	0.0	11,166.9	0.0	0.4	13.3	1.3	0.0	0.9	0.0	15.1	0.0
		**Wine vinegar**		205.6	91.0	210.6	47.3	85.8	80.2	11.2	13.6	3.0	0.0	0.4	0.0	63,392.1	0.0	0.3	106.5	2.5	0.5	0.3	1.5	146.7	0.0
**Umber**	Fe_2_O_3_ + MnO_2_ + nH_2_O + Si + AlO_3_	**H2O**		1302.3	851.4	215.2	35.6	0.0	5.1	2307.6	10.2	1.0	0.0	2.2	0.6	3.7	0.0	1.1	0.6	22.4	0.0	0.3	0.2	4.0	0.0
		**Wine**		1562.4	989.0	985.7	7921.8	438.1	19.9	20,096.2	40.2	8.4	0.2	30.6	5.8	22.4	0.0	11.2	2.8	134.6	3.0	11.5	10.1	17.0	1.5
		**Wine vinegar**		1832.9	1250.0	570.1	767.8	50.6	84.4	18,465.1	32.3	5.2	0.1	40.3	4.8	46.1	0.0	17.3	1.8	0.0	1.3	6.7	16.9	16.0	0.0
**Cuprite**	Cu_2_O	**H2O**		166.1	54.5	1.6	1.2	0.0	0.0	0.7	0.2	0.4	0.0	0.1	0.0	69.1	0.0	0.0	0.4	0.3	0.0	0.0	0.0	0.3	0.0
		**Wine**		90.1	0.0	140.9	3382.8	76.5	0.0	30.4	7.0	1.5	0.0	0.0	0.0	12,410.0	0.0	0.3	14.7	0.2	0.0	1.1	0.0	14.4	0.0
		**Wine vinegar**		0.0	54.4	0.0	592.8	69.2	8.2	18.6	12.0	1.4	0.0	1.7	0.0	67,733.7	0.0	0.7	127.1	0.0	0.5	0.0	0.0	184.3	0.0
**Orpiment**	As_2_S_3_	**H2O**		53.1	73.1	2.5	4.9	2.9	0.0	18.7	0.6	0.8	0.0	0.0	0.0	2.5	0.0	0.0	1.0	0.4	0.0	0.2	0.1	0.4	586.6
		**Wine**		144.9	0.0	403.8	7863.7	165.8	0.0	397.7	27.2	3.5	0.2	0.0	0.0	25.0	0.0	1.4	9.9	3.1	0.0	3.1	0.0	21.8	198.4
		**Wine Vinegar**		111.8	239.0	194.7	2597.1	70.9	15.2	20.7	42.4	2.9	0.0	0.0	0.5	711.3	0.0	0.8	2.8	3.8	0.0	0.3	0.0	7.7	395.3

#### 8.3.2 Plant material

Several recipes demand the burning of a range of plant material, but strangely not wood which would have been the most available material. Charring of plant material changes the chemical composition so that it becomes purer carbon (Scott, 2010). The main use of charcoal today is the use of activated charcoal that is used both internally and externally to adsorb material on to its surface (Bond, 2002). The activation of charcoal usually involves charring at least once with the addition of an activation agent or twice at two temperatures. This leads to a large number of pores in the charcoal that can increase the surface area of the charcoals. The recipes in JC utilise both olive and date stones that would have been a readily available waste material that was easily sourced, or leaves or roots of two of the most common plants (cypress and ivy).

##### 8.3.2.1 Date stones (Fig. 8)

JC 105.[86] “If the eye lashes fall out. Do this and they will grow. Burn the stone of a date (JCLP_222) and crush it finely and mix it with rose oil (JCLP_181) and apply. […]”

**Fig. 8:**
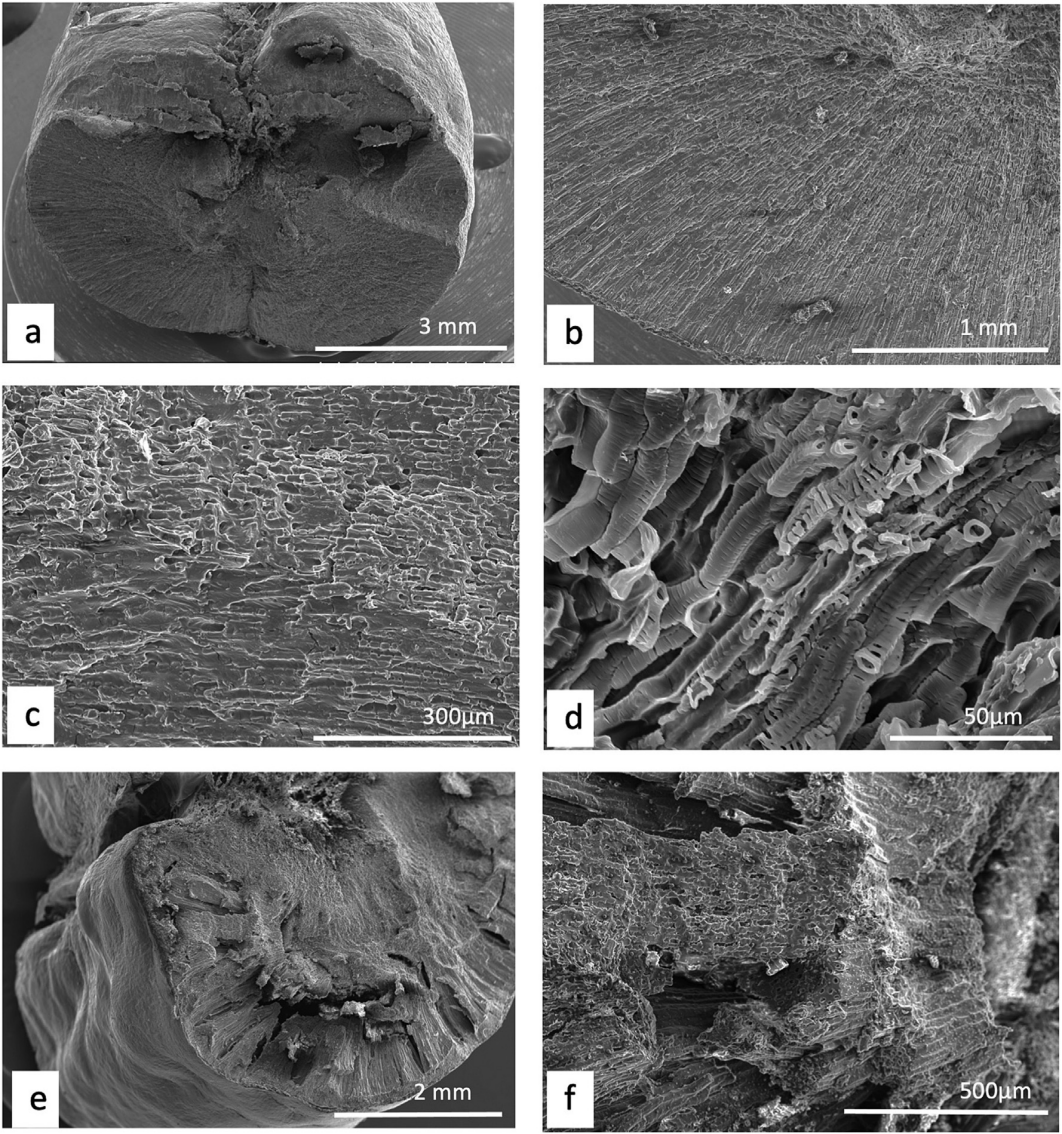

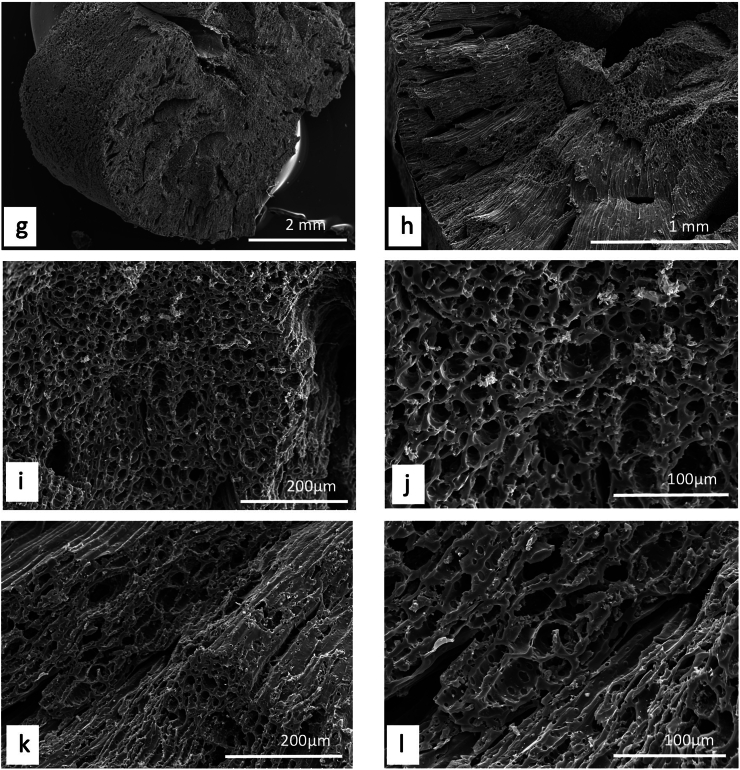
Date stones scanning electron micrographs. a. Uncharred endocarp showing internal seed. b. Section through uncharred endocarp showing fibrous structure. c. External surface of uncharred endocarp. d. Tubes in uncharred endocarp with spiral thickenings. e. Charred at 300 °C for 60 min. Section through endocarp and seed. f. Charred at 300 °C for 60 min Detail of endocarp showing fibrous structure. g. Charred at 600 °C for 60 min. Cross-section of endocarp and seed. h. Charred at 600 °C for 60 min. Detail of g. showing increased porosity of tissues. i. Charred at 600 °C for 60 min. Porosity in seed structure. j. Charred at 600 °C for 60 min. Detail of j. showing highly porous structure. k. Charred at 600 °C for 60 min. Structure of endocarp. l. Charred at 600 °C for 60 min. Detail of k showing highly porous endocarp tissue.

61.[54] For external haemorrhoids. “[…] 4. Burn the stones of a date (JCLP_222) and sprinkle their ashes (JCX_0126) but first wash with wine (JCLP_099). […]”

Several recipes call for the use of burnt date stones. It is not clear why date stone charcoal would be needed instead of, for example, wood charcoal. The change in chemistry during the charring process is the same, with increasing carbon content at higher temperatures (Scott, 2010). Higher temperatures also cause the material to become more brittle hence allowing it to be crushed more easily (Scott and Jones, 1991). However comparison of date (Fig. 8) charcoal structure and wood charcoal structure (Scott, 2010) indicates significant differences. One of these may relate to the shape of charcoal particles when the material is crushed, and another to the potentially different absorption characteristics of the two materials and this may be relevant to the use of the date stones. A key feature of the date stone is the presence of an internal seed within the hard outer endocarp (Fig. 8a). The two materials have distinctive differences in their structure as well as chemistry (Fig. 8b–d). On charring the outer stone layer begins to modify its structure, leading to fractures and some increase in porosity (Fig. 8e–f) that continues to increase with higher temperatures (Fig. 8g–l). The outer stony endocarp becomes more ‘spongy’ in structure, with many additional pores (Fig. 8l).

Date stones have been investigated for their activation properties. Experiments by Cherik and Louhab (2017) used zinc chloride to help with the activation process. Our experiments with date stones at both 300 °C and 600 °C indicate that a microporous structure (Fig. 8h–l) was achieved without the addition of zinc chloride leading to the conclusion that the structure of the charred date stone would have had already some activation. That the charred date material was used externally in some recipes remains unclear. Why for example that may have stimulated eyelash growth is unclear unless removal of surface or material within hair follicles was achieved. There is the potential that this treatment is used as a detoxifier (Mathew et al., 2018). Date stone ash dissolved in wine and wine vinegar (Table 6) produced high concentrations of a variety of dissolved elements and because of this it may have had a wide variety of effects.

##### 8.3.2.2 Olive stones (Fig. 9)

JC. 61. “8 Burn the stones of an olive (JCLP_053) and dry gourd (JCLP_092) and rub their ashes (JCX_0126) but first wash them with warm wine (JCLP_099).”.

**Fig. 9:**
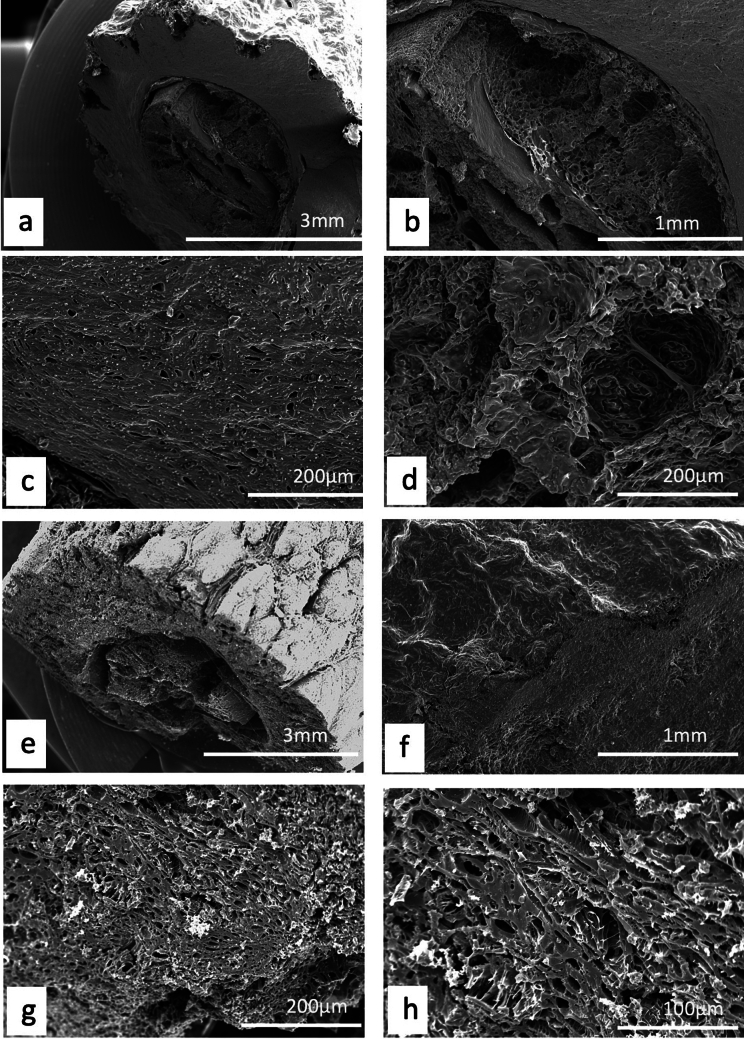
Olive stones scanning electron micrographs. a. Charred at 300 °C for 60 min. Broken stone showing internal soft kernel or seed and hard outside stone endocarp. b. Charred at 300 °C for 60 min. Detail of endocarp-seed boundary. c. Charred at 300 °C for 60 min. Longitudinal surface of seed. d. Charred at 300 °C for 60 min. Internal seed with porous structure. e. Charred at 600 °C for 60 min. Broken stone showing internal soft kernel or seed and hard outside stone endocarp. f. Charred at 600 °C for 60 min. Detail of outside of stone endocarp. g. Charred at 600 °C for 60 min. Stone endocarp showing porosity. h. Charred at 600 °C for 60 min. Detail of g.

As with the date stones several recipes call for the use of burnt olive stones. Just as with the date stones the structure of the charcoal produced from olive stones shows a large number of pores (Fig. 9h). Many of the changes seen in the date stones are also seen in the olive stones with, for example an increase in the range of hole sizes in the outer stony endocarp (Fig. 9g, h). It is difficult to see why either stone should be preferred other than availability.

It is interesting to note that olive stones are also used to produce charcoal and ash. Like the date stones, olive stones have also been used in activation studies using zinc chloride (Cherik and Louhab, 2018). The single charring at a range of temperatures up to 600 °C produces a highly porous charcoal (Fig. 9h) that on its own may have activation properties. As with date stones olive stones are recommended by JC as a cure for haemorrhoids: “Burn the stones of an olive (JCLP_053) and dry gourd (JCLP_092) and rub their ashes (JCX_0126) but first wash them with warm wine (JCLP_099). […].”

We note here that charred olive stones have not only been used as a fuel but are readily available in many archaeological sites and their structure has been used to interpret their use (Marinova et al., 2011). Olive stones produce a very porous charcoal both at the 300 °C and 600 °C temperatures. Our investigation of mixing the ashes with warm wine created a solution with a wide variety of elements (Table 6) and just as with the date stones may have had a wide variety of uses. High concentrations of strontium and boron may have helped in wound healing.

##### 8.3.2.3 Cypress leaves (Fig. 10)

Experiment 7. JC 61.[54] “For external hemorrhoids. […] 3. If they are not swollen, take the leaves of cypress (JCLP_106) and burn them and make them like flour. And then wash them with warm wine (JCLP_099) then rub the ground leaves on top.”.

**Fig. 10:**
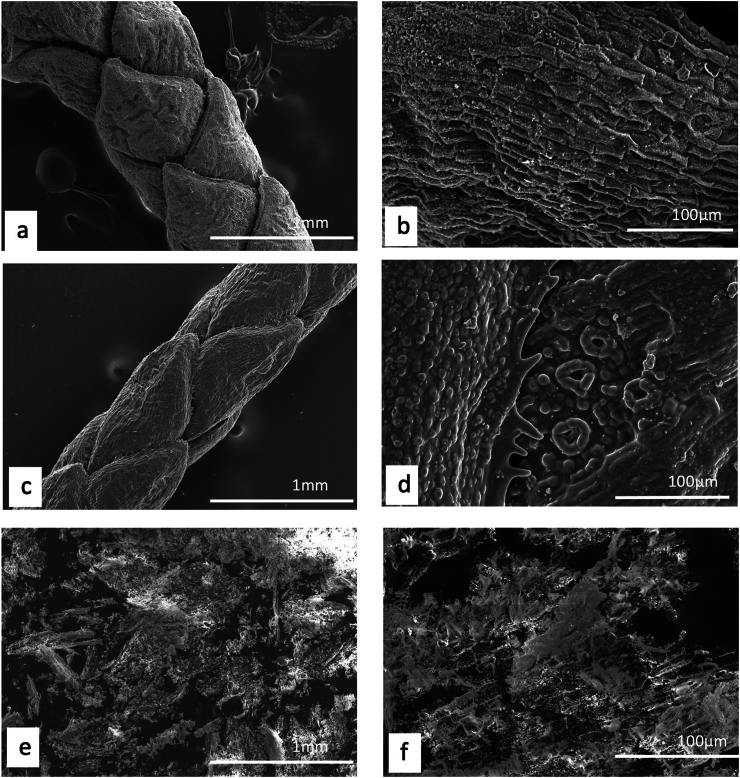
Cypress leaves. Scanning electron micrographs. a. Charred at 300 °C for 60 min, Twig with leaves attached. b. Charred at 300 °C for 60 min, Detail of leaf surface. c. Charred at 600 °C for 60 min. Twig with leaves attached. d. Charred at 600 °C for 60 min. Detail of stomatal surface on leaves and leaf margin. e. Ashed at 600 °C for 4 h. Ash residue with charcoal and mineral matter. f. Ashed at 600 °C for 4 h. Detail of ash residue with charcoal and mineral matter.

Dioscorides 1,74 κυπάρισσος (*kuparissos*), *Cupressus sempervirens* L., cypress

Dioscorides considers a number of preparation methods and uses. He writes for example: “The cypress binds and cools. Its leaves, when drunk with grape syrup and with a small amount of myrrh, are good for bladder incontinence and for difficult micturition. Its small, pale-green berries chopped up and taken in a drink with wine are good for coughing up blood, dysentery, diarrhoea, orthopnoea, and coughs. … The leaves, ground up and plastered over wounds, mend them and they are antihemorrhagic, and when triturated with vinegar, they dye hair” (Beck, 2005).

It is unclear in this case why cypress leaves are required rather than the leaves of any other plant. It is possible that the smell and release of any resins may have been seen to have been significant, but also the way in which the ashes may have been easily ground. It is possible that the material used is not only charcoal but the ash that may contain a number of organic and inorganic materials. The charring of the cypress leaves both at 300 °C (Fig. 10a, b) and 600 °C (Fig. 10c, d) showed the external anatomy of the leaves beautifully preserved. Some of the material was burned in oxygen at 600 °C for 4 h (Fig. 10e, f) that left an ash residue with little charcoal with little anatomical structure left. This material was used in the dissolving experiment.

It is not clear why cypress leaves are burned. It is possible that this is because their leaves are available all year round. It is also possible that the leaves have other uses when uncharred, as they may contain several organic chemicals, but their use charred would make some sense to JC. Our observations indicate that the structure of the charcoal may not have had any significance. However, cypress ash when dissolved in wine vinegar produces a solution with a large number of elements (Table 6) so that several may have had a therapeutic effect.

##### 8.3.2.4 Ivy roots (Fig. 11)

JC 170.[137] “It softens joint pain and hardening. […] 5. Burn the root of ivy (JCLP_087) and mix ashes (JCX_0126) with yolk (JCX_0114) of an egg (JCA_0105) and put it onto the pain. […]”

**Fig. 11:**
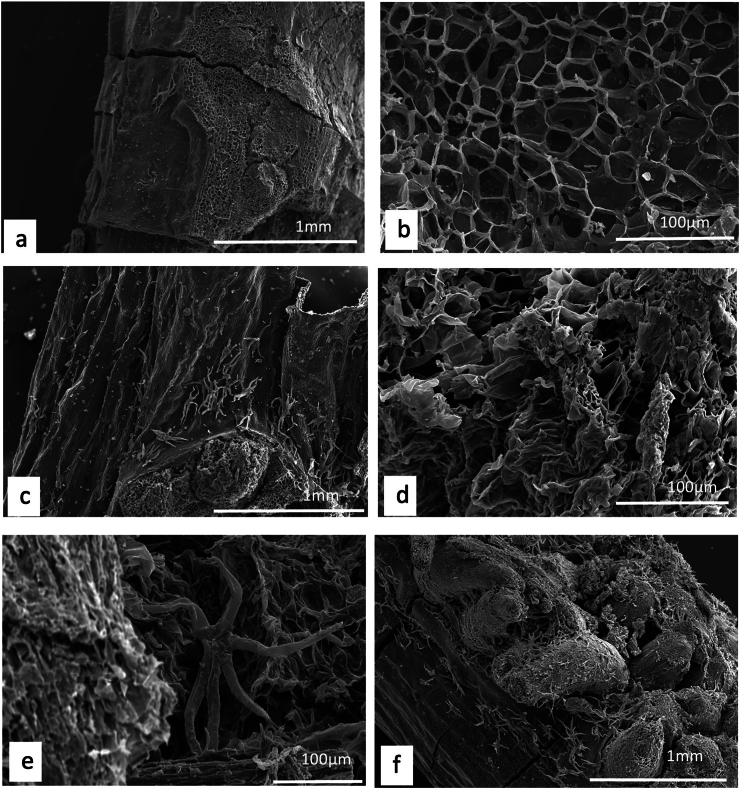
Ivy roots. Scanning electron micrographs. a. Charred at 300 °C for 60 min. External view showing porous nature of internal structure. b. Charred at 300 °C for 60 min. Detail of porous internal structure. c. Charred at 300 °C for 60 min. External surface of root with suckers and tendrils. d. Charred at 300 °C for 60 min. Porous internal structure. e. Charred at 300 °C for 60 min. Star shaped root sucker. f. Charred at 600 °C for 60 min. Root tendrils and suckers visible on roots.

Ivy (*Hedera helix* L.) has had a long history for use in medicines. Several uses are mentioned in Dioscorides. Dioscorides II, 179 κισσός (*kissos*), “All ivies are pungent, astringent, and act on the nervous system. A pinch of their flowers drunk with wine is good for dysentery, but one must take the drink twice a day; they are also suitable for burns when triturated with a cerate…. The leaves that are tender, boiled with vinegar or triturated raw, treat the spleen; the juice of the leaves and of the berry clusters is poured into the nose with unguent of iris or honey or soda for chronic headaches” (Beck, 2005). He goes on to add: “The berries, ground up and drunk or burned so as to produce smoke from below, set the menses going and an amount of one *drachma* taken in a drink at the end of the menstrual period causes barrenness” (Beck, 2005).

However it is not immediately clear why burning ivy roots would have been of particular interest. It could simply be that the plant was easily identifiable and easy to obtain.

We observed good charcoalification of the ivy roots at 300 °C (Fig. 11a–e) and at 600 °C (Fig. 11f). Given that the charring process preserved the anatomy of the plant it is not immediately obvious the reason for using this material as a starting material. Star-like suckers are beautifully preserved (Fig. 11e, f) and it maybe this feature that was considered significant.

Here the ashes of the ivy are mixed with egg yolk, possibly being kinder to the skin. Their use relates to pain relief. In Dioscorides it is unburnt ivy that is used suggesting that it is some of the organic chemical content of the leaves that is significant. It is not clear from our experiments why burning of the ivy roots is significant. Mixing of uncharred leaves may release a number of potentially useful organic chemicals that may play a role in pain relief. We know that the pro-drug (pharmacologically inactive precursor) of the active ingredient of the pain killer aspirin is salicin that is found in willow bark and myrtle (Montinari et al., 2019) but it is not recorded from ivy roots or leaves. The structure of the charcoal may be significant in that star shapes can be well seen (Fig. 11e). However, the high concentration of boron and strontium (Table 6) may have played a role in helping treat several of the conditions such as toothache and reproduction issues.

#### 8.3.3 Coal, charcoal and coal tar

Coal and tar products have long been used for a range of dermatological conditions even today (Paghdal and Schwartz, 2009). It is interesting to note that in JC this kind of material is also recommended for such use: “229. Alopecia is if the hair of the head falls out. Do this treatment. Burn the leaves of reed (JCLP_075) and mix them with liquid pitch (JCLP_244) and lard (JCA_0077) of a sheep (JCA_0709) and shave the entire head and apply. […].”

Charcoal has also been used mixed with honey in oral treatments just as they have been today (Root-Bernstein and Root-Bernstein, 1999).

“233.[169,170] Crush 15 kernels of pepper (JCLP_154) and burn carob (JCLP_139) and crush one *hexagion* of the peel of pomegranate (JCLP_182) and one ink-gall (JCLP_085) without a hole and mix it with honey (JCA_0172) and apply to the area that is rotten. 2. Apply good (JCX_1217). 3. Mix fine yellow orpiment (JCLM_006) with honey (JCA_0172) and apply hemp (JCLP_205) and put hemp (JCLP_205) with your finger into the mouth where the rot is. 4 Burn the bark of willow (JCLP_072) and sheet of paper (JCLP_230) and their ashes (JCX_0126), add also fine pepper (JCLP_154) and make it smooth with honey (JCA_0172) and put it on the rot. […].” We note that other, as yet, unidentified plants can also be treated in the same way: “34.[31] When the mouth smells. Burn dry roses (JCLP_181) and make them like flour and rub it on the teeth. […]”

29.[26] “To make the teeth white. Burn horn (JCA_0502) of a deer (JCA_0366) until it gets white. Then grind it and make it like flour and apply to the teeth. […]”

Activated charcoal has also been used in oral hygiene/whitening recipes today (Brooks et al., 2017a, Brooks et al., 2017b). We note that in our experiments the material used suggests that some degree of activation is achieved indicating that adsorption and possible absorption are the main uses of the material.

### 8.4 Salts and dissolved minerals

Salts tend to create a number of problems today (Borrelli et al., 2020). These include too much salt linked to heart disease. We note also that sodium, potassium and magnesium are taken up from many of the plant and animal ashes (Table 6) that may have some use in providing supplements.

#### 8.4.1 Results of dissolving experiments

We have shown that most minerals do not dissolve well in water, wine or wine vinegar with the exception of gypsum, baryte and calcite as well as oxidised sulphide minerals (Table 7).

**Table 7:** Elemental analyses: Minerals.

Sample	Liquid	Element	Ca	Na	Mg	K	Fe	Al	Mn	B	Ba	Cd	Co	Cr	Cu	Li	Ni	Pb	Sr	Ti	V	Y	Zn	As
		Unit	mg l^−1^	mg l^−1^	mg l^−1^	mg l^−1^	mg l^−1^	mg l^−1^	mg l^−1^	mg l^−1^	mg l^−1^	mg l^−1^	mg l^−1^	mg l^−1^	mg l^−1^	mg l-1	mg l^−1^	mg l^−1^	mg l-1	mg l^−1^	mg l^−1^	mg l^−1^	mg l^−1^	mg l^−1^
Blank	**H2O**		0.0	0.0	0.0	0.0	0.00	0.00	0.00	0.000	0.000	0.000	0.000	0.000	0.000	0.00	0.000	0.000	0.000	0.000	0.000	0.000	0.000	0.000
Blank	**Wine**		46.8	0.0	74.8	902.4	4.0	0.0	0.9	4.7	0.1	0.0	0.0	0.0	0.3	0.0	0.0	0.2	0.3	0.0	0.1	0.0	0.8	0.0
Blank	**Wine vinegar**		108.7	28.3	51.4	533.1	1.5	0.4	0.9	3.1	0.1	0.0	0.0	0.0	0.6	0.0	0.0	0.2	1.0	0.0	0.0	0.00	0.8	0.00
**Charred materials**																								
**Mussel shell ash 600 DW**	**H2O**		34.2	1645.9	9.5	12.0	0.3	0.1	0.1	1.8	0.0	0.0	0.0	0.0	0.4	0.0	0.0	0.4	0.7	0.0	0.1	0.0	0.0	0.0
	**Wine**		10,540.6	1308.4	12.7	1694.3	50.9	0.0	18.2	2.4	1.3	0.1	0.0	0.0	4.3	0.0	1.2	4.4	38.6	0.0	0.0	0.0	1.9	0.0
	**Wine vinegar**		389,264.0	4358.5	1017.3	1925.5	7.2	18.0	8.6	44.9	3.7	0.0	0.0	0.5	2.8	0.0	2.4	8.4	890.6	1.1	0.9	0.8	1.8	0.0
**Mussel shell ash 900 DW**	**H2O**		46,073.0	1566.5	0.0	26.3	0.0	7.6	0.5	6.5	23.8	0.0	0.0	0.2	2.3	0.0	0.0	3.7	192.4	0.8	0.1	0.5	4.7	0.0
	**Wine**		52,372.0	1894.3	1205.5	31,902.1	1.1	0.0	0.4	80.5	0.0	0.4	0.0	0.0	2.4	0.0	6.5	14.6	114.2	0.0	0.1	0.0	0.4	4.4
	**Wine vinegar**		421,966.8	2440.1	455.0	1817.1	35.9	38.1	7.2	41.8	4.1	0.0	0.0	0.3	10.9	0.0	1.4	17.1	804.7	0.5	0.7	1.1	4.2	0.0
**Egg shell ash 600 DW**	**H2O**		811.4	0.0	114.5	36.1	0.0	0.1	0.1	0.5	0.5	0.0	0.1	0.1	0.9	0.0	0.0	2.9	0.8	0.1	0.0	0.0	0.4	0.0
	**Wine**		31,628.0	22.0	958.6	9288.7	192.4	0.0	54.2	26.2	7.1	0.2	0.0	0.0	12.1	0.0	1.7	14.6	16.7	0.0	3.0	0.0	18.3	0.0
	**Wine vinegar**		2513.3	820.7	2128.5	14,741.6	142.2	49.7	27.3	98.6	5.4	0.0	0.0	1.0	7.9	0.0	1.4	1.2	15.9	0.0	0.6	0.0	36.6	0.0
**Egg shell ash 900 DW**	**H2O**		58,014.9	457.3	4.2	132.0	0.0	7.6	0.5	0.1	12.5	0.0	0.0	0.2	1.0	0.0	0.3	2.1	84.4	0.9	0.4	0.5	5.5	0.0
	**Wine**		58,507.5	415.5	2329.5	32,604.3	0.6	0.0	0.0	72.1	0.0	0.0	0.4	0.0	0.0	0.0	3.5	1.6	27.2	0.0	0.0	0.0	0.0	15.9
	**Wine vinegar**		421,940.3	1249.9	2580.4	1149.3	63.6	47.4	2.4	37.1	39.1	0.0	0.0	0.8	8.1	0.0	0.0	2.3	229.0	0.7	0.7	1.1	6.2	2.7
**Deer horn 900 DW**	**H2O**		250.6	564.8	865.8	267.9	0.7	0.2	0.1	0.3	0.4	0.0	0.0	0.0	2.9	0.0	0.0	0.6	0.1	0.0	0.2	0.0	0.1	0.0
	**Wine**		1427.3	6423.1	7780.0	10,579.7	53.7	0.0	12.8	38.3	17.7	0.1	0.0	0.0	5.3	0.0	2.2	2.1	3.8	0.0	0.0	0.0	8.2	0.0
	**Wine vinegar**		45,930.3	7406.0	5252.6	712.8	0.0	0.0	2.1	21.8	49.9	0.0	0.1	1.1	2.8	0.0	0.4	0.3	37.5	0.0	1.4	0.0	0.0	0.0
**Cypress ash 600 DW**	**H2O**		3082.6	7042.6	2934.4	25,134.0	19.6	7.2	6.3	52.7	8.2	0.0	0.0	0.3	1.8	0.0	0.0	4.3	25.6	0.2	0.9	0.2	1.7	3.9
	**Wine**		102,623.4	6033.4	19,078.1	74,724.0	840.8	0.0	1073.3	265.2	198.1	3.7	0.0	0.0	60.9	0.0	5.3	53.7	324.7	0.0	7.4	0.0	168.0	0.0
	**Wine vinegar**		360,207.5	11,924.0	16,766.4	24,248.4	769.4	961.8	1229.9	491.6	588.4	0.0	1.4	5.0	149.2	0.0	14.9	7.3	962.8	6.5	6.8	2.9	160.0	0.0
**Cypress ash 900 DW**	**H2O**		336,325.3	701.3	2030.4	3158.2	69.7	90.7	94.9	61.0	278.3	1.9	2.4	6.7	5.6	0.0	1.0	23.9	234.6	6.6	3.5	3.7	39.5	0.0
	**Wine**		147,311.6	3352.4	25,045.6	89,990.7	1829.0	0.0	1563.7	433.6	182.1	2.3	0.0	0.6	1472.6	0.0	17.1	108.1	156.7	0.0	21.1	0.0	162.4	46.2
	**Wine vinegar**		518,571.1	8686.4	32,861.5	45,484.6	1353.5	1750.8	1827.2	559.9	516.5	0.1	3.9	13.8	268.1	0.0	19.6	40.6	716.3	20.4	4.8	1.5	110.9	0.0
**Date stone ash 600 DW**	**H2O**		210.4	29.0	1017.8	8967.6	2.2	3.1	1.8	7.8	0.0	0.0	0.0	0.0	0.8	0.0	0.0	1.3	0.7	0.1	0.5	0.0	1.8	0.0
	**Wine**		21,242.4	2250.4	59,756.7	390,504.5	3188.0	0.0	998.4	1087.3	52.4	6.3	0.0	0.0	405.8	0.0	24.2	86.0	111.3	0.0	37.5	0.0	782.0	0.6
	**Wine vinegar**		462,392.6	2044.1	3120.9	0.0	88.6	49.5	4.5	63.5	27.9	0.0	0.1	0.9	1.7	0.0	1.2	2.5	227.6	0.0	1.0	0.9	9.0	0.0
**Date stone 900 DW**	**H2O**		117.7	3698.2	2902.9	248,936.2	9.9	8.2	6.6	101.6	0.0	0.0	0.3	0.9	135.5	2.6	1.8	0.0	0.4	0.0	0.4	0.0	22.1	15.9
	**Wine**		21,456.1	1731.1	68,036.7	479,368.4	4233.1	0.0	1363.7	1151.1	73.1	0.0	0.0	7.0	558.7	0.0	9.9	128.5	117.4	0.0	81.3	0.0	939.7	0.0
	**Wine vinegar**		34,725.1	7184.9	75,575.6	316,197.9	763.4	164.9	765.3	569.2	15.3	0.5	0.0	12.0	352.8	0.0	39.4	8.1	207.7	0.0	0.0	0.0	607.2	0.0
**Ivy ash 900 DW**	**H2O**		9733.3	2755.4	12.1	48,995.3	0.0	307.4	0.5	36.0	5.4	0.6	0.0	8.9	1.2	1.7	0.0	2.7	15.0	0.0	3.1	0.4	2.7	0.0
	**Wine**		29,430.2	11,085.7	19,714.0	175,310.2	946.0	543.4	527.3	633.9	182.6	2.2	0.0	5.5	251.4	0.0	10.2	0.0	215.3	0.0	32.0	0.0	202.7	53.5
	**Wine vinegar**		115,261.0	9631.5	15,107.8	54,645.0	881.7	3097.7	395.9	646.2	277.0	0.0	1.2	23.6	152.8	0.0	15.2	0.6	456.3	89.6	21.0	0.0	137.9	8.6
**Sponge ash 600 DW**	**H2O**		17,713.6	6095.8	5440.8	622.1	207.0	43.9	2.4	107.9	12.1	0.2	0.0	0.8	10.1	0.0	0.0	17.4	480.1	0.0	0.4	0.0	8.3	15.5
	**Wine**		172,550.6	6536.3	52,644.1	272,007.3	7314.0	3532.4	896.5	1630.6	109.3	8.6	0.0	15.7	282.1	0.0	22.5	741.4	1857.2	0.0	96.5	0.0	438.2	0.0
	**Wine vinegar**		192,589.1	19,424.3	31,605.6	0.0	6812.4	7125.7	183.3	1542.8	107.0	0.0	2.9	32.3	203.7	0.0	31.2	3.3	2807.5	170.4	38.2	0.0	181.6	1.3

Many of the recipes require the dissolving of the mineral or burnt substances with usually wine vinegar. We therefore tested the effectiveness of dissolving elements in wine, wine vinegar and water. The use of rose oil (made by the maceration of rose petals with virgin olive oil) is more difficult to investigate as different analytical techniques are involved in its analysis that were not available to the authors.

##### 8.4.1.1 Dissolving in water

200.[152] “If they beat each other and develop wounds. […] 2. Grind the so-called Lemnian earth (JCLM_017) and crush and macerate with water (JCLM_022) and apply. 3. Crush lump of earth (JCLM_009) and macerate with water (JCLM_022) and put it onto the wound. […]”

For the most part many minerals do not generally react with neutral pH water. When they do it is either because the water may be slightly acidic (*e.g.,* draining from old mines or workings) or the mineral concerned may be more easily dissolved over a long time, such as with some carbonate minerals. It is important to mention that only distilled water was used to avoid input from the water itself. In our experiments calcium is most easily released from gypsum (Fig. 2j), which is a calcium sulphate (Table 7). This is of no surprise as the mineral may often be formed from the weathering of clays. Indeed calcium appears to be the element most soluble and consequently easily extracted from several minerals with water, even more so if the mineral concerned, such as umber or oxidised iron pyrite has already undergone a weathering process.

It is therefore no surprise that at least one mineral (*e.g.,* Lemnian earth) may be macerated with water and this may also provide a clue to the identity of other potential minerals, as yet unrecognised in the ancient texts.

What was more of a surprise was the ease that a number of elements were released from several burnt substances simply by the addition of water (Table 7). Not only were calcium, magnesium and potassium and even iron and aluminium found with simple water addition for many of the materials but even strontium and boron, and in some cases copper, were often found to be enriched in the solution.

##### 8.4.1.2 Dissolving in wine

The wine may already possess naturally high concentrations of some elements (*e.g.,* calcium, magnesium and potassium seen in the wine blank) but our data tables (Table 6, Table 7, Supplementary publication) look at the increase in these elements when in contact with the different materials (*i.e.,* the original wine concentrations are subtracted). Wine is particularly efficient in releasing elements into solution, most likely due to its low pH, typically between 3 and 4. We note that elements such as calcium, sodium, magnesium and potassium may be released from a variety of minerals. Also, oxidised pyrite, for example, releases iron, boron, cadmium, copper, lead and zinc. All these may provide a range of effects if ingested. This is also the case with many of the burnt substances and occasionally the solution is even more dramatic. Again calcium, sodium and potassium are often seen but also in some cases, such as in the mussel shells concentrations are raised in the material experiencing the higher temperatures such as magnesium, boron and strontium.

Sponge ash yielded increased concentrations of a wide range of elements that included calcium, sodium, magnesium and potassium (as did date stone ash), iron, aluminium, barium, boron but also copper lead, strontium and zinc. There was only one example of obtaining iodine from burnt sponges (53.23 mg l^−1^). This ability of wine to dissolve a significant number of elements from burnt substances may explain its widespread use.

##### 8.4.1.3 Dissolving in wine vinegar

Many recipes which advocate the use of wine (see Section 8.4.1.2 above) also involve the use of wine vinegar. The use of wine vinegar may be seen as a more obvious choice to at least dissolve some minerals to release their elements, with typical pH between 2.5 and 3, but this is likely only to have a significant impact with sulphates and carbonates. The wine vinegar is particularly effective with gypsum (Fig. 2j) releasing high concentrations of calcium, sodium, magnesium and potassium and with calcite (Fig. 2l) which also yielded high concentrations of manganese and strontium and even yttrium (Table 7). Unsurprisingly hematite (Fig. 2r) produced some iron, galena (Fig. 2m) produced lead, sphalerite (Fig. 2n) produced zinc *etc.,* (Table 7), but it is the oxidised pyrite (Fig. 2d, e) that provided the widest range of elements but not as much as with the wine alone (Table 7). With cuprite (Fig. 2s), however, wine vinegar was more effective that wine in putting copper into solution but in addition, lead and zinc. One surprising result was that water was more effective in dissolving arsenic than either wine or wine vinegar. We note also with the burnt substances there may be significant differences between the uses of wine and wine vinegar with wine vinegar being particularly efficient in dissolving calcium from a range of minerals but also unexpectedly elements such as strontium, zinc, copper, barium, boron, manganese, iron, aluminium, potassium magnesium, and sodium that may possibly explain some usages (Table 6, Table 7).

## 9 Discussion

### 9.1 Minerals

Most often minerals have been identified or at least described using a combination of colour and form. Unfortunately, neither feature can provide definitive identification (Zipser et al., 2023b). Under modern systems minerals are described and named using a combination of chemical composition and mineralogical structure — neither of which can be undertaken by a non-specialist. Another aspect of minerals that needs consideration is that they are composed of elements, and it is these that are the key to the potential medicinal use (Fig. 1). We know that many major elements found in minerals such as iron, copper (*e.g.,* Borkow and Gabbay, 2009) and zinc, for example, are essential for human health but also cause potential problems when available in excess (Mason, 1979; Selinus, 2005) (see section 5). However, it is also more minor elements or trace elements that may represent the key ingredients, such as selenium, potassium, sodium, or even cadmium or lithium (Selinus, 2005; Seidel et al., 2019). The use of colour to identify a mineral can prove problematic as often it is assumed, for example, that copper minerals are blue (such as azurite) but that is not always the case as one ore, chalcopyrite has a brassy colour (Fig. 2b) and a major zinc mineral (Smithsonite) as well as hemimorphite (Fig. 2v) can both be blue. Equally many different minerals have a similar form, and others have a different form, for example massive or botryoidal.

We have used two approaches to the identification of a mineral. One is to use data on occurrence. There is extensive data on the distribution of minerals that is now readily available online and this may provide important clues (https://www.mindat.org/). In Dioscorides, for example, a range of localities is given for minerals, ordered in terms of quality. One issue that needs consideration is that the materials being described are in fact different minerals from different places and not a single mineral species (Zipser et al., 2023b). We have used the example of lapis lazuli that is cited by Dioscorides to occur in Cyprus (Osbaldeston and Wood, 2000). However, neither this material nor others such as chrysocolla (Zipser et al., 2023b) occur in Cyprus (Davies, 1930; Constantinou and Govett, 1973; Edwards et al., 2010; Antivachis, 2015). In addition, lapis lazuli is a rock type, mainly from Afghanistan and the blue mineral may be complex and variable. It is probable that the rock may have been prized and sold for high prices but misidentified and a range of blue minerals substituted.

In the second approach, we consider the potential role of a mineral is being used to treat an external or internal condition. The key here is how to make the important element bioavailable so the body can use it (Selinus, 2005). How the mineral is prepared may help in this regard. Common silicate minerals are not easily dissolved and hence may have little use for internal treatments whereas carbonates, sulphides or sulphates can be dissolved in dilute acids such as vinegar, and even wine to some extent.

Whilst there is extensive literature concerning mineral species in ancient medicines (Lev, 2010; Duffin et al., 2013), we need to reassess their identification in many cases. There has also been considerable interest in the use of clays or earths that are formed as a result of rock weathering for their potential medicinal use (Williams and Haydel, 2010; Williams, 2019). Most well-known are the Samian earths that have been prized for their medicinal uses (Photos-Jones et al., 2015). However recent research suggests many different clays or earths have medicinal properties, especially antibacterial properties of some volcanically derived clays and so geographical and geological occurrence may be a clue (Williams and Haydel, 2010; Williams, 2019). This may also be the case with natural or spring waters where some elements are found enriched and easily bioavailable such as those containing the element lithium (Seidel et al., 2019).

Many of the recipes involve the use of liquids to dissolve mineral substances. In our experiments we have shown that attempting to dissolve a range of likely minerals is problematic in all mediums, but we note the significance of using naturally oxidised materials as a base material that can easily be dissolved in water.

### 9.2 Burnt substances

When considering burnt substances we need to consider two types — burnt plants and burnt animals and whether it is the smoke or ashes that are the key part. Burning plants may provide a smoke with a particular smell that may be considered therapeutic. However, we should not forget that some plants preferentially take up some elements from the soil (Christou et al., 2017) and hence burning them may make these elements bioavailable in either the smoke or the ashes in particular (Brooks, 1994; Davies, 1994; Farago, 1994; Mehra and Fargo, 1994). We should also not forget that some smokes may contain carcinogens, harmful to people — they may also cause abnormalities in the babies of pregnant women (Scott, 2020; Foo et al., 2024). The ash may also contain charcoal that may have several uses (Scott and Damblon, 2010; James et al., 2005). Activated charcoal may be used internally to absorb a range of elements (Bond, 2002; Brooks et al., 2017a) but there are known side-effects (Elliott et al., 1989). The structure of olive and date stones when charred may provide some activation and hence their widespread use (Cherik and Louhab, 2017, Cherik and Louhab, 2018; Mallick et al., 2019). We note that a two-stage process has been devised for charcoal activation (*e.g.,* Cherik and Louhab, 2017) but our experiments suggest that this may not be necessary and that the charring of date and olive stones alone may create activated charcoal and hence its use by JC.

The burning of animals may be much more complex. We have shown that burning cuttlefish bones provided small blades that may have a use when suspended in honey. Honey is well-known to have antibacterial properties (Ramsay et al., 2019). In some cases sponges have been burned. Recent research has shown that some commonly burnt sponges contain iodine and could be used to stem blood flow (Sipkema et al., 2005). The key in this category may be to undertake experiments to see the result of the described process to help in the identification of the material. However, in the case of sponges it appears that all sponges may contain a wide variety of different chemicals that have a range of medicinal properties (Sipkema et al., 2005) and this is supported by our experiments.

## 10 Conclusions

We have translated John the Physician's terms for minerals and burnt substances and compared the interpretation with the ancient medicinal text of Dioscorides *De Materia Medica* (Beck, 2005). It became clear that many of the minerals that occurred in the translation of this text may not represent minerals of the same name in current geological literature. An analysis of the Dioscorides text showed that a large number of occurrences of each mineral are given (Zipser et al., 2023b). The text of JC is based in Cyprus and an analysis of the minerals occurring in Cyprus was undertaken. It became clear that many of the minerals claimed to be from Cyprus in the Dioscorides translations are not found in that country. Our analyses suggest that few of the mineral names found in ancient texts can be relied upon with regard to modern names and that many minerals may in fact be a range of different minerals that may easily be mistaken for each other.

We also, where we considered our identification correct, undertook a series of experiments to test some of the recipes of JC. We undertook charring and ashing experiments, observing physical changes using scanning electron microscopy. We were able to show that some of the transformations may have been beneficial for the recipe concerned. In addition, we experimented with dissolving minerals and burnt materials in common liquids used in JC such as water, wine and wine vinegar. Some of the solutions produced a wide range of elements and the results of our experiments reveal that some of the recipes may have provided a beneficial therapeutic effect and by the fact that some of the charred materials provided an adsorbent capacity and that some of the materials used such as honey, sponges and clays provided a range of antibacterial or other medicinal properties.

## CRediT authorship contribution statement

**Andrew C. Scott:** Writing – review & editing, Writing – original draft, Resources, Methodology, Investigation, Funding acquisition, Data curation, Conceptualization. **Rebecca Lazarou:** Writing – review & editing, Methodology, Formal analysis, Data curation. **Robert Allkin:** Writing – review & editing, Funding acquisition. **Mark Nesbitt:** Writing – review & editing, Supervision, Resources, Funding acquisition. **Andreas Lardos:** Writing – review & editing, Funding acquisition. **Efraim Lev:** Writing – review & editing, Funding acquisition. **Sharon Gibbons:** Investigation. **Nathalie, V. Grassineau:** Investigation. **James Brakeley:** Formal analysis. **Barbara Zipser:** Writing – review & editing, Project administration, Funding acquisition, Data curation, Conceptualization.

## Declaration of competing interest

The authors declare that they have no known competing financial interests or personal relationships that could have appeared to influence the work reported in this paper.

## Appendix A Supplementary data

Supplementary material Image 1
